# Supplementary material for: Cardiovascular risk prediction using physical performance measures in COPD: results from a multicentre observational study
Source: BMJ Open. 2020 Dec 28;10(12):e038360. doi: 10.1136/bmjopen-2020-038360 (PMC7772292; doi:10.1136/bmjopen-2020-038360)
Supplement: Supplementary data [file bmjopen-2020-038360supp001.pdf]

Supplementary materials for "Cardiovascular risk prediction using physical performance measures in COPD: results from a multi-centre observational study."

Jilles M. Fermont, Marie Fisk, Charlotte E. Bolton, William Macnee, John Cockcroft, Jonathan Fuld, Joseph Cheriyan, Divya Mohan, Kaisa M. Mäki-Petäjä, Ali B.A.K. Al-Hadithi, Ruth Tal-Singer, Hana Müllerova, Michael I. Polkey, Angela M. Wood, Carmel McEniery, Ian B. Wilkinson, on behalf of the ERICA consortium.

Fermont JM, *et al. BMJ Open* 2021; 10:e038360. doi: 10.1136/bmjopen-2020-038360

Supplementary text

Text 1 Supplementary methods

There were missing values. Data were assessed for the level and type of missing data, and completion patterns (Figures 1-2). There were about 10% missing values for variables CIMT (n = 66) and PWV (n = 60), with <5% missing values for other variables (Figures 3-5). Missing values were addressed using multiple imputations using chained equations (MICE). The time-to-event outcome was included using the non-parametric Nelson-Aalen estimator. Predictive mean matching was used for continuous variables, ordered logistic regression (as continuous) for ordinal variables, multinomial logistic regression for categorical variables, and logistic regression for binary variables. Derived variables such as the BODE Index (a composite score of BMI, forced expiratory volume in one second (FEV_1), Medical Research Council dyspnoea score, and 6MWT distance) and GOLD stage were estimated post MICE using passive imputation.

Data were cleaned for episode status, and inpatient (i.e. hospitalised) CV episodes were identified based on classifications used by the Emerging Risk Factors Collaboration.[1] Cardiovascular events were extracted from both primary and secondary positions of ICD-10. Priority was given to CV events reported in the primary position (n = 70). Following this, we selected CV events in the secondary position (n = 116), which had no recording of a CV event in the primary position. Only episodes during the study follow-up were evaluated.

[1] The Emerging Risk Factors Collaboration: analysis of individual data on lipid, inflammatory and other markers in over 1.1 million participants in 104 prospective studies of cardiovascular diseases. *Eur J Epidemiol* **22,** 839–869 (2007). https://doi.org/10.1007/s10654-007-9165-7

Supplementary tables

Table 1 Definitions of diagnoses by ICD-10 coding.

End point	ICD-10 codes
**All cardiovascular disease**	**F01, G46.3-G46.7, G458, G459 I11.0, I13.0, I13.2, I20.0-I20.1, I20.8-I25, I50, I60, I61, I.62, I63, I64, I65-I69, I70.2, I71.3-I71.9, I72, I73.9-I79 + cardiac death**
**Diseases of the arteries**	**I70.2, I72, I73.9-I79**
Peripheral arterial disease	I70.2, I73.9
Diseases of arteries, arterioles and capillaries	I72, I74-I79
**Coronary heart disease**	**I20.0-I20.1, I20.8-I25**
Angina	I20.1, I20.8-I20.9
Unstable angina	I20.0, I24
Coronary heart disease not otherwise specified	I25
Acute myocardial infarction (MI), and certain current complications following acute MI	I21, I23
Subsequent myocardial infarction	I22
**All stroke**	**I60, I61, I.62, I63, I64, I65-I69, F01, G46.3-G46.7, G458, G459**
Subarachnoid haemorrhage	I60
Intra-cerebral haemorrhage	I61
Cerebral infarction	I63
Stroke, not specified as haemorrhage or infarction	I64
Stroke syndromes	G46.3-G46.7
Transient ischemic attack	G458, G459
Other stroke	I62, I65-I69, F01
**Heart failure**	**I11.0, I13.0, I13.2, I50**
Heart failure	I50
Hypertensive heart disease with (congestive) heart failure	I11.0
Hypertensive heart and renal disease with (congestive) heart failure	I13.0
Hypertensive heart and renal disease with both (congestive) heart failure and renal disease	I13.2
**Cardiac death**	**Adjudicated**
**Other vascular deaths**	**I71.3-I71.9**
Abdominal aortic aneurysm	I71.3-I71.9

Table 2 Fatal or non-fatal hospitalised cardiovascular disease by ICD-10 coding position.

	All cardiovascular disease			AECOPD	
	**Primary ICD-10 position**	**Secondary ICD-10 position**	**Cardiac death**	**Primary ICD-10 position**	**Secondary ICD-10 position**
1	I714			J441	
2	I209			J441	
3	I501				
4	I771			J440	
5	I251				
6	I251				
7	I219				J440
8	I251				
9	I745				
10	I259				
11	I251			J441	
12	I251				
13	I500			J440	
14	I743			J441	
15	I64X			J440	
16	I714				
17	I219			J440	
18	I211				
19	I64X				
20	I739				
21	I739				
22	I639				
23	I251			J441	
24	I652				
25	I251			J440	
26	I251				
27	I632				
28	I251			J440	
29	I660			J440	
30	I500			J441	
31	I771				
32	I251			J441	
33	I638			J441	
34	I702			J440	
35	I200			J441	
36	I652				
37	I501			J441	
38	I214				
39	I64X			J440	
40	I509				
41	I739				
42	I739				
43	I639				J440
44	I743				
45	I251				
46	I251			J440	
47	I219				
48	I638				
49	I251			J440	
50	I251			J440	
51	I639				J440

52	I500				
53	I251				
54	I251			J441	
55	I639				
56	I652				J440
57	I219				
58	I635				
59	I214			J440	
60	I251				
61	I739				J440
62	I639				
63	I251			J440	
64	I200				J440
65	I214				J440
66	I251				
67	I634				J440
68	I209				J440
69	I714				
70	I509				
71		I209		J440	
72		I501			
73		I713			J440
74		I714			
75		I252			J440
76		I259		J440	
77		I252		J440	
78		I500		J441	
79		I694			
80		I209			
81		I209			
82		I259		J441	
83		I509		J440	
84		I500			J440
85		I509			J440
86		I200			
87		I219		J440	
88		I714			J440
89		I780			J440
90		I219		J440	
91		I252		J440	
92		I714		J441	
93		I209			J440
94		I251		J440	
95		I259			J440
96		I501		J440	
97		I252			
98		I252			
99		I500			J440
100		I252			J440
101		I252			
102		I652			
103		I500		J440	
104		I259		J441	
105		I251			
106		I501		J440	
107		I252			
108		I255			J440
109		I259			J440

Fermont JM, et al. BMJ Open 2021; 10:e038360. doi: 10.1136/bmjopen-2020-038360

110		I500		J440	
111		I252			
112		I252		J440	
113		I259			
114		I678		J441	
115		I501			J440
116		I678		J440	
117		I501			J440
118		I678		J440	
119		I259			
120		I209			
121		I209		J441	
122		I500		J441	
123		I259			J440
124		I259			J440
125		I259			J440
126		I209		J440	
127		I252			J440
128		I509		J440	
129		I209			
130		I259			
131		I259			
132		I259		J440	
133		I209			
134		I679			
135		I678		J440	
136		I500		J440	
137		I501			
138		I500		J441	
139		I209			
140		I652		J440	
141		I209			
142		I678			
143		I209			
144		I209		J441	
145		I501		J441	
146		I209		J440	
147		I209		J440	
148		I739			
149		I509			
150		I678		J441	
151		I252		J440	
152		I252			
153		I501		J440	
154		I678			
155		I251		J440	
156		I252			J440
157		I500		J440	
158		I252		J440	
159		I252		J440	
160		I634			
161		I679			
162		I252		J440	
163		I252			
164		I500			J440
165		I209		J441	
166		I739			J440
167		I739		J440	

168		I259			
169		I509			J440
170		I252			
171		I209			J440
172		I798		J440	
173		I500			
174		I209			
175		I501		J440	
176		I219		J441	
177		I209			
178		I252		J440	
179		I252			
180		I671		J441	
181		I259			
182		I259		J440	
183		I671			
184		I259			J440
185		I251			J440
186		I714			J440
187			Cardiac		
188			Cardiac		
189			Cardiac		
190			Cardiac		
191			Cardiac		
192			Cardiac		
Total recorded	70	116	6	74	35

Twenty-five individuals with a CV event recorded in the primary position also had an acute exacerbation of COPD (AECOPD) recorded in the primary position during the study period. In addition, 49 individuals with a CV event recorded in the secondary position also had an AECOPD recorded in the primary position during the study period.

Table 3 Conventional cardiovascular disease risk factors at baseline, their hazard ratios for cardiovascular disease.

	Median (IQR) or No. (%)	HR (95% CI)[a]	P value	HR (95% CI)[b]	P value
**Conventional CVD risk factors**					
Age – per 10 year increase	67 (62-73)	1.58 (1.30 to 1.92)	< 0.001	1.59 (1.29 to 1.99)	< 0.001
Sex – males	434 (61)	1.28 (0.94 to 1.74)	0.113	1.16 (0.84 to 1.60)	0.375
Smoking – current	218 (31)	0.95 (0.68 to 1.31)	0.744	0.92 (0.66 to 1.29)	0.632
HDL – per 1 mmol/L increase	1.4 (1.2-1.7)	0.73 (0.52 to 1.03)	0.074	0.86 (0.60 to 1.23)	0.402
Total cholesterol – per 1 mmol/L increase	5.0 (4.3-5.8)	0.80 (0.70 to 0.92)	0.002	0.89 (0.76 to 1.03)	0.118
SBP – per 10 mmHg increase	142 (131-154)	0.91 (0.84 to 0.99)	0.023	0.90 (0.82 to 0.98)	0.016
Diabetes – yes	82 (12)	1.50 (1.01 to 2.22)	0.044	1.15 (0.76 to 1.75)	0.511
Hypertension treatment – yes	245 (34)	1.79 (1.34 to 2.38)	< 0.001	1.76 (1.31 to 2.36)	< 0.001

Values are given as the median and interquartile range (IQR), or No. of cases (%). Baseline data of 714 patients are included. All models are stratified by recruitment site.
[a]Model includes age and sex.
[b]Model includes conventional cardiovascular disease risk factors: age, sex, smoking, high-density lipoprotein, total cholesterol, systolic blood pressure, diabetes, hypertension drug treatment.
CI = confidence interval. HDL = high-density lipoprotein. SBP = systolic blood pressure. CV = cardiovascular.

Fermont JM, *et al. BMJ Open* 2021; 10:e038360. doi: 10.1136/bmjopen-2020-038360

Table 4 Arterial stiffness at baseline, their hazard ratios for cardiovascular disease.

	**Median (IQR) or No. (%)**	**HR (95% CI)[a]**	**P value**	**HR (95% CI)[b]**	**P value**
**Measures of arterial stiffness**					
PWV – per 1 m/sec increase	9.8 (8.4-11.8)	1.01 (0.95 to 1.07)	0.796	0.99 (0.93 to 1.06)	0.749
CIMT – per 1 mm increase	0.81 (0.71-0.96)	1.09 (0.49 to 2.45)	0.827	1.08 (0.48 to 2.43)	0.861
AIx – per 5% increase	28 (20-34)	0.85 (0.77 to 0.93)	< 0.001	0.90 (0.82 to 0.99)	0.027

Values are given as the median and interquartile range (IQR), or No. of cases (%). Baseline data of 714 patients are included. All models are stratified by recruitment site.
[a]Model includes age and sex.
[b]Model includes conventional cardiovascular disease risk factors: age, sex, smoking, high-density lipoprotein, total cholesterol, systolic blood pressure, diabetes, hypertension drug treatment. Carotid intima-media thickness further included systolic blood pressure. Carotid-femoral aortic pulse wave velocity further included mean arterial pressure and resting heart rate. AIx further included resting heart rate and height.
CI = confidence interval. PWV = pulse wave velocity. CIMT = carotid intima-media thickness. AIx = augmentation index.

Fermont JM, *et al. BMJ Open* 2021; 10:e038360. doi: 10.1136/bmjopen-2020-038360

Table 5 Alternative measures at baseline, their hazard ratios for cardiovascular disease.

	**Median (IQR) or No. (%)**	**HR (95% CI)[a]**	**P value**	**HR (95% CI)[b]**	**P value**
**Other risk factors**					
CRP – per twofold increase	1.21 (0.47 to 2.01)	1.22 (1.07 to 1.39)	0.002	1.20 (1.06 to 1.37)	0.005
Fibrinogen – per twofold increase	1.22 (1.06 to 1.36)	2.27 (1.20 to 4.31)	0.012	2.12 (1.10 to 4.07)	0.024
Glucose – per twofold increase	1.59 (1.50 to 1.69)	1.18 (0.46 to 2.99)	0.730	0.83 (0.32 to 2.18)	0.709
BMI – per 1 kg/m^2 increase	27 (23-31)	1.04 (1.01 to 1.06)	0.002	1.02 (0.99 to 1.05)	0.089
GOLD – per 1 stage increase	1 (1-2)	1.22 (0.98 to 1.52)	0.071	1.29 (1.03 to 1.61)	0.026
4MGST – per 1 second increase	4.2 (3.5 to 5.2)	1.09 (1.03 to 1.16)	0.002	1.07 (1.01 to 1.14)	0.020
6MWT distance – per 30 metre increase	366 (255 to 440)	0.91 (0.88 to 0.94)	< 0.001	0.91 (0.88 to 0.95)	< 0.001
BODE – per 1 point increase	3 (1-5)	1.13 (1.07 to 1.20)	< 0.001	1.15 (1.08 to 1.21)	< 0.001

Values are given as the median and interquartile range (IQR), or No. of cases (%). Baseline data of 714 patients are included. All models are stratified by recruitment site.
[a]Model includes age and sex.
[b]Model includes conventional cardiovascular disease risk factors: age, sex, smoking, high-density lipoprotein, total cholesterol, systolic blood pressure, diabetes, hypertension drug treatment.
CI = confidence interval. CRP = C-reactive protein. BMI = body mass index. GOLD = global initiative for chronic obstructive lung disease. 4MGS = four-metre gait speed. 6MWT = six-minute walk test. BODE = body mass index, obstruction, dyspnoea, exercise.

Fermont JM, *et al. BMJ Open* 2021; 10:e038360. doi: 10.1136/bmjopen-2020-038360

Supplementary figures

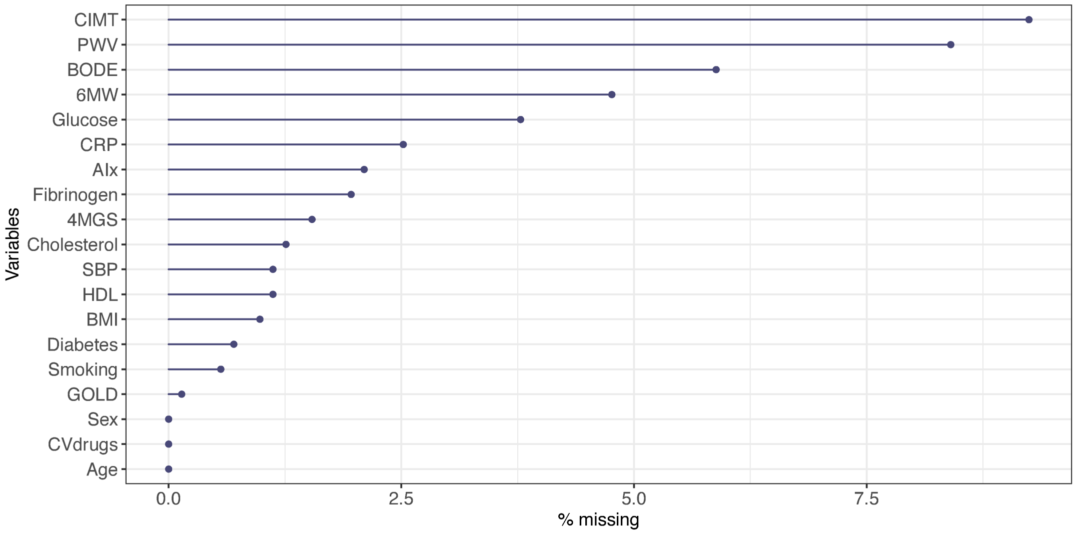

Figure 1 Percentage of missing values.
CIMT = carotid intima-media thickness. PWV = pulse wave velocity. BODE = body mass index, obstruction (i.e. forced expiratory volume in one second), dyspnoea score, exercise (i.e. six-minute walk test distance). 6MWT = six-minute walk test. CRP = C-reactive protein. AIx = augmentation index. 4MGS = four-metre gait speed. SBP = systolic blood pressure. HDL = high-density lipoprotein. BMI = body mass index. GOLD = global initiative for obstructive lung disease. CV = cardiovascular.

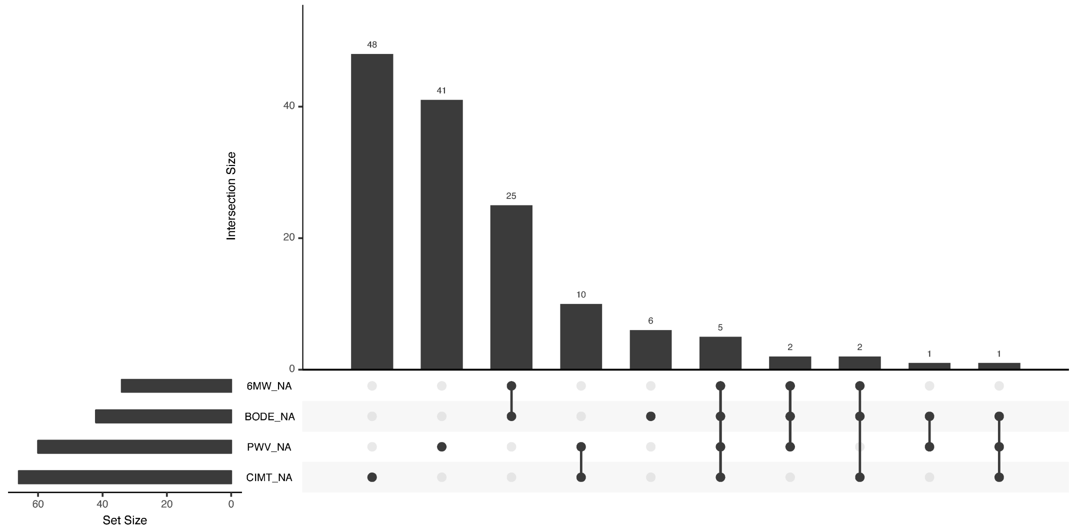

Figure 2 Percentage and pattern of missing values in key baseline characteristics.
6MWT, six-minute walk test. BODE = body mass index, obstruction (i.e. forced expiratory volume in one second), dyspnoea score, exercise (i.e. six-minute walk test distance). PWV, pulse wave velocity. CIMT, carotid-intima media thickness.

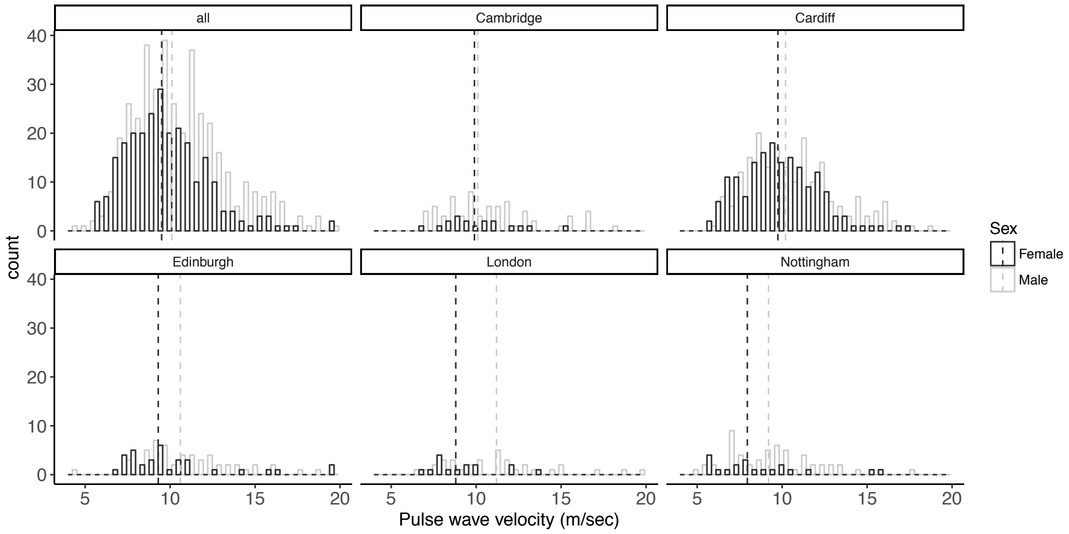

Figure 3 Differences in baseline pulse wave velocity.
Histograms displaying the distribution of PWV by sex and recruitment site. Dashed lines indicate median values by sex. Where only one median line is visible, medians for both sexes are similar.

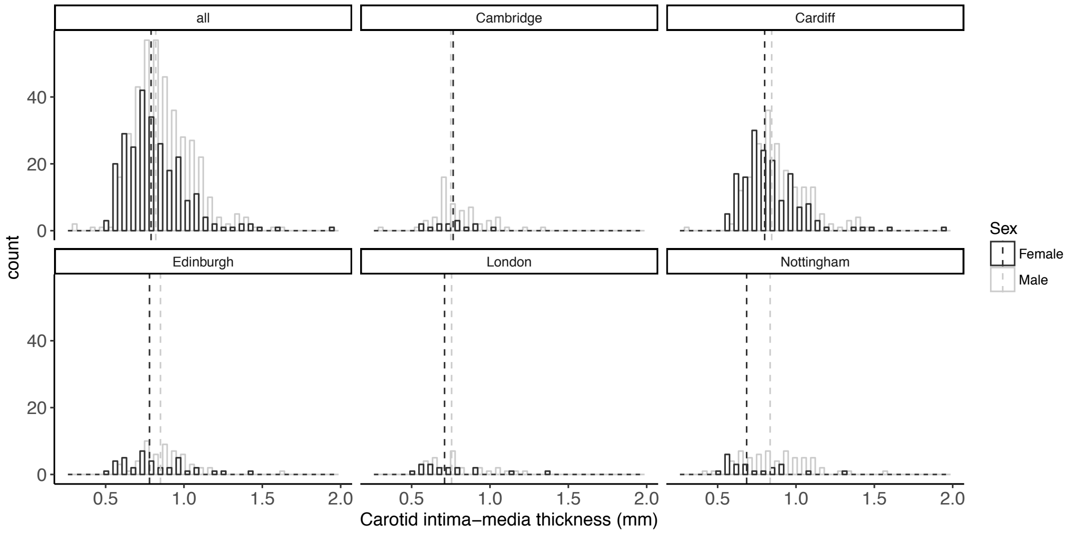

Figure 4 Differences in baseline carotid intima-media thickness.
Histograms displaying the distribution of CIMT by sex and recruitment site. Dashed lines indicate median values by sex. Where only one median line is visible, medians for both sexes are similar.

Fermont JM, *et al. BMJ Open* 2021; 10:e038360. doi: 10.1136/bmjopen-2020-038360

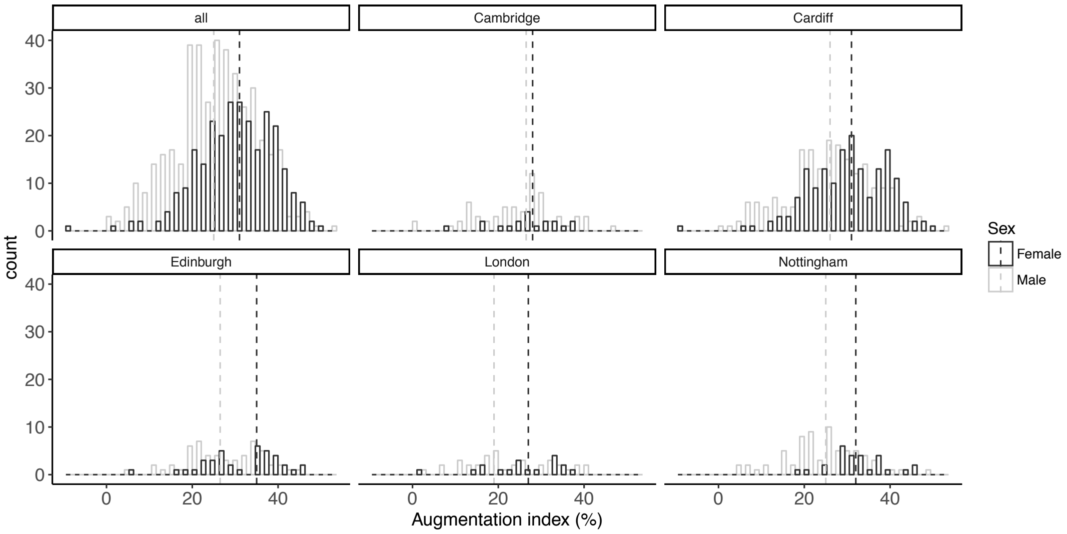

Figure 5 Differences in baseline augmentation index.
Histograms displaying the distribution of AIx by sex and recruitment site. Dashed lines indicate median values by sex.

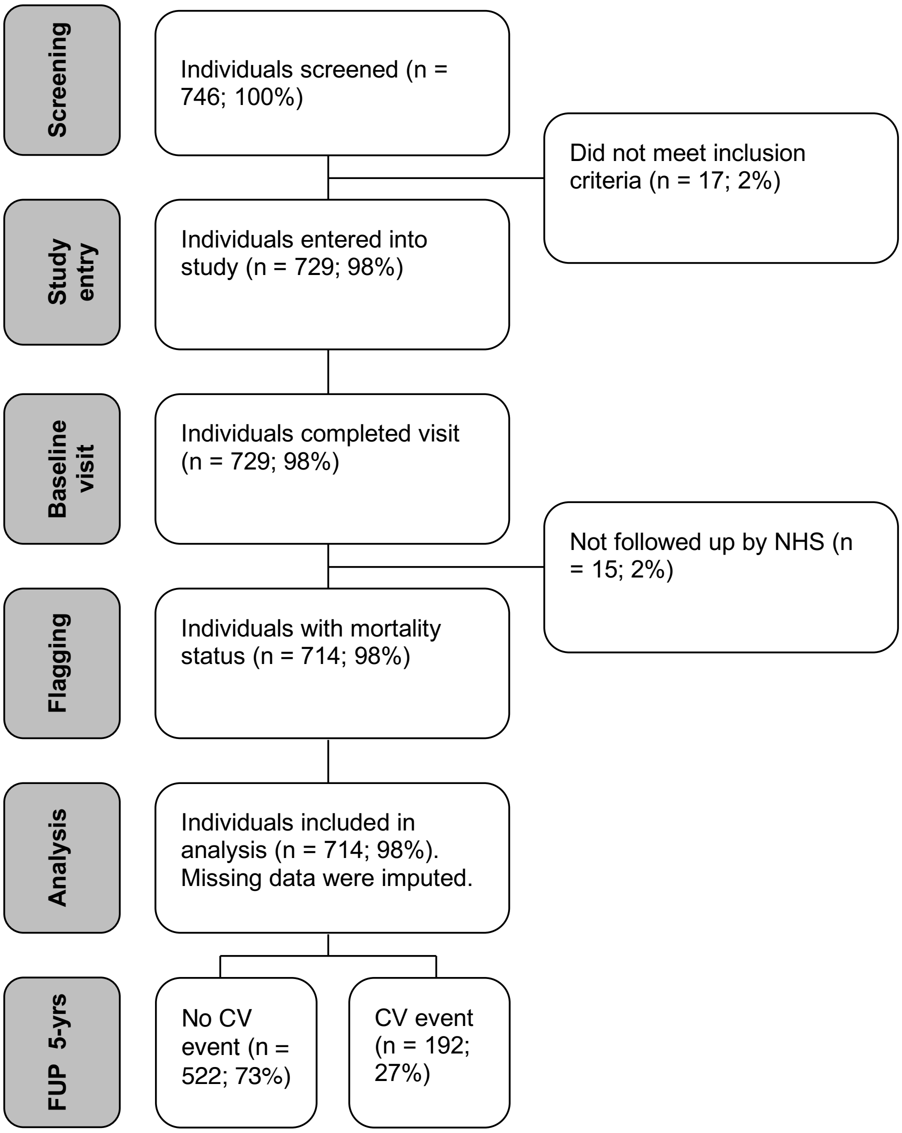

Figure 6 Participant enrolment flow diagram with five years follow up.

Total number of CV events (n = 192): peripheral arterial disease (n = 9), diseases of arteries, arterioles and capillaries (n = 7), angina (n = 21), unstable angina (n = 3), coronary heart disease not otherwise specified (n = 63), acute myocardial infarction (MI), and certain current complications following acute MI (n = 11), cerebral infarction (n = 11), stroke, not specified as haemorrhage or infarction (n = 3), other stroke (n = 18), heart failure (n = 32), abdominal aortic aneurysm (n = 8), and cardiac death (n = 6). FEV_1 = forced expiratory volume one second. FVC = forced vital capacity. NHS = National Health Services. FUP = follow-up period.
