# Supplementary material for: Cardiovascular risk prediction using physical performance measures in COPD: results from a multicentre observational study
Source: BMJ Open. 2020 Dec 28;10(12):e038360. doi: 10.1136/bmjopen-2020-038360 (PMC7772292; doi:10.1136/bmjopen-2020-038360)
Supplement: Author's manuscript [file bmjopen-2020-038360.draft_revisions.pdf]

BMJ Open

BMJ Open is committed to open peer review. As part of this commitment we make the peer review history of every article we publish publicly available.

When an article is published we post the peer reviewers' comments and the authors' responses online. We also post the versions of the paper that were used during peer review. These are the versions that the peer review comments apply to.

The versions of the paper that follow are the versions that were submitted during the peer review process. They are not the versions of record or the final published versions. They should not be cited or distributed as the published version of this manuscript.

BMJ Open is an open access journal and the full, final, typeset and author-corrected version of record of the manuscript is available on our site with no access controls, subscription charges or pay-per-view fees (http://bmjopen.bmj.com).

If you have any questions on BMJ Open's open peer review process please email info.bmjopen@bmj.com

BMJ Open

Physical performance measures improves cardiovascular risk prediction in COPD: a multi-centre prospective UK study.

Journal:	*BMJ Open*
Manuscript ID	bmjopen-2020-038360
Article Type:	Original research
Date Submitted by the Author:	07-Mar-2020
Complete List of Authors:	Fermont, Jilles; University of Cambridge, Department of Medicine<br>Fisk, Marie; University of Cambridge<br>Bolton, Charlotte; University of Nottingham<br>MacNee, William; The University of Edinburgh<br>Cockcroft, John; Columbia University Medical Center<br>Fuld, Jonathan; Cambridge University Hospitals NHS Foundation Trust, Centre for Self Management Support<br>Cheriyan, Joseph; University of Cambridge Medicine, Division of Experimental Medicine & Immunotherapeutics<br>Mohan, Divya; GSK R&D<br>Mäki-Petäjä, Kaisa ; University of Cambridge<br>Al-Hadithi, Ali ; University of Cambridge<br>Tal-Singer, Ruth; GSK R&D<br>Müllerova, Hana; GSK R&D<br>Polkey, Mike; Royal Brompton Hospital<br>Wood, Angela; University of Cambridge<br>McEniery, Carmel M.; University of Cambridge<br>Wilkinson, Ian; University of Cambridge; Cambridge Clinical Trials Unit,
Keywords:	Chronic airways disease < THORACIC MEDICINE, EPIDEMIOLOGY, RESPIRATORY MEDICINE (see Thoracic Medicine), Cardiac Epidemiology < CARDIOLOGY, PRIMARY CARE

SCHOLARONE™
Manuscripts

I, the Submitting Author has the right to grant and does grant on behalf of all authors of the Work (as defined in the below author licence), an exclusive licence and/or a non-exclusive licence for contributions from authors who are: i) UK Crown employees; ii) where BMJ has agreed a CC-BY licence shall apply, and/or iii) in accordance with the terms applicable for US Federal Government officers or employees acting as part of their official duties; on a worldwide, perpetual, irrevocable, royalty-free basis to BMJ Publishing Group Ltd ("BMJ") its licensees and where the relevant Journal is co-owned by BMJ to the co-owners of the Journal, to publish the Work in this journal and any other BMJ products and to exploit all rights, as set out in our licence.

The Submitting Author accepts and understands that any supply made under these terms is made by BMJ to the Submitting Author unless you are acting as an employee on behalf of your employer or a postgraduate student of an affiliated institution which is paying any applicable article publishing charge ("APC") for Open Access articles. Where the Submitting Author wishes to make the Work available on an Open Access basis (and intends to pay the relevant APC), the terms of reuse of such Open Access shall be governed by a Creative Commons licence – details of these licences and which Creative Commons licence will apply to this Work are set out in our licence referred to above.

Other than as permitted in any relevant BMJ Author's Self Archiving Policies, I confirm this Work has not been accepted for publication elsewhere, is not being considered for publication elsewhere and does not duplicate material already published. I confirm all authors consent to publication of this Work and authorise the granting of this licence.

For peer review only

Physical performance measures improves cardiovascular risk prediction in COPD: a multi-centre prospective UK study.

Jilles M. Fermont[1,2$], Marie Fisk[1], Charlotte E. Bolton[3], William Macnee[4], John Cockcroft[5], Jonathan Fuld[6], Joseph Cheriyan[1], Divya Mohan[7], Kaisa M. Mäki-Petäjä[1], Ali B.A.K. Al-Hadithi[1], Ruth Tal-Singer[7], Hana Müllerova[8], Michael I. Polkey[9], Angela M. Wood[2,10-13], Carmel McEniery[1], Ian B. Wilkinson[1,14], on behalf of the ERICA consortium.

[1]Division of Experimental Medicine and Immunotherapeutics, Department of Medicine, University of Cambridge, Cambridge, United Kingdom;

[2]British Heart Foundation Cardiovascular Epidemiology Unit, Department of Public Health and Primary Care, University of Cambridge, Cambridge, United Kingdom;

[3]Division of Respiratory Medicine and NIHR Nottingham BRC respiratory theme, University of Nottingham, Nottingham, United Kingdom;

[4]Centre for Inflammation Research, Queen's Medical Research Institute, University of Edinburgh, Edinburgh, Scotland United Kingdom;

[5]Department of Cardiology, Columbia University Medical Centre, New York, United States;

[6]Department of Respiratory Medicine, Cambridge University Hospitals NHS Foundation Trust, Cambridge, United Kingdom;

[7]Medical Innovation, Value Evidence Outcomes, GSK R&D, PA, United States;

[8]GSK R&D, Uxbridge, United Kingdom;

[9]Department of Respiratory Medicine, Royal Brompton Hospital, London, United Kingdom;

[10]British Heart Foundation Centre of Research Excellence, University of Cambridge, Cambridge, United Kingdom;

[11]National Institute for Health Research Blood and Transplant Research Unit in Donor Health and Genomics, University of Cambridge, Cambridge, United Kingdom;

[12]National Institute for Health Research Cambridge Biomedical Research Centre, University of Cambridge and Cambridge University Hospitals, Cambridge, United Kingdom;

[13]Health Data Research UK Cambridge, Wellcome Genome Campus and University of Cambridge, Cambridge, United Kingdom;

[14]Cambridge Clinical Trials Unit, Cambridge University Hospitals NHS Foundation Trust, Addenbrooke's Hospital, Cambridge, United Kingdom.

For peer review only

$Corresponding author: Jilles M. Fermont, Division of Experimental Medicine and Immunotherapeutics, Department of Medicine, University of Cambridge, Cambridge CB1 8RN, United Kingdom. Tel: +44 (0)12 2333 6806. E-mail: jmf88@medschl.cam.ac.uk.

Word count: 3022

Keywords: Chronic Obstructive Pulmonary Disease; COPD; Biomarkers; Prognosis; Cardiovascular Diseases.

For peer review only

ABSTRACT

Objectives Although cardiovascular disease (CVD) is a common co-morbidity associated with chronic obstructive pulmonary disease (COPD), it is unknown how to improve prediction of cardiovascular (CV) risk in COPD individuals. Traditional CV risk scores have been tested in different populations but not uniquely in COPD. The potential of alternative markers to improve CV risk prediction in COPD individuals is unknown. We aimed to determine the predictive value of the Framingham general CV risk score in COPD, and to determine if additional markers improve prediction beyond conventional factors.

Design Data from the ERICA cohort, which enrolled 729 GOLD stage II-IV COPD individuals were used. Linked hospital episode statistics and survival data were prospectively collected for a median 4.5 years follow-up.

Setting Five UK centres with an interest in COPD.

Participants Population-based sample including 714 individuals with spirometry-defined COPD, at least ten pack years and who were clinically stable for more than four weeks.

Interventions Baseline measurements included aortic pulse wave velocity (aPWV), carotid intima media thickness (CIMT), C-reactive protein (CRP), fibrinogen, spirometry and BODE-Index, six-minute walk test (6MWT) and four-metre gait speed (4MGS) test.

Primary and secondary outcome measures New occurrence (first event) of fatal or non-fatal hospitalised CVD.

Results Out of 714 participants, 232 (32%) had CV-hospitalisation and five died due to CVD. The overall Framingham model C-statistic was 0.696 (95% CI 0.691 to 0.701). aPWV and CIMT had no association with study outcome; nor improved model prediction. CRP, fibrinogen, BODE Index, 4MGS and 6MWT were associated with the outcome; independently of conventional risk factors ($p < 0.05$ for all). However, only 6MWT improved model discrimination (C = 0.725 [0.720 to 0.731]).

For peer review only

Conclusions Poor physical performance defined by the 6MWT improves prediction of CV hospitalisation in COPD individuals.

Trial registration UK Clinical Trials Gateway.

For peer review only

ARTICLE SUMMARY

Strengths and limitations of this study

- This is the first study assessing the utility of the Framingham model for cardiovascular disease risk prediction and the value of additional markers of risk within a COPD cohort.
- Patient level cohort data were linked to hospital admission data (i.e. hospital episode statistics) obtained from the National Health Services in England, Scotland and Wales, and Office for National Statistics record of mortality with analyses limited to five years of follow-up.
- Hospitalised cardiovascular episodes were coded based on ICD-10 classifications extracted from both primary and secondary positions.
- A robust multivariable prediction model, with 10-fold cross validation and 200 replications, was used to evaluate a wide range of cardiovascular and physical performance biomarkers.
- Generalisability of our results is limited to those with mainly moderate COPD in the UK with NHS hospitalisations.

For peer review only

INTRODUCTION

Chronic obstructive pulmonary disease (COPD) is associated with a two to three-fold increased risk of coronary artery disease and other cardiovascular (CV) comorbidities.[1,2] COPD and cardiovascular disease (CVD) are common conditions that share important common risk factors, such as smoking and physical inactivity, and both tend to affect older people. In addition, both conditions are often associated with raised systemic inflammatory markers and increased arterial stiffness.[3,4]

Given the global healthcare burden associated with CVD,[5] there is incentive to improve the accuracy of CV risk prediction in different populations. Individuals with COPD may be considered constitutively at high CV risk given their age and smoking history. However, it is unknown whether classic CV risk prediction models such as the Framingham general CV risk score, which predicts an individual's ten year risk of developing CVD based on an algorithm of weighted risk factors and has been tested in a number of different populations,[6–9] performs well in individuals with COPD.[10,11]

Conceptually, the performance of the Framingham model might be improved by additional measures. Candidate measurements include surrogate CV risk markers that impart mechanistic information (i.e. aortic pulse wave velocity (aPWV) or carotid intima-media thickness (CIMT)), inflammatory markers (i.e. C-reactive protein (CRP)) or measures of physical performance enhance CV risk prediction in COPD individuals. In fact, the value of these measures in CV risk prediction have been explored in different population groups. For example, the six-minute walk test (6MWT) significantly improved risk prediction in patients with stable coronary disease.[12] In intermediate CV risk individuals without CVD, adding CRP or fibrinogen to conventional risk factors, modestly improved CV event prediction.[13] A meta-analysis of aPWV studied in different disease cohorts, showed it improved CV event prediction independently of conventional risk factors.[14] In contrast, CIMT measurement, although associated with CV risk factors, did not significantly improve risk prediction beyond traditional factors in individuals with hypertension.[15] Given that individuals with COPD, in addition to having high CV risk based on conventional factors, also have increased aPWV and CIMT[4] increased inflammatory markers,[16] reduced lung function that is associated with increased CV risk in the general population,[17] and reduced physical performance,[18,19] the clinical significance of these findings in relation to CV risk prediction is an important question to address.

For peer review only - http://bmjopen.bmj.com/site/about/guidelines.xhtml

The aims of our study were to firstly determine the predictive value of classical Framingham risk factors (FRF) for CV risk, defined by new occurrence (first event) of fatal or non-fatal hospitalised CVD in individuals with stable global initiative for chronic obstructive lung disease (GOLD) stage II-IV[20] COPD. The secondary aim was to determine whether addition of alternative CV measures (i.e. aPWV and CIMT), inflammatory markers (i.e. CRP and fibrinogen), COPD severity (i.e. GOLD stage and BODE Index), as well as physical performance tests commonly used in COPD research studies (i.e. 6MWT and four-metre gait speed (4MGS)) improved the predictive value of the FRF for CV risk prediction. We addressed these questions in the Evaluation of the Role of Inflammation in Chronic Airways disease (ERICA) COPD cohort using study data and linked UK electronic health records.

For peer review only - http://bmjopen.bmj.com/site/about/guidelines.xhtml

METHODS

Study design and participants

The ERICA study is a multi-centre observational, cohort study with 729 individuals with stable GOLD stage II-IV[20] COPD, established to identify important CV and physical performance biomarkers that could be targeted to improve the outcomes of COPD individuals. Full details of the protocol have been provided elsewhere.[21] Baseline data captured included demographics, spirometry, blood circulating biochemical markers, measures of arterial stiffness (i.e. aPWV and augmentation index (AIx)), CIMT and physical performance (i.e. 4MGS and 6MWT). The longitudinal component of the study included follow-up of ERICA participants linked for UK National Health Services (NHS) electronic healthcare records (i.e. hospital episode statistics) and Office for National Statistics (ONS) record of mortality.

Clinical measures

After four hours of fasting, with no bronchodilators for six hours, and ten minutes of supine rest, carotid-femoral aPWV and AIx measurements were taken using a SphygmoCor system as previously described.[22] Carotid intima-media thickness of the common carotid arteries was measured using B-mode ultrasound at a distance of one cm from the carotid bulb with a 7–12 MHz linear probe.[23] The thickest artery of the two was included in the analysis. Systemic inflammatory markers of plasma fibrinogen and serum CRP were obtained after fasting. Physical performance measures 6MWT[24] and 4MGS[25] were assessed according to guidelines. Diabetes status and anti-hypertensive treatment were self-reported at baseline study visit. Disease severity was defined according to the GOLD classification.[20] Points for the BODE (Body mass index (BMI), airflow Obstruction, Dyspnoea, and Exercise capacity) Index were assigned as described by Celli *et al*.[26]

Cardiovascular hospitalisation and mortality

Electronic healthcare records (i.e. admitted patient care data) were obtained from NHS Digital, NHS Scotland, and NHS Wales and linked to baseline study variables. Hospital data included admissions for non-fatal CV episodes extracted from both primary and secondary international statistical classification of diseases and related health problems 10th revision coding (ICD-10) positions, and included diseases of the arteries, all stroke, and heart failure (online supplementary table 1). Atrial

For peer review only

fibrillation and flutter, and hypertensive diseases were excluded. Cause of death reported on death certificates were adjudicated by CV and pulmonary physicians.

Outcomes and predictors of interest

The primary outcome measure was defined as new occurrence (first event) of fatal or non-fatal hospitalised CVD. Time to primary outcome was calculated from the difference between the baseline visit date and either the date of death or first hospitalised CV attendance up to November 2017, when follow-up discontinued. The association between FRF (age, sex, smoking status [current/non-smoker], HDL cholesterol, total cholesterol, systolic blood pressure (SBP), diabetes [yes/no] and treatment for high blood pressure [yes/no]) and study outcome measure were evaluated. In addition, we evaluated whether BMI, aPWV and CIMT, bloods (fasting glucose), blood inflammatory biomarkers (i.e. CRP and fibrinogen), COPD severity (i.e. GOLD stage and BODE Index), and measures of physical performance (i.e. 6MWT and 4MGS), added predictive value to the Framingham model.

A supplementary outcome of interest was to report all-cause and cause-specific mortality (defined as CV, pulmonary, cancer, or other) stratified by sex, captured over the longitudinal period of follow-up analysed in the study.

Statistical analysis

Hazard ratios with 95% confidence intervals (CI) were estimated using Cox regression. Carotid-femoral aortic pulse wave velocity, CIMT, inflammatory biomarkers and physical performance tests, added to the conventional Framingham model, were adjusted for age and sex and stratified by study centre. Carotid-femoral aortic pulse wave velocity was also adjusted for mean arterial pressure and heart rate. Discrimination (i.e. Harrell's C-statistic)[27,28] was assessed using 10-fold cross validation with 200 replications.[29] Hazard ratios for log-transformed biomarkers represent a twofold increase in the biomarker. See online supplementary text 1 for further statistical analyses details.

Observational data is reported according to the STROBE statement.[30] All tests were two-sided and statistical significance were defined by 95% CI for hazard ratios not traversing 1 or $p<0.05$. Our analyses were performed using Stata version 13 (College Station. Texas) and R (R Foundation).

For peer review only

RESULTS

Of the 729 individuals included in the study, 714 could be linked with hospital admission and survival records, and were included in the analysis (online supplementary figure 6). The median age was 67 (interquartile range 62-73) years and 434 (61%) individuals were male (table 1). A third (n = 218) of the cohort smoked, 12% (n = 82) had self-reported diabetes, 34% (n = 245) were taking anti-hypertensive medications, and 31% (n = 224) were taking cholesterol-lowering medications at baseline. The median FEV_1 was 1.3 (0.9-1.7) litres (mean ± SD = 1.34 ± 0.53). In total, 232 individuals (32%) had a first event of CV hospitalisation (n = 189 (81%) were in ICD-10 secondary coding position) during median follow-up for 4.5 years and five individuals had CV death without any preceding CV episode. Cardiovascular hospitalisation accounted for the majority (98%) of events analysed. The CV incidence rate was 8.8 (95% CI 7.7 to 10.0) per 100 person-years. See online supplementary table 2 for categorisation of different admission codes for CV hospitalisation.

Table 1 Baseline characteristics (No. = 714).

Characteristic	All individuals (%)
**Framingham risk factors**	
Age (yrs.)	67 (62 to 73)
Male	434 (61)
Current smoker	218 (31)
HDL (mmol/L)	1.4 (1.2 to 1.7)
Cholesterol (mmol/L)	5.0 (4.3 to 5.8)
SBP (mmHg)	142 (131 to 154)
Diabetes mellitus	82 (12)
Drugs to treat hypertension	245 (34)
Cholesterol-lowering therapy	224 (31)
**CV measures**	
aPWV (m/sec)	9.8 (8.4 to 11.8)
CIMT (mm)	0.81 (0.71 to 0.96)
**Alternative measures**	

For peer review only

CRP	1.21 (0.47 to 2.01)
Fibrinogen	1.22 (1.06 to 1.36)
Glucose	1.59 (1.46 to 4.59)
BMI (kg/m^2)	27 (23 to 31)
GOLD (stage)	2 (2 to 3)
4MGS (m/s)	0.95 (0.77 to 1.14)
6MWT distance (metre)	366 (255 to 440)
BODE (point)	3 (1−5)

Values are given as the median and interquartile range (IQR), or No. of cases (%). HDL = high-density lipoprotein. SBP = systolic blood pressure. CV = cardiovascular. aPWV = carotid-femoral aortic pulse wave velocity. CIMT = carotid intima-media thickness. CRP = C-reactive protein. BMI = body mass index. GOLD = global initiative for obstructive lung disease. 4MGS = four-metre gait speed. 6MWT = six-minute walk test. BODE = BMI, obstruction (FEV_1), dyspnoea (Medical Research Council), exercise (6MWT).

Framingham traditional risk factors

Of the FRF, age, diabetes and treatment for high blood pressure had significant positive associations with the study's outcome whereas SBP had a significant negative association and other risk factors (i.e. sex, smoking status, cholesterol and HDL cholesterol) were not significantly associated with CV hospitalisation. Total cholesterol followed by diabetes and use of hypertension drug treatment contributed most to the discriminative ability of the model. The overall discriminative ability of the Framingham model including all FRF had a C-statistic of 0.696, 95% CI 0.691 to 0.701; figure 1 and online supplementary table 3)

PLACE FIGURE 1 HERE

Surrogate cardiovascular risk markers

For peer review only

Neither aPWV nor CIMT were significantly associated with CV hospitalisation, after adjustment for FRF (figure 2 and online supplementary table 4). Moreover, they did not significantly change the discriminative ability of the Framingham model.

PLACE FIGURE 2 HERE

Physical performance measures, COPD severity and inflammatory markers

Multivariable analysis identified that poor physical performance (i.e. reduced 6MWT distance and slower 4MGS) were significantly associated with increased CV hospitalisation, independently of FRF. Severity of COPD defined by higher BODE Index (GOLD stage did not reach statistical significance: HR 1.19 (0.98 to 1.45, p=0.077), as well as inflammatory markers (i.e. CRP and fibrinogen), BMI and fasting glucose also all had significant positive associations with CV hospitalisations independently of FRF.

Predictive modelling indicated significant improvement in risk discrimination when adding BMI (C = 0.705, 95% CI 0.700 to 0.711), BODE (C = 0.711, 95% CI 0.705 to 0.716), 4MGS (C = 0.713, 95% CI 0.707 to 0.718) or 6MWT (C = 0.725, 95% CI 0.720 to 0.731) to the FRF, but not GOLD stage, inflammatory markers or glucose (figure 3). Adding BMI, 4MGS, 6MWT and BODE collectively to the Framingham risk model resulted in a C-statistic of 0.727 (95% CI 0.721 to 0.733), indicating 6MWT primarily accounted for improved discriminative ability of the model.

PLACE FIGURE 3 HERE

All-cause and cause-specific mortality

There were 144 deaths in total (nearly 20% of the cohort), over a median follow-up period of 4.5 years. The majority of deaths were in men (n = 96, 67%) and pulmonary disease was the leading cause in

For peer review only

both sexes (table 2). Pulmonary disease accounted for 65% of deaths in women and 50% in men, followed by cancer (19% in women, 27% in men), CV (6% and 16%), and other (10% and 7% in women and men respectively).

Table 2 Mortality in the ERICA cohort.

**Cause-specific mortality**	Female No. (%)	Male No. (%)	Total No. (%)
Pulmonary	31 (65)	48 (50)	79 (55)
Cardiovascular	3 (6)	15 (16)	18 (13)
Cancer	9 (19)	26 (27)	35 (24)
Other	5 (10)	7 (7)	12 (8)
**All-cause**	48 (100)	96 (100)	144 (100)

Deaths recorded over median follow up of 4.5 years.

For peer review only

DISCUSSION

This is the first study assessing the utility of the Framingham model for CVD risk prediction and the value of additional markers of risk within a COPD cohort. Novel findings include (i) poor physical performance improved the discriminative power of the Framingham model; (ii) BODE Index, BMI, 4MGS and systemic inflammatory markers were positively associated with CV hospitalisations independently of FRF, although they did not improve the model's discriminative ability; (iii) of the FRF; age, diabetes and use of anti-hypertensives were positively associated with study outcome. However, cholesterol, diabetes and use of anti-hypertensives contributed most to the model's prognostic power. SBP had a significant negative association with CV hospitalisations but did not add to the model's predictive ability. Furthermore, aPWV and CIMT, despite providing *in-vivo* mechanistic information about the arterial system, had no significant association with CV hospitalisations. Therefore, these data suggest that in COPD, physical performance (assessed by 6MWT) contributes to CV risk estimation defined predominantly by non-fatal CV hospitalisations. Finally, CVD contributed to only a small number of deaths at a median follow-up of 4.5 years. Pulmonary disease, followed by cancer were the major causes of death in both men and women.

We observed a C-statistic of 0.696 based on the conventional Framingham model, which increased to a maximum of 0.727 when adding additional markers; this increment was primarily due to 6MWT. These C-statistic values indicate a moderate predictive ability of the models for the study's outcome. For perspective, a C-statistic of 0.53 was observed in a Framingham model in the very elderly without prior CVD, indicating it does not predict CV mortality in this group.[6] However, in primary care individuals without CVD in the Framingham study, a C-statistic of 0.76 for an outcome of general CVD, demonstrated good discrimination.[10] Since our cohort included individuals with CVD at baseline, and different versions of the Framingham model and CV outcomes are evaluated in various studies, no direct comparison of study results can be made.

Overall, our study emphasises the importance of physical function as a predictor of CV risk. Tests such as 6MWT or 4MGS are proxy measures of overall mobility and physical functioning.[31] Exercise capacity and CV fitness are known to be associated with fatal- and non-fatal CVD,[32] whilst exercise-based cardiac rehabilitation reduces risk of CV events.[33] The 6MWT distance is prognostic in

For peer review only - http://bmjopen.bmj.com/site/about/guidelines.xhtml

patients with stable coronary heart disease,[34] and those with moderate-to-severe heart failure.[35] In our cohort poor physical function improved discriminative ability of the Framingham model beyond conventional factors. This has implications for clinical practice in CV risk assessment for COPD individuals, suggesting it may be helpful to incorporate physical performance into clinical assessment. However, given that 6MWT can be logistically challenging to set up and time-consuming,[36] the faster and simpler 4MGS that also significantly improved the C-statistic could be used as a simpler alternative. In elderly with CVD, 4MGS is comparable to 6MWT in predicting all-cause mortality.[37]

The BODE Index also significantly improved the C-statistic of the CV risk model. However, this was primarily due to the 6MWT component. In fact, although GOLD stage had a positive association with the study outcome, this did not reach statistical significance nor improved prognostic power of the model. Therefore, despite association between airflow limitation and CV risk in general population studies,[17,38] in patients with COPD, physical performance assessment rather than other components of BODE (i.e. spirometry) adds value to CV risk prediction.

Although we did not find an association between inflammatory markers and surrogate markers of CV risk in the baseline cross-sectional component of the ERICA study,[39] both CRP and fibrinogen were associated with CV hospitalisations captured over nearly five years follow-up. That the associations remained significant after adjustment for FRF indicate potential value for identifying high-risk individuals within a COPD population.

The inverse relationship of SBP was an unexpected finding and merits careful consideration. One hypothesis is that SBP has a J-shaped curve for CV events and mortality.[40] Therefore, in this cohort, lower SBP might be a marker of sickness and frailty, hence its association with CV hospitalisations. This negative association of SBP and CV hospitalisations may also explain why no associations between aPWV and CIMT with CV outcomes were found. Moreover, 5-year follow-up is a short time in contrast to CV risk prediction models such as Framingham, which normally predict 10-year risk. Therefore, low SBP may be predictive of CV hospitalisation <5 years given these individuals are likely in poorer health, but over a decade of follow-up, it is unknown whether this negative association persists.[40]

For peer review only

A third of the cohort had a CV related hospitalisation during follow-up and the majority had pre-existing CV comorbidity at baseline. The high numbers of CV hospitalisations, gives perspective of what this comorbidity incurs for patients and the huge healthcare costs involved. In contrast to the sizeable number of CV hospitalisations, was the small number of CV deaths. This may reflect better CV risk management reducing CV mortality. Importantly though, such risk management does not seem to impact CV morbidity, and is an area that requires future research to determine the optimum approach to impact CV morbidity in COPD individuals.

Nearly 20% of the cohort died during follow-up. This is comparable to the TORCH study where approximately 15% of the cohort died over 3-year follow-up.[2] Pulmonary disease, followed by cancer accounted for proportionally more deaths in our cohort compared with TORCH, whereas CV-specific mortality was less (10-15% vs 27% in TORCH). Reasons for these findings are not entirely understood but the sizeable proportion of our cohort already on medicines for dyslipidaemia and blood pressure control may be an important factor. Further studies are needed.

This study has limitations. The Framingham model used was originally designed to predict CV risk defined by development of CVD. However, the majority of our participants already had CVD and were on CV medications, which may be a confounding factor impacting discriminative ability of traditional FRF. Moreover, in our study, CV risk was defined differently (as CV hospitalisation with CV mortality). Hospitalised CV episodes were coded based on ICD-10 classifications[41] extracted from both primary and secondary positions. Although, most CV hospitalisations were recorded in secondary positions, indicating the primary admission might be related to something else. We did not use sex-specific models as originally produced by the Framingham Study given the relatively small cohort size and sex was adjusted for in all analyses. Deprivation scores may be another important determinant of CV risk in COPD individuals worth evaluation in future research, although the relatively small cohort size of our study meant we did not include this in the analysis.

Furthermore, follow-up time for the study was a maximum of five years, whereas Framingham risk score calculates a 10-year risk. Hence, the prognostic value of variables may alter with a different time

For peer review only

For peer review only - http://bmjopen.bmj.com/site/about/guidelines.xhtml

horizon. We did not have access to an independent validation cohort but used cross-validation techniques instead. Generalisability of our results is limited to those with mainly moderate COPD in the UK with NHS hospitalisations. Lastly, we evaluated the Framingham risk model but it is unknown whether other risk algorithms such as QRISK, which is recommended for CV risk prediction according to NICE clinical guidelines[42] may perform better.

CONCLUSION

In a UK COPD cohort, poor physical performance assessed by 6MWT or 4MGS and inflammatory biomarkers are associated with subsequent CV-related hospitalisations, independently of conventional FRF. Importantly, poor physical performance (defined primarily by 6MWT), also significantly improved the predictive discrimination of the Framingham model. These data suggest an assessment of physical performance may enhance CV risk evaluation in individuals with COPD.

ACKNOWLEDGMENTS

The views and opinions expressed are those of the authors and do not necessarily reflect those of the University of Cambridge, the NHS, the NIHR, the Department of Health and Social Care, or other parent institutions of the authors. The corresponding author and IBW had full access to all the data in the study and takes responsibility for the integrity of the data and the accuracy of the data analysis.

Author statement: The following authors made substantial contributions to the conceptualisation or design: JMF, RTS, HM, MIP, AMW and IBW; data curation: JMF, IBW, KMP and AAH; formal analysis: JMF; interpretation: JMF, MF and IBW; funding acquisition: RTS, MIP and IBW; investigation: JMF, MF, CEB, WM, JRC, JF, JC, DM, KMP, AAH, MIP, CMM and IBW; methodology: JMF, AMW, IBW; project administration: JMF, RTS, MIP and IBW; resources: JMF, MF, CEB, WM, JRC, DM, MIP, CMM and IBW; software: JMF; supervision: DM, RTS, HM, MIP, AMW and IBW; validation: JMF; visualisation: JMF; and writing – original draft preparation: JMF. All authors contributed to the reviewing and editing of the manuscript.

Funding: This work was supported by a grant (9157-61188) from Innovate UK (formerly known as Technology Strategy Board) with contributory funding in kind (e.g. scientific expertise and meeting

For peer review only

rooms) from GSK, a consortium partner, who also funded the corresponding author's PhD. As a consortium partner, GSK was involved in the study design, data analysis, decision to publish, and preparation of the manuscript. The specific roles of all authors are articulated in the 'author contributions' section. RTS was a co-investigator on the grant and as a consortium member was involved in the decision to publish, and preparation of the manuscript. IBW, JC and CMM acknowledge funding support from the NIHR Cambridge Comprehensive Biomedical Research Centre. CEB is supported by the NIHR Nottingham BRC respiratory theme. This work was supported by core funding from: the UK Medical Research Council (MR/L003120/1), the British Heart Foundation (RG/13/13/30194; RG/18/13/33946) and the National Institute for Health Research [Cambridge Biomedical Research Centre at the Cambridge University Hospitals NHS Foundation Trust] [*]. This work was also supported by Health Data Research UK, which is funded by the UK Medical Research Council, Engineering and Physical Sciences Research Council, Economic and Social Research Council, Department of Health and Social Care (England), Chief Scientist Office of the Scottish Government Health and Social Care Directorates, Health and Social Care Research and Development Division (Welsh Government), Public Health Agency (Northern Ireland), British Heart Foundation and Wellcome. *The views expressed are those of the authors and not necessarily those of the NHS, the NIHR or the Department of Health and Social Care.

Competing interests: All authors have completed the ICMJE uniform disclosure form at www.icmje.org/coi_disclosure.pdf and declare: GSK, a consortium partner, funded JMFs PhD. HM, RTS and DM were employees of GSK at the time this work was completed and own GSK shares and stock options. JC is employed by Cambridge University Hospitals NHS Foundation Trust and is obligated to spend 50% of his time on GSK clinical trial activity, representing a significant relationship; however, he receives no other benefits or compensation from GSK. MIP received grants from GSK outside the submitted work. IBW received grants from GSK during the conduct of the study and outside the submitted work.

Patient consent for publication: Informed patient consent was obtained in writing from all study participants and permits the publishing of all data included in this article. The investigation conforms

For peer review only

with the principles outlined in the Declaration of Helsinki. Ethical approval was granted by the National Research Ethics Service Committee East of England - Cambridge South (11/EE/0357).

Patient and public involvement statement: Patients and public were not involved in the design, conduct or reporting of this study.

Data availability statement: The dataset underlying these findings, with de-identified participant data (including the data dictionary), are available to interested and qualified researchers upon request and can be obtained from the Cambridge Clinical Trials Unit. Access to hospital episode statistics requires a data sharing agreement with the National Health Services. For data access, please contact cctu@addenbrookes.nhs.uk.

For peer review only

REFERENCES

1 Chen W, Thomas J, Sadatsafavi M, *et al.* Risk of cardiovascular comorbidity in patients with chronic obstructive pulmonary disease: a systematic review and meta-analysis. *Lancet Respir Med* 2015;**3**:631–9. doi:10.1016/S2213-2600(15)00241-6

2 McGarvey LP, John M, Anderson JA, *et al.* Ascertainment of cause-specific mortality in COPD: operations of the TORCH Clinical Endpoint Committee. *Thorax* 2007;**62**:411–5.

3 Garcia-Rio F, Miravitlles M, Soriano JB, *et al.* Systemic inflammation in chronic obstructive pulmonary disease: a population-based study. *Respir Res* 2010;**11**:63. doi:10.1186/1465-9921-11-63

4 Fisk M, McEniery CM, Gale N, *et al.* Surrogate Markers of Cardiovascular Risk and Chronic Obstructive Pulmonary Disease: A Large Case-Controlled Study. *Hypertension* 2018;**71**:499–506. doi:10.1161/HYPERTENSIONAHA.117.10151

5 Lozano R, Naghavi M, Foreman K, *et al.* Global and regional mortality from 235 causes of death for 20 age groups in 1990 and 2010: a systematic analysis for the Global Burden of Disease Study 2010. *Lancet* 2012;**380**:2095–128. doi:10.1016/S0140-6736(12)61728-0

6 de Ruijter W, Westendorp RG, Assendelft WJ, *et al.* Use of Framingham risk score and new biomarkers to predict cardiovascular mortality in older people: population based observational cohort study. *BMJ* 2009;**338**:a3083. doi:10.1136/bmj.a3083

7 Kannel WB, McGee DL. Diabetes and glucose tolerance as risk factors for cardiovascular disease: the Framingham study. *Diabetes Care* 1979;**2**:120—126. doi:10.2337/diacare.2.2.120

8 Wannamethee SG, Shaper AG, Lennon L, *et al.* Metabolic syndrome vs Framingham Risk Score for prediction of coronary heart disease, stroke, and type 2 diabetes mellitus. *Arch Intern Med* 2005;**165**:2644–50. doi:10.1001/archinte.165.22.2644

9 Weiner DE, Tighiouart H, Elsayed EF, *et al.* The framingham predictive instrument in chronic kidney disease. *J Am Coll Cardiol* 2007;**50**:217–24. doi:10.1016/j.jacc.2007.03.037

10 D'Agostino RB, Vasan RS, Pencina MJ, *et al.* General cardiovascular risk profile for use in primary care. *Circulation* 2008;**117**:743–53.

11 Lee HM, Lee J, Lee K, *et al.* Relation Between COPD Severity and Global Cardiovascular Risk in US Adults. *Chest* 2012;**142**:1118–25. doi:10.1378/chest.11-2421

12 Beatty AL, Schiller NB, Whooley MA. Six-minute walk test as a prognostic tool in stable

For peer review only

coronary heart disease: data from the heart and soul study. *Arch Intern Med* 2012;**172**:1096–102. doi:10.1001/archinternmed.2012.2198

13 Collaboration ERF, Kaptoge S, Di Angelantonio E, *et al.* C-reactive protein, fibrinogen, and cardiovascular disease prediction. *N Engl J Med* 2012;**367**:1310–20. doi:10.1056/NEJMoa1107477

14 Ben-Shlomo Y, Spears M, Boustred C, *et al.* Aortic pulse wave velocity improves cardiovascular event prediction: an individual participant meta-analysis of prospective observational data from 17,635 subjects. *J Am Coll Cardiol* 2014;**63**:636–46. doi:10.1016/j.jacc.2013.09.063

15 Bots ML, Groenewegen KA, Anderson TJ, *et al.* Common carotid intima-media thickness measurements do not improve cardiovascular risk prediction in individuals with elevated blood pressure: the USE-IMT collaboration. *Hypertens (Dallas, Tex 1979)* 2014;**63**:1173–81. doi:10.1161/HYPERTENSIONAHA.113.02683

16 Gan WQ, Man SFP, Senthilselvan A, *et al.* Association between chronic obstructive pulmonary disease and systemic inflammation: a systematic review and a meta-analysis. *Thorax* 2004;**59**:574–80. doi:10.1136/thx.2003.019588

17 Hole DJ, Watt GC, Davey-Smith G, *et al.* Impaired lung function and mortality risk in men and women: findings from the Renfrew and Paisley prospective population study. *BMJ* 1996;**313**:711–6. doi:10.1136/bmj.313.7059.711

18 Sciurba F, Criner GJ, Lee SM, *et al.* Six-Minute Walk Distance in Chronic Obstructive Pulmonary Disease. *Am J Respir Crit Care Med* 2003;**167**:1522–7. doi:10.1164/rccm.200203-166OC

19 Camarri B, Eastwood PR, Cecins NM, *et al.* Six minute walk distance in healthy subjects aged 55–75 years. *Respir Med* 2006;**100**:658–65. doi:10.1016/j.rmed.2005.08.003

20 Global Initiative for Chronic Obstructive Lung Disease (GOLD). Global Strategy for the Diagnosis, Management and Prevention of COPD. 2016. http://goldcopd.org/global-strategy-diagnosis-management-prevention-copd-2016/

21 Mohan D, Gale NS, McEniery CM, *et al.* Evaluating the role of inflammation in chronic airways disease: the ERICA study. *COPD* 2014;**11**:552–9. doi:10.3109/15412555.2014.898031

22 Wilkinson IB, Fuchs SA, Jansen IM, *et al.* Reproducibility of pulse wave velocity and

For peer review only

augmentation index measured by pulse wave analysis. *J Hypertens* 1998;**16**:2079–84.https://www.ncbi.nlm.nih.gov/pubmed/9886900

23 Csanyi A, Egervari A. Simple clinical method of average intima-media thickness measurement in the common carotid artery. *Vasa* 1996;**25**:242–8.http://www.ncbi.nlm.nih.gov/pubmed/8795305

24 Laboratories ATSC on PS for CPF. ATS statement: guidelines for the six-minute walk test. *Am J Respir Crit Care Med* 2002;**166**:111–7. doi:10.1164/ajrccm.166.1.at1102

25 Guralnik JM, Simonsick EM, Ferrucci L, *et al.* A short physical performance battery assessing lower extremity function: association with self-reported disability and prediction of mortality and nursing home admission. *J Gerontol* 1994;**49**:M85-94.http://www.ncbi.nlm.nih.gov/pubmed/8126356

26 Celli BR, Cote CG, Marin JM, *et al.* The body-mass index, airflow obstruction, dyspnea, and exercise capacity index in chronic obstructive pulmonary disease. *N Engl J Med* 2004;**350**:1005–12. doi:10.1056/NEJMoa021322

27 Altman DG, Vergouwe Y, Royston P, *et al.* Prognosis and prognostic research: validating a prognostic model. *BMJ* 2009;**338**:b605.

28 Newson RB. Comparing the predictive powers of survival models using Harrell's C or Somers' D. *Stata J* 2010;**10**:339–58.http://www.stata-journal.com/article.html?article=st0198

29 Smith GC, Seaman SR, Wood AM, *et al.* Correcting for optimistic prediction in small data sets. *Am J Epidemiol* 2014;**180**:318–24. doi:10.1093/aje/kwu140

30 von Elm E, Altman DG, Egger M, *et al.* The Strengthening the Reporting of Observational Studies in Epidemiology (STROBE) statement: guidelines for reporting observational studies. *J Clin Epidemiol* 2008;**61**:344–9. doi:10.1016/j.jclinepi.2007.11.008

31 Lord SR, Menz HB. Physiologic, psychologic, health predictors of 6-minute walk performance in older people. *Arch Phys Med Rehabil* 2002;**83**:907–11. doi:10.1053/apmr.2002.33227

32 Newman AB, Simonsick EM, Naydeck BL, *et al.* Association of long-distance corridor walk performance with mortality, cardiovascular disease, mobility limitation, and disability. *Jama-Journal Am Med Assoc* 2006;**295**:2018–26. doi:DOI 10.1001/jama.295.17.2018

33 Anderson L, Oldridge N, Thompson DR, *et al.* Exercise-Based Cardiac Rehabilitation for Coronary Heart Disease Cochrane Systematic Review and Meta-Analysis. *J Am Coll Cardiol*

For peer review only

2016;**67**:1–12. doi:10.1016/j.jacc.2015.10.044

34 AL B, Schiller NB FAU - Whooley MA, MA W. Six-minute walk test as a prognostic tool in stable coronary heart disease: data from the heart and soul study. PG - 1096-102 LID - 10.1001/archinternmed.2012.2198 [doi].

35 Opasich C, Pinna GD FAU - Mazza A, Mazza A FAU - Febo O, *et al.* Six-minute walking performance in patients with moderate-to-severe heart failure; is it a useful indicator in clinical practice? PG - 488-96.

36 Holland AE, Spruit MA, Troosters T, *et al.* An official European Respiratory Society/American Thoracic Society technical standard: field walking tests in chronic respiratory disease. *Eur Respir J* 2014;**44**:1428–46. doi:10.1183/09031936.00150314

37 Kamiya K, Hamazaki N, Matsue Y, *et al.* Gait speed has comparable prognostic capability to six-minute walk distance in older patients with cardiovascular disease. *Eur J Prev Cardiol* 2017;**25**:212–9. doi:10.1177/2047487317735715

38 D. SD, Paul MSF. Why Are Patients With Chronic Obstructive Pulmonary Disease at Increased Risk of Cardiovascular Diseases? *Circulation* 2003;**107**:1514–9. doi:10.1161/01.CIR.0000056767.69054.B3

39 Duvoix A, Dickens J, Haq I, *et al.* Blood fibrinogen as a biomarker of chronic obstructive pulmonary disease. *Thorax* 2013;**68**:670–6. doi:10.1136/thoraxjnl-2012-201871

40 Investigators the S, Brook RD, Byrd JB, *et al.* Blood pressure, heart rate, and mortality in chronic obstructive pulmonary disease: the SUMMIT trial. *Eur Heart J* 2018;**39**:3128–34. doi:10.1093/eurheartj/ehy451

41 Emerging Risk Factors Collaboration. The Emerging Risk Factors Collaboration: analysis of individual data on lipid, inflammatory and other markers in over 1.1 million participants in 104 prospective studies of cardiovascular diseases. *Eur J Epidemiol* 2007;**22**:839–69. doi:10.1007/s10654-007-9165-7

42 NICE. Cardiovascular disease: risk assessment and reduction, including lipid modification. 2014. https://www.nice.org.uk/guidance/cg181/chapter/1-Recommendations

For peer review only

Figure legends

Figure 1. Framingham risk factors at baseline, their hazard ratios and discriminative ability for cardiovascular disease.

Values are given as the median and interquartile range (IQR), or No. of cases (%). Baseline data of 714 patients are included. All models are stratified by recruitment site.

[a]Adjusted for age and sex.

[b]Adjusted for Framingham risk factors: age, sex, smoking, high-density lipoprotein, total cholesterol, systolic blood pressure, diabetes, hypertension medication.

CI = confidence interval. HDL = high-density lipoprotein. SBP = systolic blood pressure. CV = cardiovascular.

There were < 5% missing values for descriptive variables such as BMI and smoking status. Missing values were addressed using multiple imputations using chained equations.

Figure 2. Aortic stiffness at baseline, their hazard ratios and discriminative ability for cardiovascular disease.

Values are given as the median and interquartile range (IQR), or No. of cases (%). Baseline data of 714 patients are included. All models are stratified by recruitment site.

[a]Adjusted for age and sex.

[b]Adjusted for Framingham risk factors: age, sex, smoking, high-density lipoprotein, total cholesterol, systolic blood pressure, diabetes, hypertension medication.

PWV further adjusted for mean arterial pressure and resting heart rate. AIx further adjusted for resting heart rate and height.

CI = confidence interval. PWV = pulse wave velocity. CIMT = carotid intima-media thickness. AIx = augmentation index.

There were about 10% missing values for variables CIMT (n = 66) and PWV (n = 60). Missing values were addressed using multiple imputations using chained equations.

Figure 3. Alternative measures at baseline, their hazard ratios and discriminative ability for cardiovascular disease.

For peer review only

Values are given as the median and interquartile range (IQR), or No. of cases (%). Baseline data of 714 patients are included. All models are stratified by recruitment site.

[a]Adjusted for age and sex.

[b]Adjusted for Framingham risk factors: age, sex, smoking, high-density lipoprotein, total cholesterol, systolic blood pressure, diabetes, hypertension medication.

CI = confidence interval. CRP = C-reactive protein. BMI = body mass index. GOLD = global initiative for chronic obstructive lung disease. 4MGS = four-metre gait speed. 6MWT = six-minute walk test. BODE = body mass index, obstruction, dyspnoea, exercise.

There were < 5% missing values for biochemical markers including fibrinogen and cholesterol. Missing values were addressed using multiple imputations using chained equations.

For peer review only

Online supplementary material

Supplementary text 1 Supplementary methods.

Supplementary table 1 Definitions of diagnoses by ICD-10 coding.

Supplementary table 2 Framingham risk factors at baseline, their hazard ratios for cardiovascular disease.

Supplementary table 3 Arterial stiffness at baseline, their hazard ratios for cardiovascular disease.

Supplementary table 4 Alternative measures at baseline, their hazard ratios for cardiovascular disease.

Supplementary figure 1 Percentage of missing values.

Supplementary figure 2 Percentage and pattern of missing values in key baseline characteristics.

Supplementary figure 3 Differences in baseline pulse wave velocity.

Supplementary figure 4 Differences in baseline carotid intima-media thickness.

Supplementary figure 5 Differences in baseline augmentation index.

Supplementary figure 6 Participant enrolment flow diagram with five years follow up.

For peer review only

Risk factor	Median (IQR) or No. (%)	HR (95% CI)[a]	HR (95% CI)[b]	C−statistic
All Framingham risk factors				0.696 [0.691 to 0.701]
Age − per 10 year increase	67 (62−73)	1.50 (1.26 to 1.78)	1.43 (1.18 to 1.72)	0.594 [0.588 to 0.602]
Sex − male	434 (61)	1.16 (0.88 to 1.52)	1.12 (0.84 tot 1.49)	0.551 [0.542 to 0.558]
Smoking − current	218 (31)	0.93 (0.70 to 1.25)	0.91 (0.68 to 1.23)	0.537 [0.528 to 0.544]
HDL − per 1 mmol/L increase	1.4 (1.2−1.7)	0.61 (0.44 to 0.84)	0.91 (0.66 to 1.27)	0.569 [0.563 to 0.577]
Cholesterol − per 1 mmol/L increase	5.0 (4.3−5.8)	0.72 (0.63 to 0.81)	0.88 (0.76 to 1.01)	0.618 [0.611 to 0.623]
SBP − per 10 mmHg increase	142 (131−154)	0.92 (0.85 to 0.99)	0.93 (0.86 to 1.00)	0.540 [0.532 to 0.547]
Diabetes − yes	82 (12)	4.16 (3.09 to 5.60)	3.23 (2.32 to 4.50)	0.616 [0.607 to 0.622]
Hypertension medication − yes	245 (34)	1.77 (1.37 to 2.29)	1.50 (1.15 to 1.96)	0.612 [0.604 to 0.616]

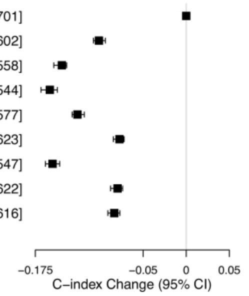

Figure 1. Framingham risk factors at baseline, their hazard ratios and discriminative ability for cardiovascular disease.Values are given as the median and interquartile range (IQR), or No. of cases (%). Baseline data of 714 patients are included. All models are stratified by recruitment site.[a]Adjusted for age and sex.[b]Adjusted for Framingham risk factors: age, sex, smoking, high-density lipoprotein, total cholesterol, systolic blood pressure, diabetes, hypertension medication.CI = confidence interval. HDL = high-density lipoprotein. SBP = systolic blood pressure. CV = cardiovascular.There were < 5% missing values for descriptive variables such as BMI and smoking status. Missing values were addressed using multiple imputations using chained equations.

309x95mm (300 x 300 DPI)

Risk factor	Median (IQR) or No. (%)	HR (95% CI)[a]	HR (95% CI)[b]	C−statistic
All Framingham risk factors				0.696 [0.691 to 0.701]
+ PWV – per 1 m/sec increase	9.8 (8.4–11.8)	1.04 (0.98 to 1.10)	0.99 (0.93 to 1.06)	0.695 [0.690 to 0.700]
+ CIMT – per 1 mm increase	0.81 (0.71–0.96)	1.18 (0.57 to 2.44)	1.29 (0.60 to 2.77)	0.694 [0.689 to 0.700]
+ AIx – per 5% increase	28 (20–34)	0.84 (0.78 to 0.92)	0.92 (0.84 to 1.01)	0.695 [0.690 to 0.700]

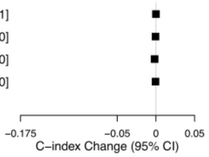

Figure 2. Aortic stiffness at baseline, their hazard ratios and discriminative ability for cardiovascular disease.Values are given as the median and interquartile range (IQR), or No. of cases (%). Baseline data of 714 patients are included. All models are stratified by recruitment site.[a]Adjusted for age and sex.[b]Adjusted for Framingham risk factors: age, sex, smoking, high-density lipoprotein, total cholesterol, systolic blood pressure, diabetes, hypertension medication.PWV further adjusted for mean arterial pressure and resting heart rate. AIx further adjusted for resting heart rate and height.CI = confidence interval. PWV = pulse wave velocity. CIMT = carotid intima-media thickness. AIx = augmentation index.There were about 10% missing values for variables CIMT (n = 66) and PWV (n = 60). Missing values were addressed using multiple imputations using chained equations.

343x66mm (300 x 300 DPI)

Risk factor	Median (IQR) or No. (%)	HR (95% CI)[a]	HR (95% CI)[b]	C−statistic
All Framingham risk factors				0.696 [0.691 to 0.701]
+ CRP – per twofold increase	1.21 (0.47 to 2.01)	1.20 (1.07 to 1.35)	1.17 (1.04 to 1.31)	0.698 [0.693 to 0.703]
+ Fibrinogen – per twofold increase	1.22 (1.06 to 1.36)	1.75 (1.14 to 2.67)	1.75 (1.14 to 2.68)	0.695 [0.690 to 0.700]
+ Glucose – per twofold increase	1.59 (1.46 to 4.59)	2.55 (1.43 to 4.53)	1.94 (1.05 to 3.56)	0.695 [0.690 to 0.700]
+ BMI – per 1 kg/m^2 increase	27 (23–31)	1.07 (1.04 to 1.09)	1.04 (1.02 to 1.07)	0.705 [0.700 to 0.711]
+ GOLD – per 1 stage increase	1 (1–2)	1.19 (0.98 to 1.45)	1.28 (1.04 to 1.57)	0.697 [0.691 to 0.701]
+ 4MGS – per 1 second increase	4.2 (3.5 to 5.2)	1.10 (1.05 to 1.16)	1.08 (1.02 to 1.15)	0.713 [0.707 to 0.718]
+ 6MW distance – per 30 meter increase	366 (255 to 440)	0.91 (0.88 to 0.93)	0.91 (0.88 to 0.94)	0.725 [0.720 to 0.731]
+ BODE – per 1 point increase	3 (1–5)	1.13 (1.07 to 1.18)	1.14 (1.08 to 1.20)	0.711 [0.705 to 0.716]
+ BMI, 4MGS, 6MW, BODE – per 1 unit increase				0.727 [0.721 to 0.733]

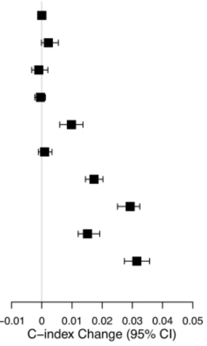

Figure 3. Alternative measures at baseline, their hazard ratios and discriminative ability for cardiovascular disease.Values are given as the median and interquartile range (IQR), or No. of cases (%). Baseline data of 714 patients are included. All models are stratified by recruitment site.[a]Adjusted for age and sex.[b]Adjusted for Framingham risk factors: age, sex, smoking, high-density lipoprotein, total cholesterol, systolic blood pressure, diabetes, hypertension medication.CI = confidence interval. CRP = C-reactive protein. BMI = body mass index. GOLD = global initiative for chronic obstructive lung disease. 4MGS = four-metre gait speed. 6MWT = six-minute walk test. BODE = body mass index, obstruction, dyspnoea, exercise.There were < 5% missing values for biochemical markers including fibrinogen and cholesterol. Missing values were addressed using multiple imputations using chained equations.

331x119mm (300 x 300 DPI)

Supplementary materials for “Physical performance measures improves cardiovascular risk prediction in COPD: a multi-centre prospective UK study.”

Jilles M. Fermont, Marie Fisk, Charlotte E. Bolton, William Macnee, John Cockcroft, Jonathan Fuld, Joseph Cheriyan, Divya Mohan, Kaisa M. Mäki-Petäjä, Ali B.A.K. Al-Hadithi, Ruth Tal-Singer, Hana Müllerova, Michael I. Polkey, Angela M. Wood, Carmel McEniery, Ian B. Wilkinson, on behalf of the ERICA consortium.

For peer review only

Supplementary text

Text 1 Supplementary methods

There were missing values. Data were assessed for the level and type of missing data, and completion patterns (Figures 1-2). There were about 10% missing values for variables CIMT (n = 66) and PWV (n = 60), with <5% missing values for other variables (Figures 3-5). Missing values were addressed using multiple imputations using chained equations (MICE). The time-to-event outcome was included using the non-parametric Nelson-Aalen estimator. Predictive mean matching was used for continuous variables, ordered logistic regression (as continuous) for ordinal variables, multinomial logistic regression for categorical variables, and logistic regression for binary variables. Derived variables such as the BODE Index (a composite score of BMI, forced expiratory volume in one second (FEV_1), Medical Research Council dyspnoea score, and 6MWT distance) and GOLD stage were estimated post MICE using passive imputation.

For peer review only

Supplementary tables

Table 1 Definitions of diagnoses by ICD-10 coding.

End point	ICD-10 codes
**All cardiovascular disease**	**E10.5, E11-E14, F01, G46.3-G46.7, G458, G459 I11.0, I13.0, I13.2, I20.0-I20.1, I20.8-I21-I25, I50, I60, I61, I.62, I63, I64, I65-I69, I70.2, I71.3-I71.9, I72, I73.9-I79, R96 + cardiac death**
**Diseases of the arteries**	**I70.2, I72, I73.9-I79, E10.5, E11-E14**
Peripheral arterial disease	I70.2, I73.9, E10.5, E11-E14
Diseases of arteries, arterioles and capillaries	I72, I74-I79
**Coronary heart disease**	**I20.0-I20.1, I20.8-I21-I25**
Angina	I20.1, I20.8-I20.9
Unstable angina	I20.0, I24
Coronary heart disease not otherwise specified	I25
Acute myocardial infarction (MI), and certain current complications following acute MI	I21, I23
Subsequent myocardial infarction	I22
**All stroke**	**I60, I61, I.62, I63, I64, I65-I69, F01, G46.3-G46.7, G458, G459**
Subarachnoid haemorrhage	I60
Intra-cerebral haemorrhage	I61
Cerebral infarction	I63
Stroke, not specified as haemorrhage or infarction	I64
Stroke syndromes	G46.3-G46.7
Transient ischemic attack	G458, G459
Other stroke	I62, I65-I69, F01
**Heart failure**	**I11.0, I13.0, I13.2, I50**
Heart failure	I50
Hypertensive heart disease with (congestive) heart failure	I11.0
Hypertensive heart and renal disease with (congestive) heart failure	I13.0
Hypertensive heart and renal disease with both (congestive) heart failure and renal disease	I13.2
**Cardiac death**	**Adjudicated**
**Other vascular deaths**	**R96, I71.3-I71.9**
Sudden death, cause unknown	R96
Abdominal aortic aneurysm	I71.3-I71.9

Atrial fibrillation and flutter (I48) and hypertensive diseases (I10-I15) were considered risk factors and therefore not included.

For peer review only

Table 2 Framingham risk factors at baseline, their hazard ratios for cardiovascular disease.

	Median (IQR) or No. (%)	HR (95% CI)[a]	P value	HR (95% CI)[b]	P value
**Framingham risk factors**					
Age – per 10 year increase	67 (62-73)	1.50 (1.26 to 1.78)	< 0.001	1.43 (1.18 to 1.72)	< 0.001
Sex – males	434 (61)	1.16 (0.88 to 1.52)	0.282	1.12 (0.84 tot 1.49)	0.437
Smoking – current	218 (31)	0.93 (0.70 to 1.25)	0.650	0.91 (0.68 to 1.23)	0.554
HDL – per 1 mmol/L increase	1.4 (1.2-1.7)	0.61 (0.44 to 0.84)	0.003	0.91 (0.66 to 1.27)	0.596
Total cholesterol – per 1 mmol/L increase	5.0 (4.3-5.8)	0.72 (0.63 to 0.81)	< 0.001	0.88 (0.76 to 1.01)	0.063
SBP – per 10 mmHg increase	142 (131-154)	0.92 (0.85 to 0.99)	0.029	0.93 (0.86 to 1.00)	0.061
Diabetes – yes	82 (12)	4.16 (3.09 to 5.60)	< 0.001	3.23 (2.32 to 4.50)	< 0.001
Hypertension treatment – yes	245 (34)	1.77 (1.37 to 2.29)	< 0.001	1.50 (1.15 to 1.96)	0.003

Values are given as the median and interquartile range (IQR), or No. of cases (%). Baseline data of 714 patients are included. All models are stratified by recruitment site.
[a]Adjusted for age and sex
[b]Adjusted for Framingham risk factors: age, sex, smoking, high-density lipoprotein, total cholesterol, systolic blood pressure, diabetes, hypertension drug treatment.
CI = confidence interval. HDL = high-density lipoprotein. SBP = systolic blood pressure. CV = cardiovascular.

For peer review only

Table 3 Arterial stiffness at baseline, their hazard ratios for cardiovascular disease.

	Median (IQR) or No. (%)	HR (95% CI)[a]	P value	HR (95% CI)[b]	P value
**Measures of arterial stiffness**					
PWV – per 1 m/sec increase	9.8 (8.4-11.8)	1.04 (0.98 to 1.10)	0.159	0.99 (0.93 to 1.06)	0.839
CIMT – per 1 mm increase	0.81 (0.71-0.96)	1.18 (0.57 to 2.44)	0.651	1.29 (0.60 to 2.77)	0.519
AIx – per 5% increase	28 (20-34)	0.84 (0.78 to 0.92)	< 0.001	0.92 (0.84 to 1.01)	0.056

Values are given as the median and interquartile range (IQR), or No. of cases (%). Baseline data of 714 patients are included. All models are stratified by recruitment site.
[a]Adjusted for age and sex
[b]Adjusted for Framingham risk factors: age, sex, smoking, high-density lipoprotein, total cholesterol, systolic blood pressure, diabetes, hypertension drug treatment. PWV further adjusted for mean arterial pressure and resting heart rate. AIx further adjusted for resting heart rate and height.
CI = confidence interval. PWV = pulse wave velocity. CIMT = carotid intima-media thickness. AIx = augmentation index.

For peer review only

Table 4 Alternative measures at baseline, their hazard ratios for cardiovascular disease.

	Median (IQR) or No. (%)	HR (95% CI)[a]	P value	HR (95% CI)[b]	P value
**Other risk factors**					
CRP – per twofold increase	1.21 (0.47 to 2.01)	1.20 (1.07 to 1.35)	0.002	1.17 (1.04 to 1.31)	0.010
Fibrinogen – per twofold increase	1.22 (1.06 to 1.36)	1.75 (1.14 to 2.67)	0.009	1.75 (1.14 to 2.68)	0.010
Glucose – per twofold increase	1.59 (1.50 to 1.69)	2.55 (1.43 to 4.53)	0.001	1.94 (1.05 to 3.56)	0.033
BMI – per 1 kg/m^2 increase	27 (23-31)	1.07 (1.04 to 1.09)	< 0.001	1.04 (1.02 to 1.07)	0.001
GOLD – per 1 stage increase	1 (1-2)	1.19 (0.98 to 1.45)	0.077	1.28 (1.04 to 1.57)	0.018
4MGST – per 1 second increase	4.2 (3.5 to 5.2)	1.10 (1.05 to 1.16)	< 0.001	1.08 (1.02 to 1.15)	0.010
6MWT distance – per 30 metre increase	366 (255 to 440)	0.91 (0.88 to 0.93)	< 0.001	0.91 (0.88 to 0.94)	< 0.001
BODE – per 1 point increase	3 (1-5)	1.13 (1.07 to 1.18)	< 0.001	1.14 (1.08 to 1.20)	< 0.001

Values are given as the median and interquartile range (IQR), or No. of cases (%). Baseline data of 714 patients are included. All models are stratified by recruitment site.
[a]Adjusted for age and sex
[b]Adjusted for Framingham risk factors: age, sex, smoking, high-density lipoprotein, total cholesterol, systolic blood pressure, diabetes, hypertension drug treatment.
CI = confidence interval. CRP = C-reactive protein. BMI = body mass index. GOLD = global initiative for chronic obstructive lung disease. 4MGS = four-metre gait speed. 6MWT = six-minute walk test. BODE = body mass index, obstruction, dyspnoea, exercise.

For peer review only

Supplementary figures

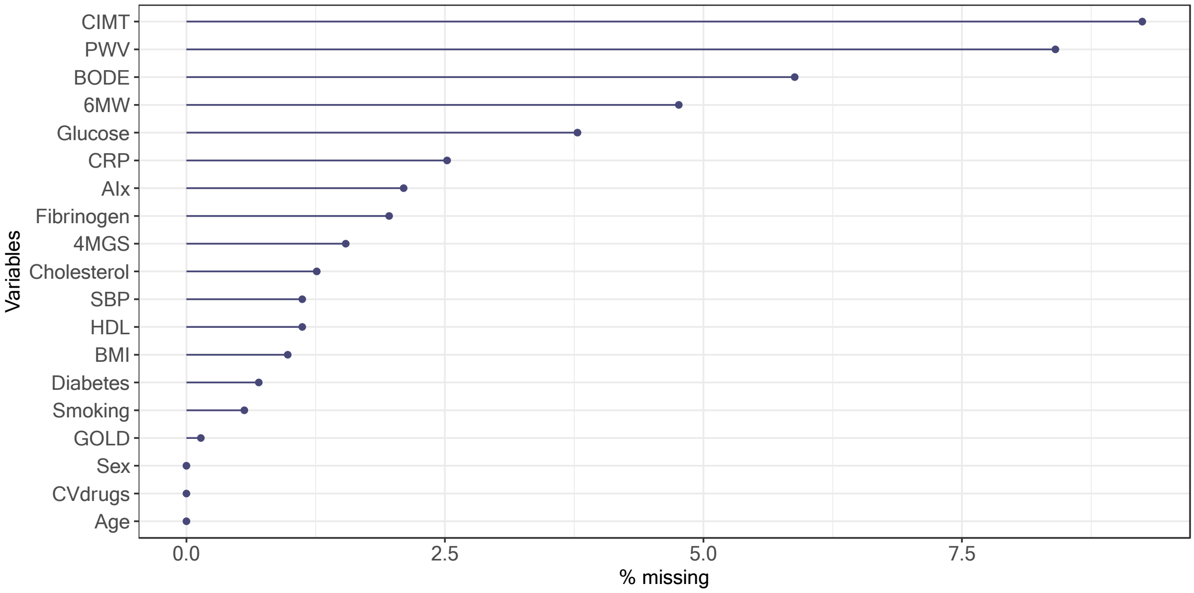

Figure 1 Percentage of missing values.
CIMT = carotid intima-media thickness. PWV = pulse wave velocity. BODE = body mass index, obstruction (i.e. forced expiratory volume in one second), dyspnoea score, exercise (i.e. six-minute walk test distance). 6MWT = six-minute walk test. CRP = C-reactive protein. AIx = augmentation index. 4MGS = four-metre gait speed. SBP = systolic blood pressure. HDL = high-density lipoprotein. BMI = body mass index. GOLD = global initiative for obstructive lung disease. CV = cardiovascular.

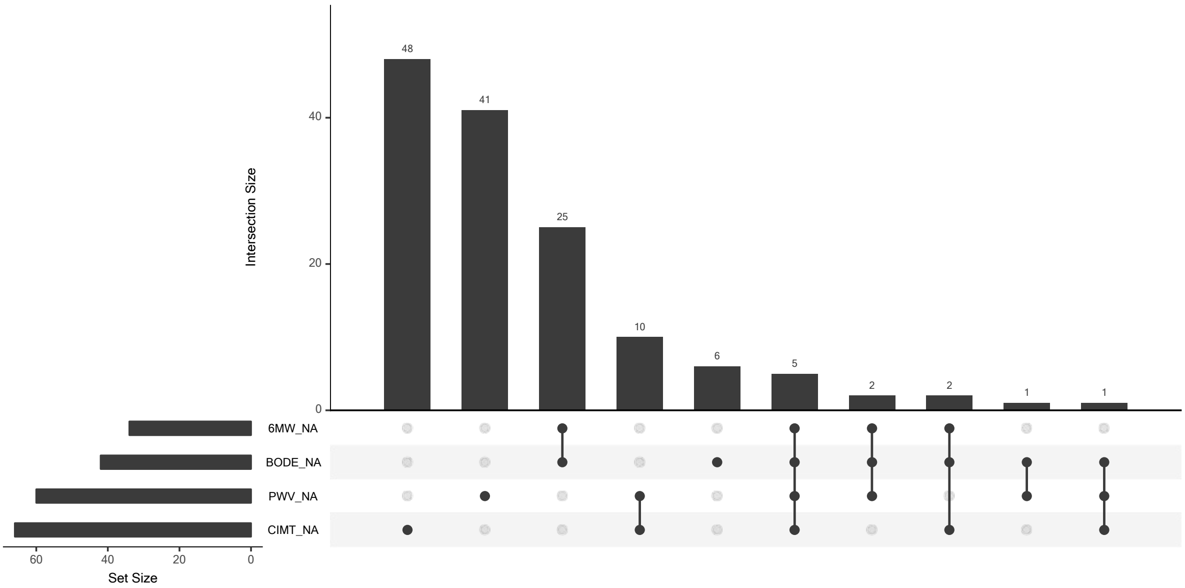

Figure 2 Percentage and pattern of missing values in key baseline characteristics.
6MWT, six-minute walk test. BODE = body mass index, obstruction (i.e. forced expiratory volume in one second), dyspnoea score, exercise (i.e. six-minute walk test distance). PWV, pulse wave velocity. CIMT, carotid-intima media thickness.

For peer review only - http://bmjopen.bmj.com/site/about/guidelines.xhtml

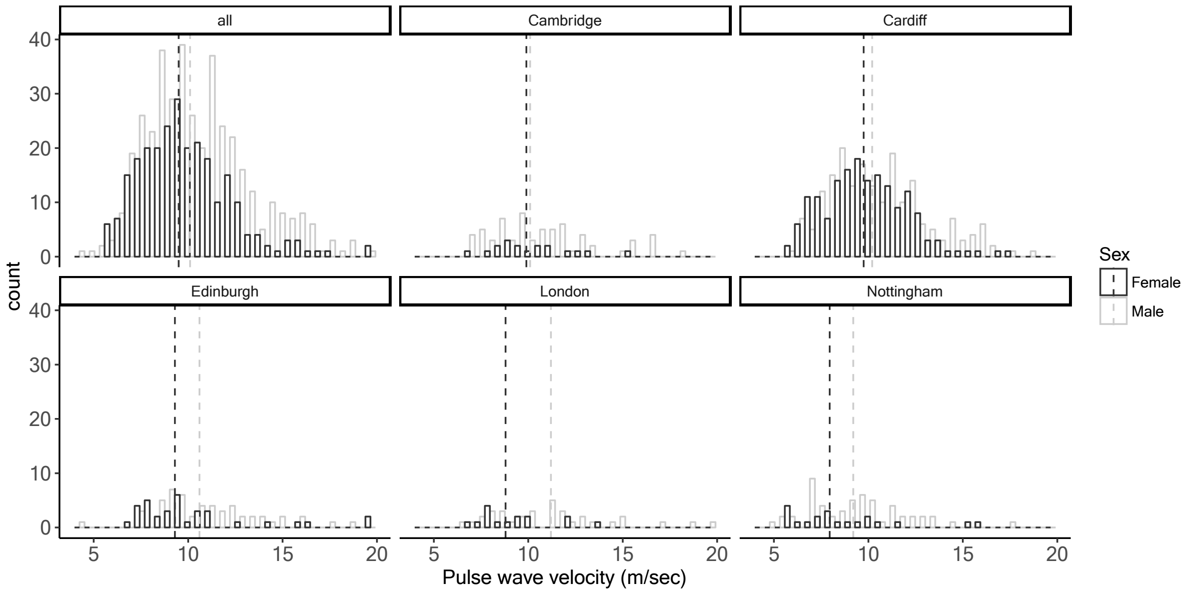

Figure 3 Differences in baseline pulse wave velocity.
Histograms displaying the distribution of PWV by sex and recruitment site. Dashed lines indicate median values by sex. Where only one median line is visible, medians for both sexes are similar.

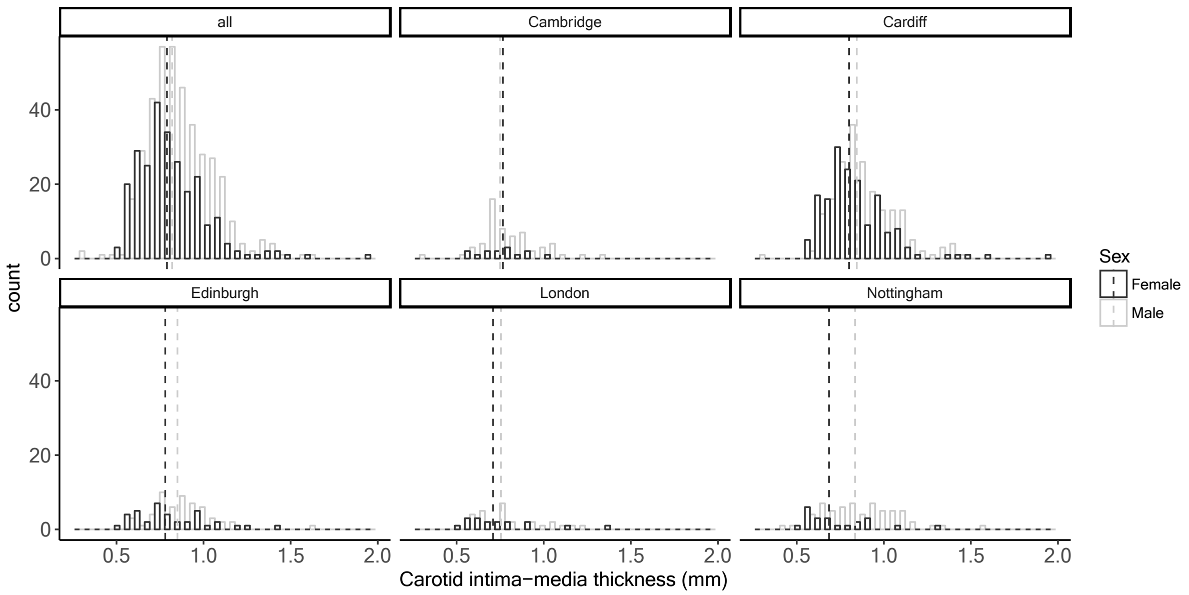

Figure 4 Differences in baseline carotid intima-media thickness.
Histograms displaying the distribution of CIMT by sex and recruitment site. Dashed lines indicate median values by sex. Where only one median line is visible, medians for both sexes are similar.

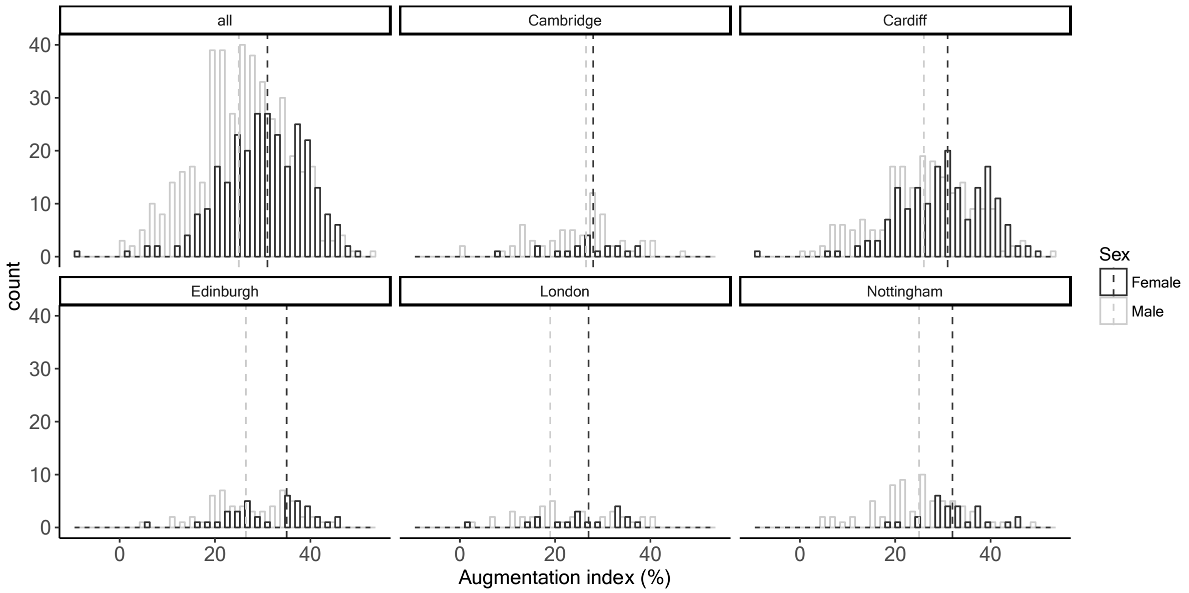

Figure 5 Differences in baseline augmentation index.
Histograms displaying the distribution of AIx by sex and recruitment site. Dashed lines indicate median values by sex.

For peer review only - http://bmjopen.bmj.com/site/about/guidelines.xhtml

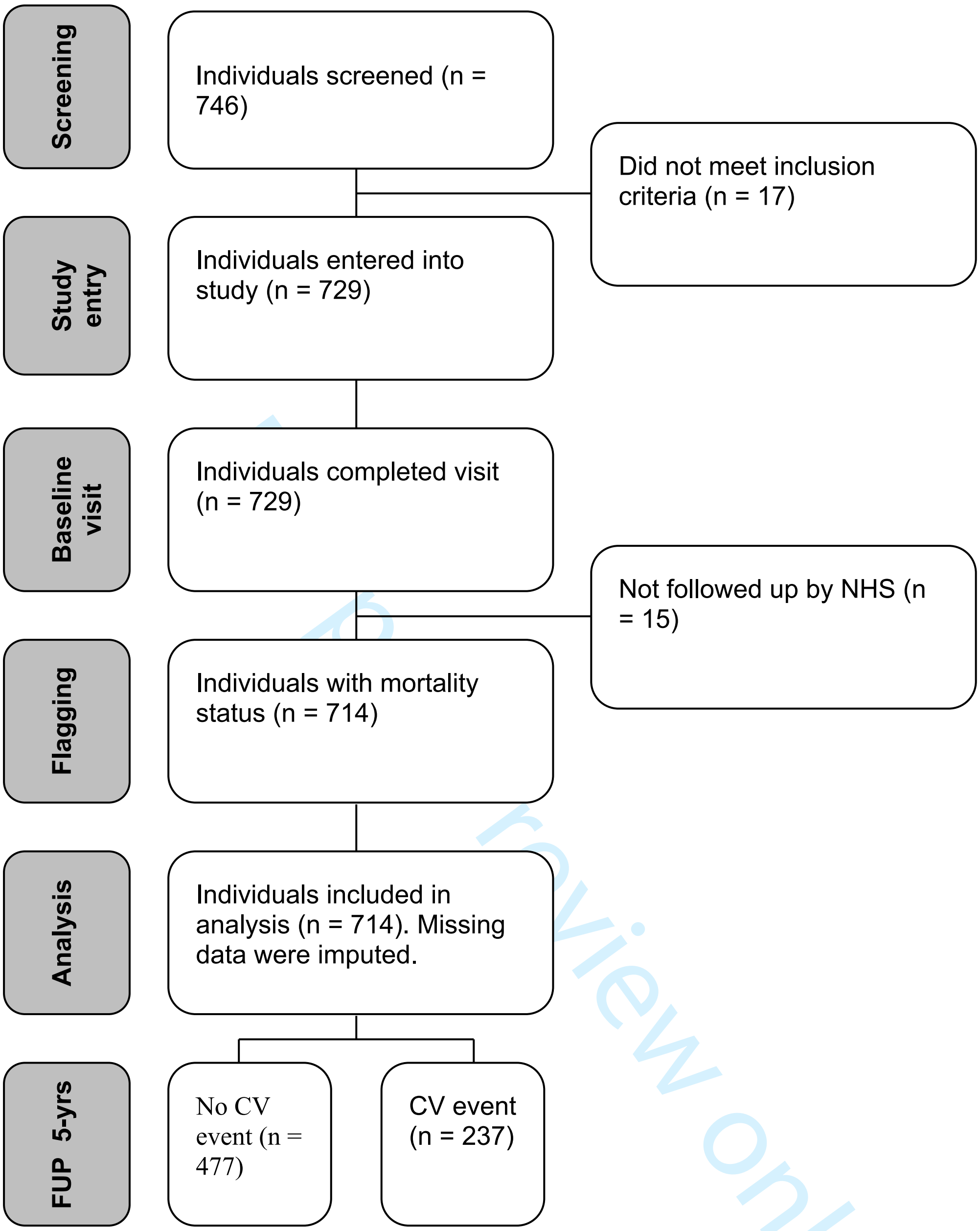

Figure 6 Participant enrolment flow diagram with five years follow up.
Total number of CV events (n = 237): peripheral arterial disease (n = 77), diseases of arteries, arterioles and capillaries (n = 6), angina (n = 24), unstable angina (n = 2), coronary heart disease not otherwise specified (n = 58), acute myocardial infarction (MI), and certain current complications following acute MI (n = 8), cerebral infarction (n = 9), stroke, not specified as haemorrhage or infarction (n = 1), other stroke (n = 14), heart failure (n = 28), and abdominal aortic aneurysm (n = 5). FEV_1 = forced expiratory volume one second. FVC = forced vital capacity. NHS = National Health Services. FUP = follow-up period.

STROBE 2007 (v4) Statement—Checklist of items that should be included in reports of *cohort studies*

**Section/Topic**	**Item #**	**Recommendation**	**Reported on page #**
**Title and abstract**	1	(*a*) Indicate the study's design with a commonly used term in the title or the abstract	1
		(*b*) Provide in the abstract an informative and balanced summary of what was done and what was found	3
**Introduction**			
Background/rationale	2	Explain the scientific background and rationale for the investigation being reported	6
Objectives	3	State specific objectives, including any prespecified hypotheses	7
**Methods**			
Study design	4	Present key elements of study design early in the paper	8
Setting	5	Describe the setting, locations, and relevant dates, including periods of recruitment, exposure, follow-up, and data collection	8
Participants	6	(*a*) Give the eligibility criteria, and the sources and methods of selection of participants. Describe methods of follow-up	8
		(*b*) For matched studies, give matching criteria and number of exposed and unexposed	
Variables	7	Clearly define all outcomes, exposures, predictors, potential confounders, and effect modifiers. Give diagnostic criteria, if applicable	8-9
Data sources/ measurement	8*	For each variable of interest, give sources of data and details of methods of assessment (measurement). Describe comparability of assessment methods if there is more than one group	8-9
Bias	9	Describe any efforts to address potential sources of bias	9
Study size	10	Explain how the study size was arrived at	8-9
Quantitative variables	11	Explain how quantitative variables were handled in the analyses. If applicable, describe which groupings were chosen and why	9
Statistical methods	12	(*a*) Describe all statistical methods, including those used to control for confounding	9 + supplementary text 1
		(*b*) Describe any methods used to examine subgroups and interactions	9
		(*c*) Explain how missing data were addressed	Supplementary text 1
		(*d*) If applicable, explain how loss to follow-up was addressed	

For peer review only

		(*e*) Describe any sensitivity analyses	
**Results**			
Participants	13*	(a) Report numbers of individuals at each stage of study—eg numbers potentially eligible, examined for eligibility, confirmed eligible, included in the study, completing follow-up, and analysed	10 + supplementary figure 6
		(b) Give reasons for non-participation at each stage	Supplementary figure 6
		(c) Consider use of a flow diagram	Supplementary figure 6
Descriptive data	14*	(a) Give characteristics of study participants (eg demographic, clinical, social) and information on exposures and potential confounders	10-11
		(b) Indicate number of participants with missing data for each variable of interest	Supplementary figures 1-5
		(c) Summarise follow-up time (eg, average and total amount)	10
Outcome data	15*	Report numbers of outcome events or summary measures over time	10
Main results	16	(*a*) Give unadjusted estimates and, if applicable, confounder-adjusted estimates and their precision (eg, 95% confidence interval). Make clear which confounders were adjusted for and why they were included	11-13
		(*b*) Report category boundaries when continuous variables were categorized	
		(*c*) If relevant, consider translating estimates of relative risk into absolute risk for a meaningful time period	
Other analyses	17	Report other analyses done—eg analyses of subgroups and interactions, and sensitivity analyses	
**Discussion**			
Key results	18	Summarise key results with reference to study objectives	14
**Limitations**			
Interpretation	20	Give a cautious overall interpretation of results considering objectives, limitations, multiplicity of analyses, results from similar studies, and other relevant evidence	16
Generalisability	21	Discuss the generalisability (external validity) of the study results	17
**Other information**			
Funding	22	Give the source of funding and the role of the funders for the present study and, if applicable, for the original study on which the present article is based	17-18

*Give information separately for cases and controls in case-control studies and, if applicable, for exposed and unexposed groups in cohort and cross-sectional studies.

For peer review only

Note: An Explanation and Elaboration article discusses each checklist item and gives methodological background and published examples of transparent reporting. The STROBE checklist is best used in conjunction with this article (freely available on the Web sites of PLoS Medicine at http://www.plosmedicine.org/, Annals of Internal Medicine at http://www.annals.org/, and Epidemiology at http://www.epidem.com/). Information on the STROBE Initiative is available at www.strobe-statement.org.

For peer review only

BMJ Open

Cardiovascular risk prediction using physical performance measures in COPD: results from a multi-centre observational study.

Journal:	*BMJ Open*
Manuscript ID	bmjopen-2020-038360.R1
Article Type:	Original research
Date Submitted by the Author:	25-Jun-2020
Complete List of Authors:	Fermont, Jilles; University of Cambridge, Department of Medicine<br>Fisk, Marie; University of Cambridge<br>Bolton, Charlotte; University of Nottingham<br>MacNee, William; The University of Edinburgh<br>Cockcroft, John; Columbia University Medical Center<br>Fuld, Jonathan; Cambridge University Hospitals NHS Foundation Trust, Centre for Self Management Support<br>Cheriyan, Joseph; University of Cambridge Medicine, Division of Experimental Medicine & Immunotherapeutics<br>Mohan, Divya; GSK R&D<br>Mäki-Petäjä, Kaisa ; University of Cambridge<br>Al-Hadithi, Ali ; University of Cambridge,<br>Tal-Singer, Ruth; GSK R&D<br>Müllerova, Hana; GSK R&D<br>Polkey, Mike; Royal Brompton Hospital<br>Wood, Angela; University of Cambridge<br>McEniery, Carmel M.; University of Cambridge<br>Wilkinson, Ian; University of Cambridge; Cambridge Clinical Trials Unit,
**Primary Subject Heading**:	Respiratory medicine
Secondary Subject Heading:	Cardiovascular medicine, Epidemiology, Diagnostics, Public health
Keywords:	Chronic airways disease < THORACIC MEDICINE, EPIDEMIOLOGY, RESPIRATORY MEDICINE (see Thoracic Medicine), Cardiac Epidemiology < CARDIOLOGY, PRIMARY CARE

SCHOLARONE™
Manuscripts

I, the Submitting Author has the right to grant and does grant on behalf of all authors of the Work (as defined in the below author licence), an exclusive licence and/or a non-exclusive licence for contributions from authors who are: i) UK Crown employees; ii) where BMJ has agreed a CC-BY licence shall apply, and/or iii) in accordance with the terms applicable for US Federal Government officers or employees acting as part of their official duties; on a worldwide, perpetual, irrevocable, royalty-free basis to BMJ Publishing Group Ltd ("BMJ") its licensees and where the relevant Journal is co-owned by BMJ to the co-owners of the Journal, to publish the Work in this journal and any other BMJ products and to exploit all rights, as set out in our licence.

The Submitting Author accepts and understands that any supply made under these terms is made by BMJ to the Submitting Author unless you are acting as an employee on behalf of your employer or a postgraduate student of an affiliated institution which is paying any applicable article publishing charge ("APC") for Open Access articles. Where the Submitting Author wishes to make the Work available on an Open Access basis (and intends to pay the relevant APC), the terms of reuse of such Open Access shall be governed by a Creative Commons licence – details of these licences and which Creative Commons licence will apply to this Work are set out in our licence referred to above.

Other than as permitted in any relevant BMJ Author's Self Archiving Policies, I confirm this Work has not been accepted for publication elsewhere, is not being considered for publication elsewhere and does not duplicate material already published. I confirm all authors consent to publication of this Work and authorise the granting of this licence.

For peer review only

Cardiovascular risk prediction using physical performance measures in COPD: results from a multi-centre observational study.

Jilles M. Fermont[1,2$], Marie Fisk[1], Charlotte E. Bolton[3], William Macnee[4], John Cockcroft[5], Jonathan Fuld[6], Joseph Cheriyan[1], Divya Mohan[7], Kaisa M. Mäki-Petäjä[1], Ali B.A.K. Al-Hadithi[1], Ruth Tal-Singer[7], Hana Müllerova[8], Michael I. Polkey[9], Angela M. Wood[2,10-13], Carmel McEniery[1], Ian B. Wilkinson[1,14], on behalf of the ERICA consortium.

[1]Division of Experimental Medicine and Immunotherapeutics, Department of Medicine, University of Cambridge, Cambridge, United Kingdom;

[2]British Heart Foundation Cardiovascular Epidemiology Unit, Department of Public Health and Primary Care, University of Cambridge, Cambridge, United Kingdom;

[3]Division of Respiratory Medicine and NIHR Nottingham BRC respiratory theme, University of Nottingham, Nottingham, United Kingdom;

[4]Centre for Inflammation Research, Queen's Medical Research Institute, University of Edinburgh, Edinburgh, Scotland United Kingdom;

[5]Department of Cardiology, Columbia University Medical Centre, New York, United States;

[6]Department of Respiratory Medicine, Cambridge University Hospitals NHS Foundation Trust, Cambridge, United Kingdom;

[7]Medical Innovation, Value Evidence Outcomes, GSK R&D, PA, United States;

[8]GSK R&D, Uxbridge, United Kingdom;

[9]Department of Respiratory Medicine, Royal Brompton Hospital, London, United Kingdom;

[10]British Heart Foundation Centre of Research Excellence, University of Cambridge, Cambridge, United Kingdom;

[11]National Institute for Health Research Blood and Transplant Research Unit in Donor Health and Genomics, University of Cambridge, Cambridge, United Kingdom;

[12]National Institute for Health Research Cambridge Biomedical Research Centre, University of Cambridge and Cambridge University Hospitals, Cambridge, United Kingdom;

[13]Health Data Research UK Cambridge, Wellcome Genome Campus and University of Cambridge, Cambridge, United Kingdom;

[14]Cambridge Clinical Trials Unit, Cambridge University Hospitals NHS Foundation Trust, Addenbrooke's Hospital, Cambridge, United Kingdom.

For peer review only

[$]Corresponding author: Jilles M. Fermont, Division of Experimental Medicine and Immunotherapeutics, Department of Medicine, University of Cambridge, Cambridge CB1 8RN, United Kingdom. Tel: +44 (0)12 2333 6806. E-mail: jmf88@medschl.cam.ac.uk.

Word count: 3384

Keywords: Chronic Obstructive Pulmonary Disease; COPD; Biomarkers; Prognosis; Cardiovascular Diseases.

For peer review only

ABSTRACT

Objectives Although cardiovascular disease (CVD) is a common co-morbidity associated with chronic obstructive pulmonary disease (COPD), it is unknown how to improve prediction of cardiovascular (CV) risk in COPD individuals. Traditional CV risk scores have been tested in different populations but not uniquely in COPD. The potential of alternative markers to improve CV risk prediction in COPD individuals is unknown. We aimed to determine the predictive value of conventional CVD risk factors in COPD, and to determine if additional markers improve prediction beyond conventional factors.

Design Data from the ERICA cohort, which enrolled 729 GOLD stage II-IV COPD individuals were used. Linked hospital episode statistics and survival data were prospectively collected for a median 4.5 years follow-up.

Setting Five UK centres with an interest in COPD.

Participants Population-based sample including 714 individuals with spirometry-defined COPD, at least ten pack years and who were clinically stable for more than four weeks.

Interventions Baseline measurements included aortic pulse wave velocity (aPWV), carotid intima media thickness (CIMT), C-reactive protein (CRP), fibrinogen, spirometry and BODE-Index, six-minute walk test (6MWT) and four-metre gait speed (4MGS) test.

Primary and secondary outcome measures New occurrence (first event) of fatal or non-fatal hospitalised CVD, and all-cause and cause-specific mortality.

Results Out of 714 participants, 232 (32%) had CV-hospitalisation and five died due to CVD. The overall CV risk model C-statistic was 0.696 (95% CI 0.691 to 0.701). Aortic pulse wave velocity and CIMT had no association with study outcome; nor improved model prediction. CRP, fibrinogen, BODE Index, 4MGS and 6MWT were associated with the outcome; independently of conventional risk factors ($p < 0.05$ for all). However, only 6MWT improved model discrimination (C = 0.725 [0.720 to 0.731]).

For peer review only

Conclusions Poor physical performance defined by the 6MWT improves prediction of CV hospitalisation in COPD individuals.

Trial registration Clinical Trials Gateway (ID 11101).

For peer review only

ARTICLE SUMMARY

Strengths and limitations of this study

- This is the first study assessing the utility of conventional cardiovascular (CV) disease risk factors for CV risk prediction and the value of additional markers of risk within a COPD cohort.
- Patient level cohort data were linked to hospital admission data (i.e. hospital episode statistics) obtained from the National Health Services in England, Scotland and Wales, and Office for National Statistics record of mortality with analyses limited to five years of follow-up.
- Hospitalised cardiovascular episodes were coded based on ICD-10 classifications extracted from both primary and secondary positions.
- A multivariable prediction model, with 10-fold cross validation and 200 replications, was used to evaluate a wide range of cardiovascular and physical performance biomarkers.
- Generalisability of our results is limited to those with moderate COPD in the UK with NHS hospitalisations.

For peer review only

For peer review only - http://bmjopen.bmj.com/site/about/guidelines.xhtml

INTRODUCTION

Chronic obstructive pulmonary disease (COPD) is associated with a two to three-fold increased risk of coronary artery disease and other cardiovascular (CV) comorbidities.[1,2] COPD and cardiovascular disease (CVD) are common conditions that share important common risk factors, such as smoking and physical inactivity, and both tend to affect older people. In addition, both conditions are often associated with raised systemic inflammatory markers and increased arterial stiffness.[3,4]

Given the global healthcare burden associated with CVD,[5] there is incentive to improve the accuracy of CV risk prediction in different populations. Individuals with COPD may be considered constitutively at high CV risk given their age and smoking history. However, it is unknown whether classic CV risk prediction models such as the Framingham general CV risk score, which predicts an individual's ten year risk of developing CVD based on an algorithm of weighted risk factors and has been tested in a number of different populations,[6–9] performs well in individuals with COPD.[10,11]

Conceptually, the performance of the Framingham model might be improved by additional measures. Candidate measurements include surrogate CV risk markers that impart mechanistic information (i.e. aortic pulse wave velocity (aPWV) or carotid intima-media thickness (CIMT)), inflammatory markers (i.e. C-reactive protein (CRP)) or measures of physical performance enhance CV risk prediction in COPD individuals. In fact, the value of these measures in CV risk prediction have been explored in different population groups. For example, the six-minute walk test (6MWT) significantly improved risk prediction in patients with stable coronary disease.[12] In intermediate CV risk individuals without CVD, adding CRP or fibrinogen to conventional risk factors, modestly improved CV event prediction.[13] A meta-analysis of aPWV studied in different disease cohorts, showed it improved CV event prediction independently of conventional risk factors.[14] In contrast, CIMT measurement, although associated with CV risk factors, did not significantly improve risk prediction beyond traditional factors in individuals with hypertension.[15] Given that individuals with COPD, in addition to having high CV risk based on conventional factors, also have increased aPWV and CIMT[4] increased inflammatory markers,[16] reduced lung function that is associated with increased CV risk in the general population,[17] and reduced physical performance,[18,19] the clinical significance of these findings in relation to CV risk prediction is an important question to address.

For peer review only - http://bmjopen.bmj.com/site/about/guidelines.xhtml

The aims of our study were to firstly determine the predictive value of conventional CVD risk factors for CV risk, defined by new occurrence (first event since study enrolment) of fatal or non-fatal hospitalised CVD in individuals with stable global initiative for chronic obstructive lung disease (GOLD) stage II-IV[20] COPD. The secondary aim was to determine whether addition of alternative CV measures (i.e. aPWV and CIMT), inflammatory markers (i.e. CRP and fibrinogen), COPD severity (i.e. GOLD stage and BODE Index), as well as physical performance tests commonly used in COPD research studies (i.e. 6MWT and four-metre gait speed (4MGS)) improved the predictive value of a CV risk model based on conventional CVD risk factors for CV risk prediction. We addressed these questions in the Evaluation of the Role of Inflammation in Chronic Airways disease (ERICA) COPD cohort using study data and linked UK electronic health records.

For peer review only

METHODS

Study design and participants

The ERICA study is a multi-centre observational, cohort study with 729 individuals with stable GOLD stage II-IV[20] COPD, established to identify important CV and physical performance biomarkers that could be targeted to improve the outcomes of COPD individuals. Participants had a clinical diagnosis of COPD, smoking history of at least ten pack years, FEV_1 / FVC ratio <0.7 and FEV_1 ≤80% of predicted normal lung function, and were aged >40 years old and clinically stable for >4 weeks. Full details of the protocol have been provided elsewhere.[21] Baseline data captured included demographics, spirometry, blood circulating biochemical markers, measures of arterial stiffness (i.e. aPWV and augmentation index (AIx)), CIMT and physical performance (i.e. 4MGS and 6MWT). Individuals in the ERICA study were linked with UK National Health Services (NHS) electronic healthcare records (i.e. hospital episode statistics (HES)) and Office for National Statistics death data through anonymised IDs provided by the NHS.

Clinical measures

After four hours of fasting, with no bronchodilators for six hours, and ten minutes of supine rest, carotid-femoral aPWV and AIx measurements were taken using a SphygmoCor system as previously described.[22] Carotid intima-media thickness of the common carotid arteries was measured using B-mode ultrasound at a distance of one cm from the carotid bulb with a 7–12 MHz linear probe.[23] The thickest artery of the two was included in the analysis. Systemic inflammatory markers of plasma fibrinogen and serum CRP were obtained after fasting. Physical performance measures 6MWT[24] and 4MGS[25] were assessed according to guidelines. Diabetes status and anti-hypertensive treatment were self-reported at baseline study visit. Disease severity was defined according to the GOLD classification.[20] Points for the BODE (Body mass index (BMI), airflow Obstruction, Dyspnoea, and Exercise capacity) Index were assigned as described by Celli *et al*.[26]

Cardiovascular hospitalisation and mortality

Cardiovascular hospitalisation and mortality data were extracted from the linked hospital admission data and death certificates. Non-fatal CV episodes were extracted from both primary and secondary international statistical classification of diseases and related health problems 10th revision coding

For peer review only

(ICD-10) positions. Causes of death were adjudicated by CV and pulmonary physicians. We defined the primary outcome as first reported occurrence (since study enrolment) of fatal or non-fatal hospitalised CVD, where CVD was defined as diseases of the arteries, stroke or heart failure (see supplementary table 1) based on classifications used by the Emerging Risk Factors Collaboration.[27] Time to primary outcome was calculated from the difference between the baseline visit date (starting December 2011) and either the date of death or first hospitalised CV attendance up to November 2017, when follow-up discontinued. Secondary outcomes of interest were all-cause and cause-specific mortality (defined as CV, pulmonary, cancer, or other).

Risk factors of interest

Conventional CVD risk factors included age, sex, self-reported smoking status [current/ex-smoker], HDL cholesterol, total cholesterol, systolic blood pressure (SBP), diabetes [yes/no] and treatment for high blood pressure [yes/no]. We assessed adding the following risk factors: BMI, aPWV and CIMT, bloods (fasting glucose), blood inflammatory biomarkers (i.e. CRP and fibrinogen), COPD severity (i.e. GOLD stage and BODE Index), and measures of physical performance (i.e. 6MWT and 4MGS).

Statistical analysis

Hazard ratios with 95% confidence intervals (CI) were estimated using Cox regression models stratified by study centre. Age and sex were added to all models. To quantify the independent association of CIMT we further included mean arterial pressure and resting heart. For AIx we further included resting heart rate and height. Proportional hazards were assessed by Schoenfeld's global tests. We assessed the relationships of the new markers and outcomes and consequently log-transformed the following risk factors: CRP, fibrinogen and glucose. Hazard ratios for log-transformed risk factors represent a twofold increase in the risk factor, whereas others are presented as a change in unit. We evaluated the predictive value of new markers added to the conventional CVD risk factors using measures of discrimination (i.e. Harrell’s C-statistic)[28,29] and calibration (i.e. Hosmer-Lemeshow test and Brier score). The C-statistic is a measure for validating the discriminative ability of a model. Value ranges from 0.5-1.0, whereas 1.0 is perfect prediction and 0.5 a random guess. Higher score indicates better discriminative ability of the model. To optimise efficiency and avoiding optimism

For peer review only

from internal validation in small samples, we used 10-fold cross validation with 200 replications.[30] See online supplementary text 1 and supplementary figures 1-5 for further statistical analyses details.

Observational data is reported according to the STROBE statement.[31] All tests were two-sided and statistical significance were defined by 95% CI for hazard ratios not traversing 1 or p<0.05. Our analyses were performed using Stata version 13 (College Station. Texas) and R (R Foundation).

For peer review only

RESULTS

Of the 729 individuals included in the study, 714 (98%) could be linked with hospital admission and survival records, and were included in the analysis (online supplementary figure 6). The median age was 67 (interquartile range 62-73) years and 434 (61%) individuals were male (table 1). A third (n = 218) of the cohort smoked, 12% (n = 82) had self-reported diabetes, 34% (n = 245) were taking anti-hypertensive medications, and 31% (n = 224) were taking cholesterol-lowering medications at baseline. The median FEV_1 was 1.3 (0.9-1.7) litres (mean ± SD = 1.34 ± 0.53). In total, 232 individuals (32%) had a first event of CV hospitalisation[1] (n = 189 (81%) were in ICD-10 secondary coding position) during median follow-up for 4.5 years and five individuals had CV death without any preceding CV episode. Cardiovascular hospitalisation accounted for the majority (98%) of events analysed. The CV incidence rate was 8.8 (95% CI 7.7 to 10.0) per 100 person-years. See online supplementary tables 1-2 for categorisation of different admission codes for CV hospitalisation.

Table 1 Baseline characteristics (No. = 714).

Characteristic	Summary measures
**Conventional CVD risk factors**	
Age (yrs.)	67 (62 to 73)
Male	434 (61)
Current smoker	218 (31)
HDL (mmol/L)	1.4 (1.2 to 1.7)
Cholesterol (mmol/L)	5.0 (4.3 to 5.8)
SBP (mmHg)	142 (131 to 154)
Diabetes mellitus	82 (12)
Drugs to treat hypertension	245 (34)
**CV measures**	
aPWV (m/sec)	9.8 (8.4 to 11.8)
CIMT (mm)	0.81 (0.71 to 0.96)

[1] Peripheral arterial disease (n = 77), diseases of arteries, arterioles and capillaries (n = 6), angina (n = 24), unstable angina (n = 2), coronary heart disease not otherwise specified (n = 58), acute myocardial infarction (MI), and certain current complications following acute MI (n = 8), cerebral infarction (n = 9), stroke, not specified as haemorrhage or infarction (n = 1), other stroke (n = 14), heart failure (n = 28), abdominal aortic aneurysm (n = 5).

For peer review only - http://bmjopen.bmj.com/site/about/guidelines.xhtml

Alternative measures	
CRP	1.21 (0.47 to 2.01)
Fibrinogen	1.22 (1.06 to 1.36)
Glucose	1.59 (1.46 to 4.59)
BMI (kg/m^2)	27 (23 to 31)
GOLD (stage)	2 (2 to 3)
4MGS (m/s)	0.95 (0.77 to 1.14)
6MWT distance (metre)	366 (255 to 440)
BODE (point)	3 (1−5)

Values are given as the median and interquartile range (IQR), or No. of cases (%). CVD = cardiovascular disease. HDL = high-density lipoprotein. SBP = systolic blood pressure. CV = cardiovascular. aPWV = carotid-femoral aortic pulse wave velocity. CIMT = carotid intima-media thickness. CRP = C-reactive protein. BMI = body mass index. GOLD = global initiative for obstructive lung disease. 4MGS = four-metre gait speed. 6MWT = six-minute walk test. BODE = BMI, obstruction (FEV_1), dyspnoea (Medical Research Council), exercise (6MWT).

Conventional cardiovascular disease risk factors

Of the conventional CVD risk factors, age, diabetes and treatment for high blood pressure had significant positive associations with the study's outcome whereas SBP had a significant negative association and other risk factors (i.e. sex, smoking status, cholesterol and HDL cholesterol) were not significantly associated with CV hospitalisation. Total cholesterol followed by diabetes and use of hypertension drug treatment contributed most to the discriminative ability of the model. The overall discriminative ability of the CV risk model had a C-statistic of 0.696, 95% CI 0.691 to 0.701; figure 1 and online supplementary table 2).

PLACE FIGURE 1 HERE

Surrogate cardiovascular risk markers

For peer review only

Neither aPWV nor CIMT were significantly associated with CV hospitalisation, after including conventional CVD risk factors (figure 2 and online supplementary table 3). Moreover, they did not significantly change the discriminative ability of the CV risk model.

PLACE FIGURE 2 HERE

Physical performance measures, COPD severity and inflammatory markers

Multivariable analysis identified that poor physical performance (i.e. reduced 6MWT distance and slower 4MGS) were significantly associated with increased CV hospitalisation, independently of conventional CVD risk factors. Severity of COPD defined by higher BODE Index (GOLD stage did not reach statistical significance: HR 1.19 (0.98 to 1.45, p=0.077), as well as inflammatory markers (i.e. CRP and fibrinogen), BMI and fasting glucose also all had significant positive associations with CV hospitalisations independently of conventional CVD risk factors.

Predictive modelling indicated significant improvement in risk discrimination when adding BMI (C = 0.705, 95% CI 0.700 to 0.711), BODE (C = 0.711, 95% CI 0.705 to 0.716), 4MGS (C = 0.713, 95% CI 0.707 to 0.718) or 6MWT (C = 0.725, 95% CI 0.720 to 0.731) to the CV risk model, but not GOLD stage, inflammatory markers or glucose (figure 3 and online supplementary table 4). Adding BMI, 4MGS, 6MWT and BODE collectively to the CV risk model resulted in a C-statistic of 0.727 (95% CI 0.721 to 0.733), indicating 6MWT primarily accounted for improved discriminative ability of the model.

PLACE FIGURE 3 HERE

All-cause and cause-specific mortality

There were 144 deaths in total (nearly 20% of the cohort), over a median follow-up period of 4.5 years. The majority of deaths were in men (n = 96, 67%) and pulmonary disease was the leading cause in

For peer review only - http://bmjopen.bmj.com/site/about/guidelines.xhtml

both sexes (table 2). Pulmonary disease accounted for 65% of deaths in women and 50% in men, followed by cancer (19% in women, 27% in men), CV (6% and 16%), and other (10% and 7% in women and men respectively).

Table 2 Mortality in the ERICA cohort.

**Cause-specific mortality**	Female No. (%)	Male No. (%)	Total No. (%)
Pulmonary	31 (65)	48 (50)	79 (55)
Cardiovascular	3 (6)	15 (16)	18 (13)
Cancer	9 (19)	26 (27)	35 (24)
Other	5 (10)	7 (7)	12 (8)
**All-cause**	48 (100)	96 (100)	144 (100)

Deaths recorded over median follow up of 4.5 years.

For peer review only

DISCUSSION

This is the first study assessing the utility of conventional CVD risk factors for CV risk prediction and the value of additional markers of risk within a COPD cohort. Novel findings include (i) poor physical performance improved the discriminative power when added to the CV risk model; (ii) BODE Index, BMI, 4MGS and systemic inflammatory markers were positively associated with CV hospitalisations independently of conventional CVD risk factors, although they did not improve the model's discriminative ability; (iii) of the conventional CVD risk factors; age, diabetes and use of anti-hypertensives were positively associated with study outcome. However, cholesterol, diabetes and use of anti-hypertensives contributed most to the model's prognostic power. Systolic blood pressure had a significant negative association with CV hospitalisations but did not add to the model's predictive ability. Furthermore, aPWV and CIMT, despite providing *in-vivo* mechanistic information about the arterial system, had no significant association with CV hospitalisations. Therefore, these data suggest that in COPD, physical performance (assessed by 6MWT) contributes to CV risk estimation defined predominantly by non-fatal CV hospitalisations. Finally, CVD contributed to only a small number of deaths at a median follow-up of 4.5 years. Pulmonary disease, followed by cancer were the major causes of death in both men and women.

We observed a C-statistic of 0.696 based on the conventional CV risk model, which increased to a maximum of 0.727 when adding additional markers; this increment was primarily due to 6MWT. These C-statistic values indicate a moderate predictive ability of the models for the study's outcome. For perspective, a C-statistic of 0.53 was observed in a Framingham model in the very elderly (aged 85-plus; the median age of our cohort was 67 years) without prior CVD, indicating it does not predict CV mortality in this group.[6] However, in primary care individuals without CVD in the Framingham study, a C-statistic of 0.76 for an outcome of general CVD (i.e. coronary heart disease, stroke, peripheral artery disease, or heart failure), demonstrated good discrimination.[10] Since our cohort included individuals with CVD at baseline, and different CVD risk factors and CV outcomes are evaluated in various studies, no direct comparison of study results can be made. That aPWV was not predictive in our cohort strikes with findings reported by Ben-Shlomo *et al.* However, from the 17 included cohorts none were COPD cohorts and the mean age of included cohorts were lower, whilst the proportion of individuals taking drugs to treat hypertension was higher.[14]

For peer review only

Overall, our study emphasises the importance of physical function as a predictor of CV risk. Tests such as 6MWT or 4MGS are proxy measures of overall mobility and physical functioning.[32] Exercise capacity and CV fitness are known to be associated with fatal- and non-fatal CVD,[33] whilst exercise-based cardiac rehabilitation reduces risk of CV events.[34] The 6MWT distance is prognostic in patients with stable coronary heart disease,[12] and those with moderate-to-severe heart failure.[35] In our cohort poor physical function improved discriminative ability of the CV risk model beyond conventional CVD factors. This has implications for clinical practice in CV risk assessment for COPD individuals, suggesting it may be helpful to incorporate physical performance into clinical assessment. Especially those aged under 65 years may benefit most from active CVD assessment, according to Morgan *et al*.[36] However, given that 6MWT can be logistically challenging to set up and time-consuming,[37] the faster and simpler 4MGS that also significantly improved the C-statistic could be used as a simpler alternative. In elderly with CVD, 4MGS is comparable to 6MWT in predicting all-cause mortality.[38]

The BODE Index also significantly improved the C-statistic of the CV risk model. However, this was primarily due to the 6MWT component. In fact, although GOLD stage had a positive association with the study outcome, this did not reach statistical significance nor improved prognostic power of the model. Therefore, despite association between airflow limitation and CV risk in general population studies,[17,39] in patients with COPD, physical performance assessment rather than other components of BODE (i.e. spirometry) adds value to CV risk prediction.

Although we did not find an association between inflammatory markers and surrogate markers of CV risk in the baseline cross-sectional component of the ERICA study,[40] both CRP and fibrinogen were associated with CV hospitalisations captured over nearly five years follow-up. That the associations remained significant after including conventional CVD risk factors indicate potential value for identifying high-risk individuals within a COPD population. The inverse relationship of SBP was an unexpected finding of borderline significance. One hypothesis is that SBP has a J-shaped curve for CV events and mortality.[41] Therefore, in this cohort, lower SBP might be a marker of sickness and frailty, hence its association with CV hospitalisations.

For peer review only

A third of the cohort had a CV related hospitalisation during follow-up and the majority had pre-existing CV comorbidity at baseline. The high numbers of CV hospitalisations, gives perspective of what this comorbidity incurs for patients and the huge healthcare costs involved. In contrast to the sizeable number of CV hospitalisations, was the small number of CV deaths. Gayle *et al.*, previously reported that CV-related mortality in chronic lung patients had already started to decline in England.[42] This may reflect better CV risk management reducing CV mortality. Importantly though, such risk management does not seem to impact CV morbidity, and is an area that requires future research to determine the optimum approach to impact CV morbidity in COPD individuals.

Nearly 20% of the cohort died during follow-up. This is comparable to the TORCH study where approximately 15% of the cohort died over 3-year follow-up.[2] Pulmonary disease, followed by cancer accounted for proportionally more deaths in our cohort compared with TORCH, whereas CV-specific mortality was less (10-15% vs 27% in TORCH). Reasons for these findings are not entirely understood but the sizeable proportion of our cohort already on medicines for dyslipidaemia and blood pressure control may be an important factor. Further studies are needed.

This study has limitations. The conventional CVD risk factors are usually used to predict CV risk defined by development of CVD. However, the majority of our participants already had CVD and were on CV medications, which may be a confounding factor impacting discriminative ability of CVD risk factors. Moreover, in our study, CV risk was defined differently (as CV hospitalisation with CV mortality). Hospitalised CV episodes were coded based on ICD-10 classifications[27] extracted from both primary and secondary positions. Although, most CV hospitalisations were recorded in secondary positions, indicating the primary admission might be related to something else. Due to the few CV events recorded in the primary position, we were unable to conduct a sensitivity analysis including CV events in the primary position only. We did not have spell data available. The study period covered the time from study enrolment until the end of study, or death. Some individuals, however, may have been admitted to hospital for CV events before study enrolment. We were unable to obtain HES data outside this period. Competing risks may have occurred, for example, individuals who died of other causes and therefore did not experience a CV event during the study period. However, our study did

For peer review only - http://bmjopen.bmj.com/site/about/guidelines.xhtml

not have sufficient statistical power to assess this. It is possible our models predict non-CV hospitalisation due to, for example, potential cross-contamination of COPD and CV hospitalisation as a result of misclassification of morbidity and mortality that often occurs when data are obtained from routine sources.[43] Deprivation scores may be another important determinant of CV risk in COPD individuals worth evaluation in future research,[44] although the relatively small cohort size of our study meant we did not include this in the analysis.

Furthermore, follow-up time for the study was a maximum of five years, whereas risk scores such as Framingham Risk Score and QRISK calculate a 10-year risk. Hence, the prognostic value of variables may alter with a different time horizon, and depends on the extent of time trends in the new biomarkers. For example, a too short time period may result in an insufficient number of events, whilst over a longer time period, e.g. 20 years, the predictive ability would diminishes because ageing is a strong predictor. We did not have access to an independent validation cohort but used cross-validation techniques instead. Generalisability of our results is limited to those with moderate COPD in the UK with NHS hospitalisations.

CONCLUSION

In a UK COPD cohort, poor physical performance assessed by 6MWT or 4MGS and inflammatory biomarkers are associated with subsequent CV-related hospitalisations, independently of conventional CVD risk factors. Importantly, poor physical performance (defined primarily by 6MWT), also significantly improved the predictive discrimination of the CV risk model. These data suggest an assessment of physical performance may enhance CV risk evaluation in individuals with COPD.

ACKNOWLEDGMENTS

The views and opinions expressed are those of the authors and do not necessarily reflect those of the University of Cambridge, the NHS, the NIHR, the Department of Health and Social Care, or other parent institutions of the authors. The corresponding author and IBW had full access to all the data in the study and takes responsibility for the integrity of the data and the accuracy of the data analysis.

For peer review only

Contributors: The following authors made substantial contributions to the conceptualisation or design: JMF, RTS, HM, MIP, AMW and IBW; data curation: JMF, IBW, KMP and AAH; formal analysis: JMF; interpretation: JMF, MF and IBW; funding acquisition: RTS, MIP and IBW; investigation: JMF, MF, CEB, WM, JRC, JF, JC, DM, KMP, AAH, MIP, CMM and IBW; methodology: JMF, AMW, IBW; project administration: JMF, RTS, MIP and IBW; resources: JMF, MF, CEB, WM, JRC, DM, MIP, CMM and IBW; software: JMF; supervision: DM, RTS, HM, MIP, AMW and IBW; validation: JMF; visualisation: JMF; and writing – original draft preparation: JMF. All authors contributed to the reviewing and editing of the manuscript.

Competing interests: All authors have completed the ICMJE uniform disclosure form at www.icmje.org/coi_disclosure.pdf and declare: GSK, a consortium partner, funded JMFs PhD. HM, RTS and DM were employees of GSK at the time this work was completed and own GSK shares and stock options. JC is employed by Cambridge University Hospitals NHS Foundation Trust and is obligated to spend 50% of his time on GSK clinical trial activity, representing a significant relationship; however, he receives no other benefits or compensation from GSK. MIP received grants from GSK outside the submitted work. IBW received grants from GSK during the conduct of the study and outside the submitted work.

Funding: This work was supported by a grant (9157-61188) from Innovate UK (formerly known as Technology Strategy Board) with contributory funding in kind (e.g. scientific expertise and meeting rooms) from GSK, a consortium partner, who also funded the corresponding author's PhD. As a consortium partner, GSK was involved in the study design, data analysis, decision to publish, and preparation of the manuscript. The specific roles of all authors are articulated in the 'author contributions' section. RTS was a co-investigator on the grant and as a consortium member was involved in the decision to publish, and preparation of the manuscript. IBW, JC and CMM acknowledge funding support from the NIHR Cambridge Comprehensive Biomedical Research Centre. CEB is supported by the NIHR Nottingham BRC respiratory theme. This work was supported by core funding from: the UK Medical Research Council (MR/L003120/1), the British Heart Foundation (RG/13/13/30194; RG/18/13/33946) and the National Institute for Health Research [Cambridge Biomedical Research Centre at the Cambridge University Hospitals NHS Foundation Trust] [*]. This

For peer review only

work was also supported by Health Data Research UK, which is funded by the UK Medical Research Council, Engineering and Physical Sciences Research Council, Economic and Social Research Council, Department of Health and Social Care (England), Chief Scientist Office of the Scottish Government Health and Social Care Directorates, Health and Social Care Research and Development Division (Welsh Government), Public Health Agency (Northern Ireland), British Heart Foundation and Wellcome. *The views expressed are those of the authors and not necessarily those of the NHS, the NIHR or the Department of Health and Social Care.

Data availability: The dataset underlying these findings, with de-identified participant data (including the data dictionary), are available to interested and qualified researchers upon request and can be obtained from the Cambridge Clinical Trials Unit. Access to hospital episode statistics requires a data sharing agreement with the National Health Services. For data access, please contact cctu@addenbrookes.nhs.uk.

Patient consent for publication: Informed patient consent was obtained in writing from all study participants and permits the publishing of all data included in this article. The investigation conforms with the principles outlined in the Declaration of Helsinki. Ethical approval was granted by the National Research Ethics Service Committee East of England - Cambridge South (11/EE/0357).

Patient and public involvement: Patients and public were not involved in the design, conduct or reporting of this study.

For peer review only

REFERENCES

1 Chen W, Thomas J, Sadatsafavi M, *et al.* Risk of cardiovascular comorbidity in patients with chronic obstructive pulmonary disease: a systematic review and meta-analysis. *Lancet Respir Med* 2015;**3**:631–9. doi:10.1016/S2213-2600(15)00241-6

2 McGarvey LP, John M, Anderson JA, *et al.* Ascertainment of cause-specific mortality in COPD: operations of the TORCH Clinical Endpoint Committee. *Thorax* 2007;**62**:411–5.

3 Garcia-Rio F, Miravitlles M, Soriano JB, *et al.* Systemic inflammation in chronic obstructive pulmonary disease: a population-based study. *Respir Res* 2010;**11**:63. doi:10.1186/1465-9921-11-63

4 Fisk M, McEniery CM, Gale N, *et al.* Surrogate Markers of Cardiovascular Risk and Chronic Obstructive Pulmonary Disease: A Large Case-Controlled Study. *Hypertension* 2018;**71**:499–506. doi:10.1161/HYPERTENSIONAHA.117.10151

5 Lozano R, Naghavi M, Foreman K, *et al.* Global and regional mortality from 235 causes of death for 20 age groups in 1990 and 2010: a systematic analysis for the Global Burden of Disease Study 2010. *Lancet* 2012;**380**:2095–128. doi:10.1016/S0140-6736(12)61728-0

6 de Ruijter W, Westendorp RG, Assendelft WJ, *et al.* Use of Framingham risk score and new biomarkers to predict cardiovascular mortality in older people: population based observational cohort study. *BMJ* 2009;**338**:a3083. doi:10.1136/bmj.a3083

7 Wannamethee SG, Shaper AG, Lennon L, *et al.* Metabolic syndrome vs Framingham Risk Score for prediction of coronary heart disease, stroke, and type 2 diabetes mellitus. *Arch Intern Med* 2005;**165**:2644–50. doi:10.1001/archinte.165.22.2644

8 Weiner DE, Tighiouart H, Elsayed EF, *et al.* The framingham predictive instrument in chronic kidney disease. *J Am Coll Cardiol* 2007;**50**:217–24. doi:10.1016/j.jacc.2007.03.037

9 Samia M, F. RR, Richey SA, *et al.* Enhanced Risk Assessment in Asymptomatic Individuals With Exercise Testing and Framingham Risk Scores. *Circulation* 2005;**112**:1566–72. doi:10.1161/CIRCULATIONAHA.105.542993

10 D'Agostino RB, Vasan RS, Pencina MJ, *et al.* General cardiovascular risk profile for use in primary care. *Circulation* 2008;**117**:743–53.

11 Lee HM, Lee J, Lee K, *et al.* Relation Between COPD Severity and Global Cardiovascular Risk in US Adults. *Chest* 2012;**142**:1118–25. doi:10.1378/chest.11-2421

For peer review only

12 Beatty AL, Schiller NB, Whooley MA. Six-minute walk test as a prognostic tool in stable coronary heart disease: data from the heart and soul study. *Arch Intern Med* 2012;**172**:1096–102. doi:10.1001/archinternmed.2012.2198

13 Emerging Risk Factors Collaboration, Kaptoge S, Di Angelantonio E, *et al.* C-reactive protein, fibrinogen, and cardiovascular disease prediction. *N Engl J Med* 2012;**367**:1310–20. doi:10.1056/NEJMoa1107477

14 Ben-Shlomo Y, Spears M, Boustred C, *et al.* Aortic pulse wave velocity improves cardiovascular event prediction: an individual participant meta-analysis of prospective observational data from 17,635 subjects. *J Am Coll Cardiol* 2014;**63**:636–46. doi:10.1016/j.jacc.2013.09.063

15 Bots ML, Groenewegen KA, Anderson TJ, *et al.* Common carotid intima-media thickness measurements do not improve cardiovascular risk prediction in individuals with elevated blood pressure: the USE-IMT collaboration. *Hypertens (Dallas, Tex 1979)* 2014;**63**:1173–81. doi:10.1161/HYPERTENSIONAHA.113.02683

16 Gan WQ, Man SFP, Senthilselvan A, *et al.* Association between chronic obstructive pulmonary disease and systemic inflammation: a systematic review and a meta-analysis. *Thorax* 2004;**59**:574–80. doi:10.1136/thx.2003.019588

17 Hole DJ, Watt GC, Davey-Smith G, *et al.* Impaired lung function and mortality risk in men and women: findings from the Renfrew and Paisley prospective population study. *BMJ* 1996;**313**:711–6. doi:10.1136/bmj.313.7059.711

18 Sciurba F, Criner GJ, Lee SM, *et al.* Six-Minute Walk Distance in Chronic Obstructive Pulmonary Disease. *Am J Respir Crit Care Med* 2003;**167**:1522–7. doi:10.1164/rccm.200203-166OC

19 Camarri B, Eastwood PR, Cecins NM, *et al.* Six minute walk distance in healthy subjects aged 55–75 years. *Respir Med* 2006;**100**:658–65. doi:10.1016/j.rmed.2005.08.003

20 Global Initiative for Chronic Obstructive Lung Disease (GOLD). Global Strategy for the Diagnosis, Management and Prevention of COPD. 2016. http://goldcopd.org/global-strategy-diagnosis-management-prevention-copd-2016/

21 Mohan D, Gale NS, McEniery CM, *et al.* Evaluating the role of inflammation in chronic airways disease: the ERICA study. *COPD* 2014;**11**:552–9. doi:10.3109/15412555.2014.898031

For peer review only

22 Wilkinson IB, Fuchs SA, Jansen IM, *et al.* Reproducibility of pulse wave velocity and augmentation index measured by pulse wave analysis. *J Hypertens* 1998;**16**:2079–84.https://www.ncbi.nlm.nih.gov/pubmed/9886900

23 Csanyi A, Egervari A. Simple clinical method of average intima-media thickness measurement in the common carotid artery. *Vasa* 1996;**25**:242–8.http://www.ncbi.nlm.nih.gov/pubmed/8795305

24 Laboratories ATSC on PS for CPF. ATS statement: guidelines for the six-minute walk test. *Am J Respir Crit Care Med* 2002;**166**:111–7. doi:10.1164/ajrccm.166.1.at1102

25 Guralnik JM, Simonsick EM, Ferrucci L, *et al.* A short physical performance battery assessing lower extremity function: association with self-reported disability and prediction of mortality and nursing home admission. *J Gerontol* 1994;**49**:M85-94.http://www.ncbi.nlm.nih.gov/pubmed/8126356

26 Celli BR, Cote CG, Marin JM, *et al.* The body-mass index, airflow obstruction, dyspnea, and exercise capacity index in chronic obstructive pulmonary disease. *N Engl J Med* 2004;**350**:1005–12. doi:10.1056/NEJMoa021322

27 Emerging Risk Factors Collaboration. The Emerging Risk Factors Collaboration: analysis of individual data on lipid, inflammatory and other markers in over 1.1 million participants in 104 prospective studies of cardiovascular diseases. *Eur J Epidemiol* 2007;**22**:839–69. doi:10.1007/s10654-007-9165-7

28 Altman DG, Vergouwe Y, Royston P, *et al.* Prognosis and prognostic research: validating a prognostic model. *BMJ* 2009;**338**:b605.

29 Newson RB. Comparing the predictive powers of survival models using Harrell's C or Somers' D. *Stata J* 2010;**10**:339–58.http://www.stata-journal.com/article.html?article=st0198

30 Smith GC, Seaman SR, Wood AM, *et al.* Correcting for optimistic prediction in small data sets. *Am J Epidemiol* 2014;**180**:318–24. doi:10.1093/aje/kwu140

31 von Elm E, Altman DG, Egger M, *et al.* The Strengthening the Reporting of Observational Studies in Epidemiology (STROBE) statement: guidelines for reporting observational studies. *J Clin Epidemiol* 2008;**61**:344–9. doi:10.1016/j.jclinepi.2007.11.008

32 Lord SR, Menz HB. Physiologic, psychologic, health predictors of 6-minute walk performance in older people. *Arch Phys Med Rehabil* 2002;**83**:907–11. doi:10.1053/apmr.2002.33227

For peer review only

33 Newman AB, Simonsick EM, Naydeck BL, *et al.* Association of long-distance corridor walk performance with mortality, cardiovascular disease, mobility limitation, and disability. *Jama-Journal Am Med Assoc* 2006;**295**:2018–26. doi:DOI 10.1001/jama.295.17.2018

34 Anderson L, Oldridge N, Thompson DR, *et al.* Exercise-Based Cardiac Rehabilitation for Coronary Heart Disease Cochrane Systematic Review and Meta-Analysis. *J Am Coll Cardiol* 2016;**67**:1–12. doi:10.1016/j.jacc.2015.10.044

35 Opasich C. Six-minute walking performance in patients with moderate-to-severe heart failure; is it a useful indicator in clinical practice? *Eur Heart J* 2001;**22**:488–96. doi:10.1053/euhj.2000.2310

36 Morgan AD, Zakeri R, Quint JK. Defining the relationship between COPD and CVD: what are the implications for clinical practice? *Ther Adv Respir Dis* 2018;**12**:1753465817750524. doi:10.1177/1753465817750524

37 Holland AE, Spruit MA, Troosters T, *et al.* An official European Respiratory Society/American Thoracic Society technical standard: field walking tests in chronic respiratory disease. *Eur Respir J* 2014;**44**:1428–46. doi:10.1183/09031936.00150314

38 Kamiya K, Hamazaki N, Matsue Y, *et al.* Gait speed has comparable prognostic capability to six-minute walk distance in older patients with cardiovascular disease. *Eur J Prev Cardiol* 2017;**25**:212–9. doi:10.1177/2047487317735715

39 D. SD, Paul MSF. Why Are Patients With Chronic Obstructive Pulmonary Disease at Increased Risk of Cardiovascular Diseases? *Circulation* 2003;**107**:1514–9. doi:10.1161/01.CIR.0000056767.69054.B3

40 Duvoix A, Dickens J, Haq I, *et al.* Blood fibrinogen as a biomarker of chronic obstructive pulmonary disease. *Thorax* 2013;**68**:670–6. doi:10.1136/thoraxjnl-2012-201871

41 SUMMIT Investigators, Brook RD, Byrd JB, *et al.* Blood pressure, heart rate, and mortality in chronic obstructive pulmonary disease: the SUMMIT trial. *Eur Heart J* 2018;**39**:3128–34. doi:10.1093/eurheartj/ehy451

42 Gayle A V, Axson EL, Bloom CI, *et al.* Changing causes of death for patients with chronic respiratory disease in England, 2005-2015. *Thorax* 2019;**74**:483 LP – 491. doi:10.1136/thoraxjnl-2018-212514

43 Macnee W, Maclay J, McAllister D. Cardiovascular injury and repair in chronic obstructive

For peer review only

pulmonary disease. *Proc Am Thorac Soc* 2008;**5**:824–33. doi:10.1513/pats.200807-071TH

44 Pujades-Rodriguez M, Timmis A, Stogiannis D, *et al.* Socioeconomic deprivation and the incidence of 12 cardiovascular diseases in 1.9 million women and men: implications for risk prediction and prevention. *PLoS One* 2014;**9**:e104671–e104671. doi:10.1371/journal.pone.0104671

For peer review only

Figure legends

Figure 1. Conventional cardiovascular disease risk factors at baseline, their hazard ratios and discriminative ability for fatal or non-fatal hospitalised cardiovascular disease.

Values are given as the median and interquartile range (IQR), or No. of cases (%). Baseline data of 714 patients are included. All models are stratified by recruitment site.

[a]Model includes age and sex.

[b]Model includes conventional cardiovascular disease risk factors: age, sex, smoking, high-density lipoprotein, total cholesterol, systolic blood pressure, diabetes, hypertension medication.

CI = confidence interval. HDL = high-density lipoprotein. SBP = systolic blood pressure. CV = cardiovascular.

There were < 5% missing values for descriptive variables such as BMI and smoking status. Missing values were addressed using multiple imputations using chained equations.

Figure 2. Aortic stiffness at baseline, their hazard ratios and discriminative ability for fatal or non-fatal hospitalised cardiovascular disease.

Values are given as the median and interquartile range (IQR), or No. of cases (%). Baseline data of 714 patients are included. All models are stratified by recruitment site.

Goodness of fit (chi2(3), P > chi2): CV risk model (4.30, 0.231), aPWV (2.81, 0.422), CIMT (1.57, 0.666), AIx (0.99, 0.803). Estimates based on quartiles of risk.

Brier score (95% CI): CV risk model 0.122 (0.106 to 0.138), aPWV 0.124 (0.107 to 0.142), CIMT 0.122 (0.105 to 0.139), AIx 0.123 (0.106 to 0.139). Lower score indicates better accuracy of estimates.

[a]Model includes age and sex.

[b]Model includes conventional cardiovascular disease risk factors: age, sex, smoking, high-density lipoprotein, total cholesterol, systolic blood pressure, diabetes, hypertension medication. Carotid-femoral aortic pulse wave velocity further included mean arterial pressure and resting heart rate. AIx further included resting heart rate and height.

CI = confidence interval. PWV = pulse wave velocity. CIMT = carotid intima-media thickness. AIx = augmentation index.

There were about 10% missing values for variables CIMT (n = 66) and aPWV (n = 60). Missing values were addressed using multiple imputations using chained equations.

For peer review only

Figure 3. Alternative measures at baseline, their hazard ratios and discriminative ability for fatal or non-fatal hospitalised cardiovascular disease.

Values are given as the median and interquartile range (IQR), or No. of cases (%). Baseline data of 714 patients are included. All models are stratified by recruitment site.

Goodness of fit (chi2(3), P > chi2): CV risk model (4.30, 0.231), CRP (4.26, 0.235), Fibrinogen (1.89, 0.595), Glucose (0.87, 0.832), BMI (1.03, 0.794), GOLD (1.75, 0.625), 4MGS (6.69, 0.083), 6MW (7.28, 0.063), BODE (1.88, 0.599), BMI, 4MGS, 6MW, BODE (5.28, 0.152). Estimates based on quartiles of risk.

Brier score (95% CI): CV risk model 0.122 (0.106 to 0.138), CRP 0.121 (0.104 to 0.137), Fibrinogen 0.122, (0.106 to 0.139), Glucose 0.125 (0.108 to 0.141), BMI 0.122 (0.105 to 0.138) GOLD 0.123 (0.107 to 0.139), 4MGS 0.122 (0.105 to 0.138), 6MW 0.112 (0.101 to 0.133), BODE 0.119 (0.102 to 0.136), BMI, 4MGS, 6MW, BODE 0.116 (0.100 to 0.133). Lower score indicates better accuracy of estimates.

[a]Model includes age and sex.

[b]Model includes conventional cardiovascular disease risk factors: age, sex, smoking, high-density lipoprotein, total cholesterol, systolic blood pressure, diabetes, hypertension medication.

CI = confidence interval. CRP = C-reactive protein. BMI = body mass index. GOLD = global initiative for chronic obstructive lung disease. 4MGS = four-metre gait speed. 6MWT = six-minute walk test. BODE = body mass index, obstruction, dyspnoea, exercise.

There were < 5% missing values for biochemical markers including fibrinogen and cholesterol. Missing values were addressed using multiple imputations using chained equations.

For peer review only

Online supplementary material

Supplementary text 1 Supplementary methods.

Supplementary table 1 Definitions of diagnoses by ICD-10 coding.

Supplementary table 2 Fatal or non-fatal hospitalised cardiovascular disease by ICD-10 coding position.

Supplementary table 3 Conventional cardiovascular disease risk factors at baseline, their hazard ratios for cardiovascular disease.

Supplementary table 4 Arterial stiffness at baseline, their hazard ratios for cardiovascular disease.

Supplementary table 5 Alternative measures at baseline, their hazard ratios for cardiovascular disease.

Supplementary figure 1 Percentage of missing values.

Supplementary figure 2 Percentage and pattern of missing values in key baseline characteristics.

Supplementary figure 3 Differences in baseline pulse wave velocity.

Supplementary figure 4 Differences in baseline carotid intima-media thickness.

Supplementary figure 5 Differences in baseline augmentation index.

Supplementary figure 6 Participant enrolment flow diagram with five years follow up.

For peer review only

Risk factor	Median (IQR) or n (%)	HR (95% CI)[a]	HR (95% CI)[b]	C−statistic
All conventional CVD risk factors				0.696 [0.691 to 0.701]
Age − per 10 year increase	67 (62−73)	1.50 (1.26 to 1.78)	1.43 (1.18 to 1.72)	0.594 [0.588 to 0.602]
Sex − male	434 (61)	1.16 (0.88 to 1.52)	1.12 (0.84 tot 1.49)	0.551 [0.542 to 0.558]
Smoking − current	218 (31)	0.93 (0.70 to 1.25)	0.91 (0.68 to 1.23)	0.537 [0.528 to 0.544]
HDL − per 1 mmol/L increase	1.4 (1.2−1.7)	0.61 (0.44 to 0.84)	0.91 (0.66 to 1.27)	0.569 [0.563 to 0.577]
Cholesterol − per 1 mmol/L increase	5.0 (4.3−5.8)	0.72 (0.63 to 0.81)	0.88 (0.76 to 1.01)	0.618 [0.611 to 0.623]
SBP − per 10 mmHg increase	142 (131−154)	0.92 (0.85 to 0.99)	0.93 (0.86 to 1.00)	0.540 [0.532 to 0.547]
Diabetes − yes	82 (12)	4.16 (3.09 to 5.60)	3.23 (2.32 to 4.50)	0.616 [0.607 to 0.622]
Hypertension medication − yes	245 (34)	1.77 (1.37 to 2.29)	1.50 (1.15 to 1.96)	0.612 [0.604 to 0.616]

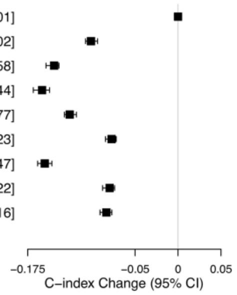

Conventional cardiovascular disease risk factors at baseline, their hazard ratios and discriminative ability for fatal or non-fatal hospitalised cardiovascular disease.

Values are given as the median and interquartile range (IQR), or No. of cases (%). Baseline data of 714 patients are included. All models are stratified by recruitment site.

[a]Model includes age and sex.

[b]Model includes conventional cardiovascular disease risk factors: age, sex, smoking, high-density lipoprotein, total cholesterol, systolic blood pressure, diabetes, hypertension medication.

CI = confidence interval. HDL = high-density lipoprotein. SBP = systolic blood pressure. CV = cardiovascular.

There were < 5% missing values for descriptive variables such as BMI and smoking status. Missing values were addressed using multiple imputations using chained equations.

309x95mm (300 x 300 DPI)

Risk factor	Median (IQR) or n (%)	HR (95% CI)[a]	HR (95% CI)[b]	C−statistic
All conventional CVD risk factors				0.696 [0.691 to 0.701]
+ PWV – per 1 m/sec increase	9.8 (8.4–11.8)	1.04 (0.98 to 1.10)	0.99 (0.93 to 1.06)	0.695 [0.690 to 0.700]
+ CIMT – per 1 mm increase	0.81 (0.71–0.96)	1.18 (0.57 to 2.44)	1.29 (0.60 to 2.77)	0.694 [0.689 to 0.700]
+ AIx – per 5% increase	28 (20–34)	0.84 (0.78 to 0.92)	0.92 (0.84 to 1.01)	0.695 [0.690 to 0.700]

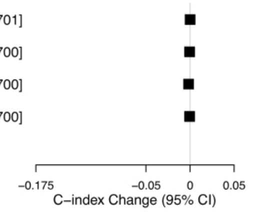

Aortic stiffness at baseline, their hazard ratios and discriminative ability for fatal or non-fatal hospitalised cardiovascular disease.
Values are given as the median and interquartile range (IQR), or No. of cases (%). Baseline data of 714 patients are included. All models are stratified by recruitment site.
Goodness of fit (chi2(3), P > chi2): CV risk model (4.30, 0.231), aPWV (2.81, 0.422), CIMT (1.57, 0.666), AIx (0.99, 0.803). Estimates based on quartiles of risk.
Brier score (95% CI): CV risk model 0.122 (0.106 to 0.138), aPWV 0.124 (0.107 to 0.142), CIMT 0.122 (0.105 to 0.139), AIx 0.123 (0.106 to 0.139). Lower score indicates better accuracy of estimates.

[a]Model includes age and sex.

[b]Model includes conventional cardiovascular disease risk factors: age, sex, smoking, high-density lipoprotein, total cholesterol, systolic blood pressure, diabetes, hypertension medication. Carotid-femoral aortic pulse wave velocity further included mean arterial pressure and resting heart rate. AIx further included resting heart rate and height.
CI = confidence interval. PWV = pulse wave velocity. CIMT = carotid intima-media thickness. AIx = augmentation index.
There were about 10% missing values for variables CIMT (n = 66) and aPWV (n = 60). Missing values were addressed using multiple imputations using chained equations.

302x69mm (300 x 300 DPI)

Risk factor	Median (IQR) or n (%)	HR (95% CI)[a]	HR (95% CI)[b]	C−statistic
All conventional CVD risk factors				0.696 [0.691 to 0.701]
+ CRP – per twofold increase	1.21 (0.47 to 2.01)	1.20 (1.07 to 1.35)	1.17 (1.04 to 1.31)	0.698 [0.693 to 0.703]
+ Fibrinogen – per twofold increase	1.22 (1.06 to 1.36)	1.75 (1.14 to 2.67)	1.75 (1.14 to 2.68)	0.695 [0.690 to 0.700]
+ Glucose – per twofold increase	1.59 (1.46 to 4.59)	2.55 (1.43 to 4.53)	1.94 (1.05 to 3.56)	0.695 [0.690 to 0.700]
+ BMI – per 1 kg/m^2 increase	27 (23–31)	1.07 (1.04 to 1.09)	1.04 (1.02 to 1.07)	0.705 [0.700 to 0.711]
+ GOLD – per 1 stage increase	1 (1–2)	1.19 (0.98 to 1.45)	1.28 (1.04 to 1.57)	0.697 [0.691 to 0.701]
+ 4MGS – per 1 second increase	4.2 (3.5 to 5.2)	1.10 (1.05 to 1.16)	1.08 (1.02 to 1.15)	0.713 [0.707 to 0.718]
+ 6MW distance – per 30 meter increase	366 (255 to 440)	0.91 (0.88 to 0.93)	0.91 (0.88 to 0.94)	0.725 [0.720 to 0.731]
+ BODE – per 1 point increase	3 (1–5)	1.13 (1.07 to 1.18)	1.14 (1.08 to 1.20)	0.711 [0.705 to 0.716]
+ BMI, 4MGS, 6MW, BODE – per 1 unit increase				0.727 [0.721 to 0.733]

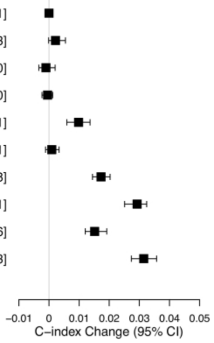

Alternative measures at baseline, their hazard ratios and discriminative ability for fatal or non-fatal hospitalised cardiovascular disease.
Values are given as the median and interquartile range (IQR), or No. of cases (%). Baseline data of 714 patients are included. All models are stratified by recruitment site.
Goodness of fit (chi2(3), P > chi2): CV risk model (4.30, 0.231), CRP (4.26, 0.235), Fibrinogen (1.89, 0.595), Glucose (0.87, 0.832), BMI (1.03, 0.794), GOLD (1.75, 0.625), 4MGS (6.69, 0.083), 6MW (7.28, 0.063), BODE (1.88, 0.599), BMI, 4MGS, 6MW, BODE (5.28, 0.152). Estimates based on quartiles of risk.
Brier score (95% CI): CV risk model 0.122 (0.106 to 0.138), CRP 0.121 (0.104 to 0.137), Fibrinogen 0.122, (0.106 to 0.139), Glucose 0.125 (0.108 to 0.141), BMI 0.122 (0.105 to 0.138) GOLD 0.123 (0.107 to 0.139), 4MGS 0.122 (0.105 to 0.138), 6MW 0.112 (0.101 to 0.133), BODE 0.119 (0.102 to 0.136), BMI, 4MGS, 6MW, BODE 0.116 (0.100 to 0.133). Lower score indicates better accuracy of estimates.

[a]Model includes age and sex.

[b]Model includes conventional cardiovascular disease risk factors: age, sex, smoking, high-density lipoprotein, total cholesterol, systolic blood pressure, diabetes, hypertension medication.
CI = confidence interval. CRP = C-reactive protein. BMI = body mass index. GOLD = global initiative for chronic obstructive lung disease. 4MGS = four-metre gait speed. 6MWT = six-minute walk test. BODE = body mass index, obstruction, dyspnoea, exercise.
There were < 5% missing values for biochemical markers including fibrinogen and cholesterol. Missing values were addressed using multiple imputations using chained equations.

331x119mm (300 x 300 DPI)

Supplementary materials for "Cardiovascular risk prediction using physical performance measures in COPD: results from a multi-centre observational study."

Jilles M. Fermont, Marie Fisk, Charlotte E. Bolton, William Macnee, John Cockcroft, Jonathan Fuld, Joseph Cheriyan, Divya Mohan, Kaisa M. Mäki-Petäjä, Ali B.A.K. Al-Hadithi, Ruth Tal-Singer, Hana Müllerova, Michael I. Polkey, Angela M. Wood, Carmel McEniery, Ian B. Wilkinson, on behalf of the ERICA consortium.

For peer review only

Supplementary text

Text 1 Supplementary methods

There were missing values. Data were assessed for the level and type of missing data, and completion patterns (Figures 1-2). There were about 10% missing values for variables CIMT (n = 66) and PWV (n = 60), with <5% missing values for other variables (Figures 3-5). Missing values were addressed using multiple imputations using chained equations (MICE). The time-to-event outcome was included using the non-parametric Nelson-Aalen estimator. Predictive mean matching was used for continuous variables, ordered logistic regression (as continuous) for ordinal variables, multinomial logistic regression for categorical variables, and logistic regression for binary variables. Derived variables such as the BODE Index (a composite score of BMI, forced expiratory volume in one second (FEV_1), Medical Research Council dyspnoea score, and 6MWT distance) and GOLD stage were estimated post MICE using passive imputation.

Data were cleaned for episode status, and inpatient (i.e. hospitalised) CV episodes were identified based on classifications used by the Emerging Risk Factors Collaboration.[1] Cardiovascular events were extracted from both primary and secondary positions of ICD-10. Priority was given to CV events reported in the primary position (n = 43). Following this, we selected CV events in the secondary position (n = 189), which had no recording of a CV event in the primary position. Only episodes during the study follow-up were evaluated.

For peer review only

[1] The Emerging Risk Factors Collaboration: analysis of individual data on lipid, inflammatory and other markers in over 1.1 million participants in 104 prospective studies of cardiovascular diseases. *Eur J Epidemiol* **22,** 839–869 (2007). https://doi.org/10.1007/s10654-007-9165-7

Supplementary tables

Table 1 Definitions of diagnoses by ICD-10 coding.

End point	ICD-10 codes
**All cardiovascular disease**	**E10.5, E11-E14, F01, G46.3-G46.7, G458, G459<br>I11.0, I13.0, I13.2, I20.0-I20.1, I20.8-I25, I50, I60, I61, I.62, I63, I64, I65-I69, I70.2, I71.3-I71.9, I72, I73.9-I79, R96 + cardiac death**
**Diseases of the arteries**	**I70.2, I72, I73.9-I79, E10.5, E11-E14**
Peripheral arterial disease	I70.2, I73.9, E10.5, E11-E14
Diseases of arteries, arterioles and capillaries	I72, I74-I79
**Coronary heart disease**	**I20.0-I20.1, I20.8-I25**
Angina	I20.1, I20.8-I20.9
Unstable angina	I20.0, I24
Coronary heart disease not otherwise specified	I25
Acute myocardial infarction (MI), and certain current complications following acute MI	I21, I23
Subsequent myocardial infarction	I22
**All stroke**	**I60, I61, I.62, I63, I64, I65-I69, F01, G46.3-G46.7, G458, G459**
Subarachnoid haemorrhage	I60
Intra-cerebral haemorrhage	I61
Cerebral infarction	I63
Stroke, not specified as haemorrhage or infarction	I64
Stroke syndromes	G46.3-G46.7
Transient ischemic attack	G458, G459
Other stroke	I62, I65-I69, F01
**Heart failure**	**I11.0, I13.0, I13.2, I50**
Heart failure	I50
Hypertensive heart disease with (congestive) heart failure	I11.0
Hypertensive heart and renal disease with (congestive) heart failure	I13.0
Hypertensive heart and renal disease with both (congestive) heart failure and renal disease	I13.2
**Cardiac death**	**Adjudicated**
**Other vascular deaths**	**R96, I71.3-I71.9**
Sudden death, cause unknown	R96
Abdominal aortic aneurysm	I71.3-I71.9

For peer review only

Table 2 Fatal or non-fatal hospitalised cardiovascular disease by ICD-10 coding position.

	All cardiovascular disease			AECOPD	
	**Primary ICD-10 position**	**Secondary ICD-10 position**	**Cardiac death**	**Primary ICD-10 position**	**Secondary ICD-10 position**
1	I702			J440	
2	I660			J440	
3	I64X			J440	
4	I638			J441	
5	I500			J440	
6	I251			J441	
7	I251			J441	
8	I251			J440	
9	I251			J440	
10	I251			J440	
11	I251			J440	
12	I251			J440	
13	I219			J440	
14	I209			J441	
15		E119		J441	
16		E119		J440	
17		E119		J440	
18		E119		J441	
19		E119		J441	
20		E119		J441	
21		E119		J441	
22		E119		J440	
23		E119		J440	
24		E119		J440	
25		E119		J449	
26		E119		J440	
27		E119		J440	
28		E119		J441	
29		E119		J440	
30		E119		J441	
31		E119		J440	
32		E119		J440	
33		E119		J440	
34		E119		J441	
35		E119		J441	
36		E119		J440	
37		E119		J440	
38		E119		J439	
39		E119		J449	
40		E119		J440	
41		E149		J440	
42		I209		J440	
43		I209		J441	
44		I209		J440	
45		I209		J441	
46		I209		J440	
47		I209		J440	
48		I209		J440	
49		I219		J440	
50		I219		J440	
51		I219		J440	

For peer review only - http://bmjopen.bmj.com/site/about/guidelines.xhtml

52		I219		J441	
53		I251		J440	
54		I251		J441	
55		I252		J441	
56		I252		J440	
57		I252		J441	
58		I252		J440	
59		I258		J441	
60		I258		J440	
61		I258		J440	
62		I259		J440	
63		I259		J441	
64		I259		J441	
65		I259		J441	
66		I259		J440	
67		I259		J440	
68		I259		J441	
69		I259		J440	
70		I500		J441	
71		I500		J440	
72		I500		J441	
73		I500		J440	
74		I500		J440	
75		I501		J440	
76		I501		J440	
77		I501		J441	
78		I501		J440	
79		I509		J440	
80		I509		J440	
81		I634		J438	
82		I652		J440	
83		I671		J441	
84		I678		J440	
85		I678		J440	
86		I678		J440	
87		I678		J440	
88		I714		J441	
89		I714		J449	
90		I739		J440	
91		I798		J440	
92	I771				
93	I771				
94	I745				
95	I743				
96	I739				
97	I739				
98	I739				
99	I739				J440
100	I652				
101	I652				
102	I639				J448
103	I639				J440
104	I639				
105	I639				
106	I638				
107	I634				J440
108	I632				
109	I509				

For peer review only

110	I509				
111	I501				
112	I500				
113	I251				
114	I251				
115	I251				
116	I251				
117	I251				J440
118	I219				
119	I209				
120	I200				J440
121		E112			
122		E119			
123		E119			
124		E119			J440
125		E119			
126		E119			J440
127		E119			J448
128		E119			J440
129		E119			
130		E119			
131		E119			
132		E119			
133		E119			
134		E119			J440
135		E119			
136		E119			
137		E119			J440
138		E119			
139		E119			
140		E119			J440
141		E119			J440
142		E119			
143		E119			
144		E119			
145		E119			
146		E119			
147		E119			
148		E119			J440
149		E119			J440
150		E119			
151		E119			
152		E119			
153		E119			
154		E119			
155		E119			
156		E119			
157		E119			
158		E119			
159		E119			
160		E119			
161		E119			J440
162		E119			J440
163		E119			J440
164		E149			
165		E149			
166		I200			
167		I209			

For peer review only

168		I209			
169		I209			J440
170		I209			
171		I209			
172		I209			
173		I209			
174		I209			
175		I209			
176		I209			
177		I209			J440
178		I209			
179		I211			
180		I251			
181		I251			
182		I251			J440
183		I252			J440
184		I252			
185		I252			J440
186		I252			
187		I252			
188		I252			
189		I252			J440
190		I252			
191		I252			J440
192		I252			
193		I252			
194		I252			
195		I252			
196		I259			
197		I259			
198		I259			J440
199		I259			J440
200		I259			J440
201		I259			
202		I259			J440
203		I259			J440
204		I259			J440
205		I259			
206		I259			J440
207		I259			
208		I259			J448
209		I259			
210		I259			J440
211		I500			J440
212		I500			J448
213		I500			J440
214		I501			
215		I501			J440
216		I501			J440
217		I501			J440
218		I509			
219		I509			J440
220		I652			
221		I671			
222		I678			J440
223		I678			
224		I679			
225		I679			

For peer review only

226		I694			
227		I713			J440
228		I714			
229		I714			J440
230		I739			
231		I739			J440
232		I780			J440
233			Cardiac		
234			Cardiac		
235			Cardiac		
236			Cardiac		
237			Cardiac		
Total recorded	43	189	5	91	47

Fourteen individuals with a CV event recorded in the primary position also had an acute exacerbation of COPD (AECOPD) recorded in the primary position during the study period. In addition, 77 individuals with a CV event recorded in the secondary position also had an AECOPD recorded in the primary position during the study period.

For peer review only

Table 3 Conventional cardiovascular disease risk factors at baseline, their hazard ratios for cardiovascular disease.

	Median (IQR) or No. (%)	HR (95% CI)[a]	P value	HR (95% CI)[b]	P value
**Conventional CVD risk factors**					
Age – per 10 year increase	67 (62-73)	1.50 (1.26 to 1.78)	< 0.001	1.43 (1.18 to 1.72)	< 0.001
Sex – males	434 (61)	1.16 (0.88 to 1.52)	0.282	1.12 (0.84 tot 1.49)	0.437
Smoking – current	218 (31)	0.93 (0.70 to 1.25)	0.650	0.91 (0.68 to 1.23)	0.554
HDL – per 1 mmol/L increase	1.4 (1.2-1.7)	0.61 (0.44 to 0.84)	0.003	0.91 (0.66 to 1.27)	0.596
Total cholesterol – per 1 mmol/L increase	5.0 (4.3-5.8)	0.72 (0.63 to 0.81)	< 0.001	0.88 (0.76 to 1.01)	0.063
SBP – per 10 mmHg increase	142 (131-154)	0.92 (0.85 to 0.99)	0.029	0.93 (0.86 to 1.00)	0.061
Diabetes – yes	82 (12)	4.16 (3.09 to 5.60)	< 0.001	3.23 (2.32 to 4.50)	< 0.001
Hypertension treatment – yes	245 (34)	1.77 (1.37 to 2.29)	< 0.001	1.50 (1.15 to 1.96)	0.003

Values are given as the median and interquartile range (IQR), or No. of cases (%). Baseline data of 714 patients are included. All models are stratified by recruitment site.
[a]Model includes age and sex.
[b]Model includes conventional cardiovascular disease risk factors: age, sex, smoking, high-density lipoprotein, total cholesterol, systolic blood pressure, diabetes, hypertension drug treatment.
CI = confidence interval. HDL = high-density lipoprotein. SBP = systolic blood pressure. CV = cardiovascular.

For peer review only

Table 4 Arterial stiffness at baseline, their hazard ratios for cardiovascular disease.

	Median (IQR) or No. (%)	HR (95% CI)[a]	P value	HR (95% CI)[b]	P value
**Measures of arterial stiffness**					
PWV – per 1 m/sec increase	9.8 (8.4-11.8)	1.04 (0.98 to 1.10)	0.159	0.99 (0.93 to 1.06)	0.839
CIMT – per 1 mm increase	0.81 (0.71-0.96)	1.18 (0.57 to 2.44)	0.651	1.29 (0.60 to 2.77)	0.519
AIx – per 5% increase	28 (20-34)	0.84 (0.78 to 0.92)	< 0.001	0.92 (0.84 to 1.01)	0.056

Values are given as the median and interquartile range (IQR), or No. of cases (%). Baseline data of 714 patients are included. All models are stratified by recruitment site.
[a]Model includes age and sex.
[b]Model includes conventional cardiovascular disease risk factors: age, sex, smoking, high-density lipoprotein, total cholesterol, systolic blood pressure, diabetes, hypertension drug treatment. PWV further included mean arterial pressure and resting heart rate. AIx further included resting heart rate and height.
CI = confidence interval. PWV = pulse wave velocity. CIMT = carotid intima-media thickness. AIx = augmentation index.

For peer review only

Table 5 Alternative measures at baseline, their hazard ratios for cardiovascular disease.

	Median (IQR) or No. (%)	HR (95% CI)[a]	P value	HR (95% CI)[b]	P value
**Other risk factors**					
CRP – per twofold increase	1.21 (0.47 to 2.01)	1.20 (1.07 to 1.35)	0.002	1.17 (1.04 to 1.31)	0.010
Fibrinogen – per twofold increase	1.22 (1.06 to 1.36)	1.75 (1.14 to 2.67)	0.009	1.75 (1.14 to 2.68)	0.010
Glucose – per twofold increase	1.59 (1.50 to 1.69)	2.55 (1.43 to 4.53)	0.001	1.94 (1.05 to 3.56)	0.033
BMI – per 1 kg/m^2 increase	27 (23-31)	1.07 (1.04 to 1.09)	< 0.001	1.04 (1.02 to 1.07)	0.001
GOLD – per 1 stage increase	1 (1-2)	1.19 (0.98 to 1.45)	0.077	1.28 (1.04 to 1.57)	0.018
4MGST – per 1 second increase	4.2 (3.5 to 5.2)	1.10 (1.05 to 1.16)	< 0.001	1.08 (1.02 to 1.15)	0.010
6MWT distance – per 30 metre increase	366 (255 to 440)	0.91 (0.88 to 0.93)	< 0.001	0.91 (0.88 to 0.94)	< 0.001
BODE – per 1 point increase	3 (1-5)	1.13 (1.07 to 1.18)	< 0.001	1.14 (1.08 to 1.20)	< 0.001

Values are given as the median and interquartile range (IQR), or No. of cases (%). Baseline data of 714 patients are included. All models are stratified by recruitment site.
[a]Model includes age and sex.
[b]Model includes conventional cardiovascular disease risk factors: age, sex, smoking, high-density lipoprotein, total cholesterol, systolic blood pressure, diabetes, hypertension drug treatment.
CI = confidence interval. CRP = C-reactive protein. BMI = body mass index. GOLD = global initiative for chronic obstructive lung disease. 4MGS = four-metre gait speed. 6MWT = six-minute walk test. BODE = body mass index, obstruction, dyspnoea, exercise.

For peer review only

Supplementary figures

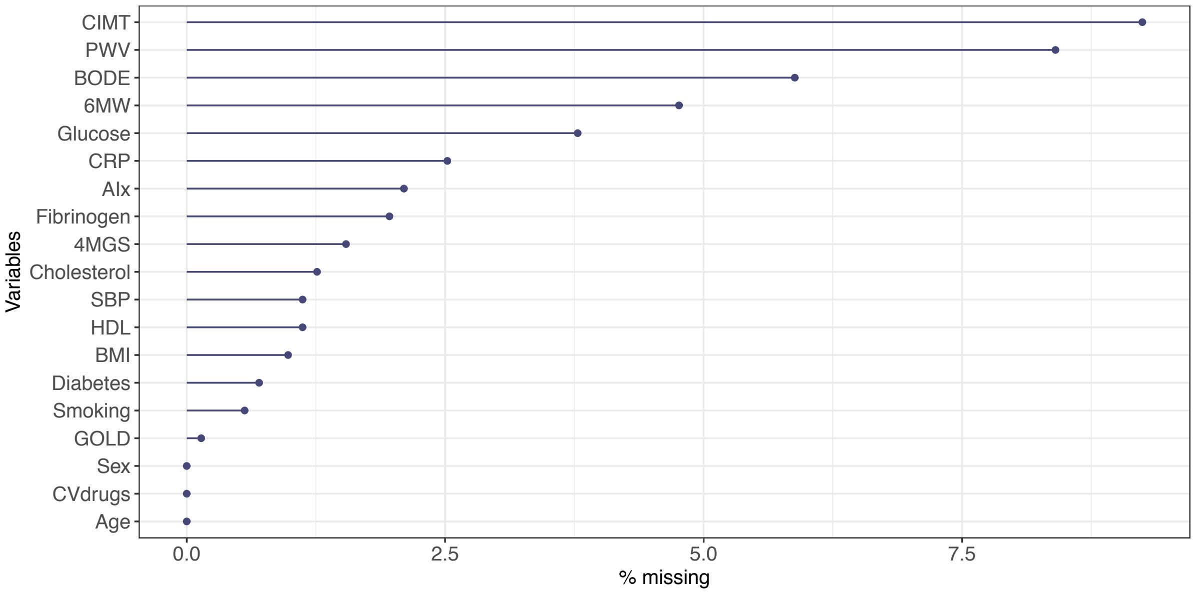

Figure 1 Percentage of missing values.
CIMT = carotid intima-media thickness. PWV = pulse wave velocity. BODE = body mass index, obstruction (i.e. forced expiratory volume in one second), dyspnoea score, exercise (i.e. six-minute walk test distance). 6MWT = six-minute walk test. CRP = C-reactive protein. AIx = augmentation index. 4MGS = four-metre gait speed. SBP = systolic blood pressure. HDL = high-density lipoprotein. BMI = body mass index. GOLD = global initiative for obstructive lung disease. CV = cardiovascular.

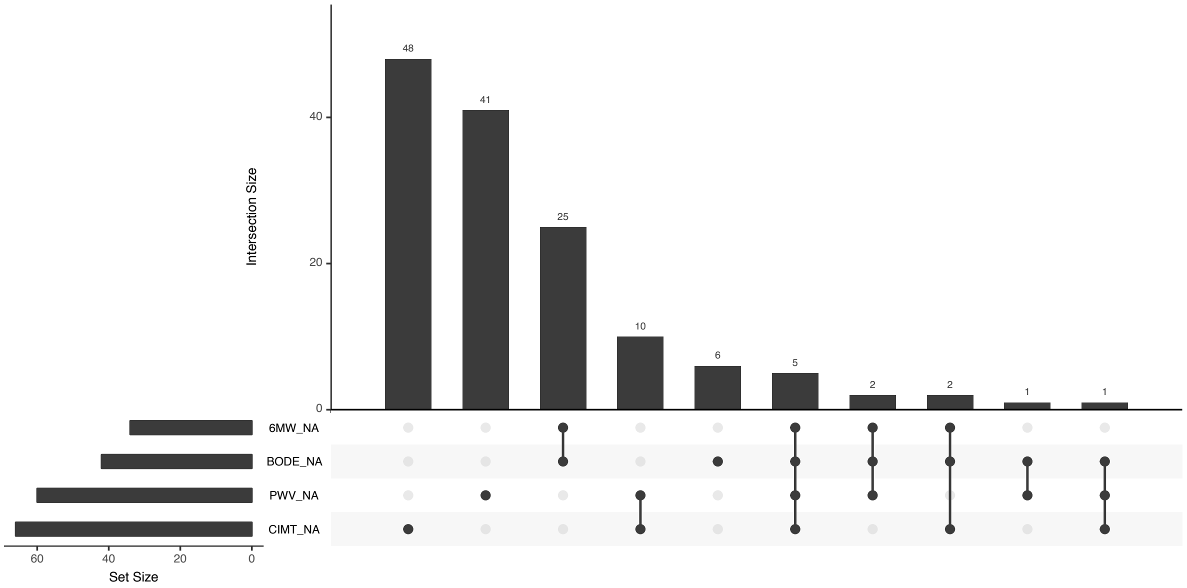

Figure 2 Percentage and pattern of missing values in key baseline characteristics.
6MWT, six-minute walk test. BODE = body mass index, obstruction (i.e. forced expiratory volume in one second), dyspnoea score, exercise (i.e. six-minute walk test distance). PWV, pulse wave velocity. CIMT, carotid-intima media thickness.

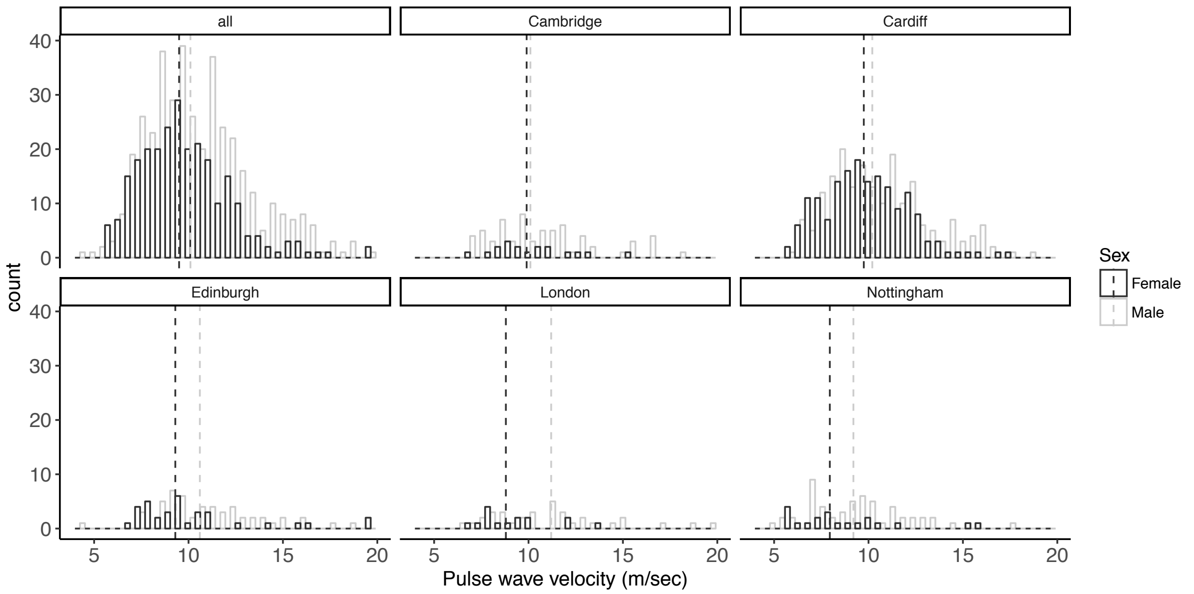

Figure 3 Differences in baseline pulse wave velocity.
Histograms displaying the distribution of PWV by sex and recruitment site. Dashed lines indicate median values by sex. Where only one median line is visible, medians for both sexes are similar.

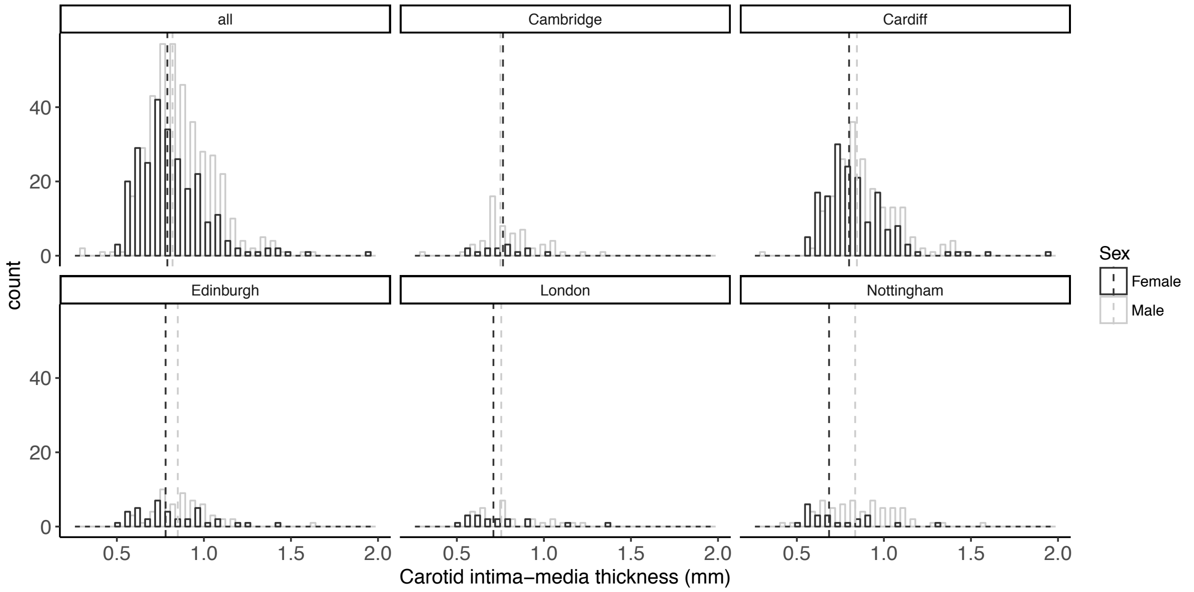

Figure 4 Differences in baseline carotid intima-media thickness.
Histograms displaying the distribution of CIMT by sex and recruitment site. Dashed lines indicate median values by sex. Where only one median line is visible, medians for both sexes are similar.

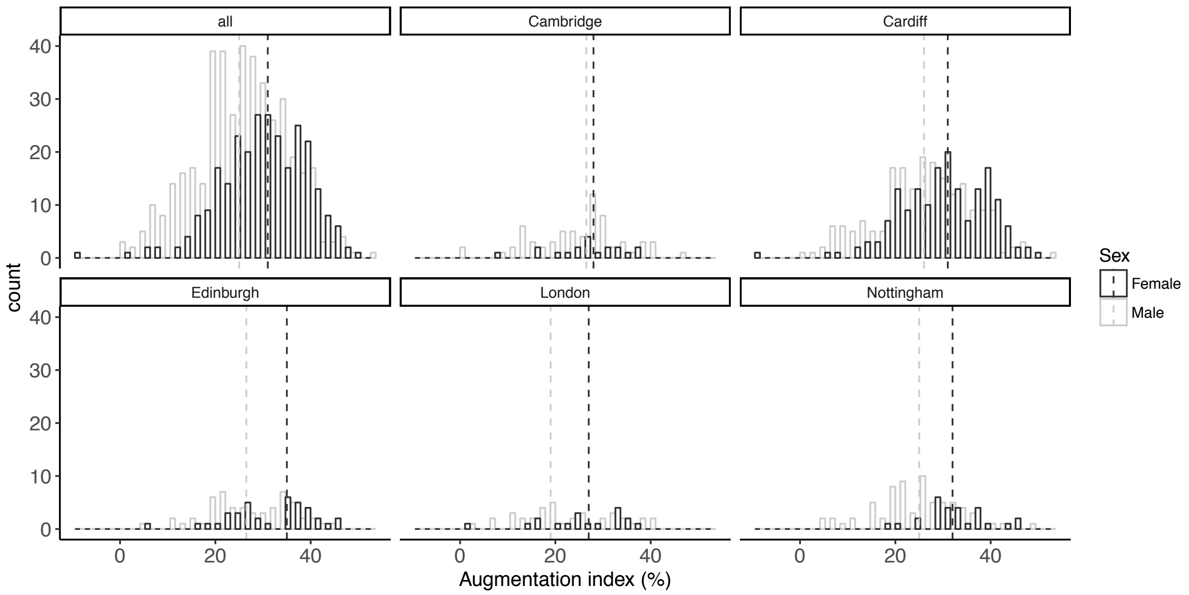

Figure 5 Differences in baseline augmentation index.
Histograms displaying the distribution of AIx by sex and recruitment site. Dashed lines indicate median values by sex.

For peer review only

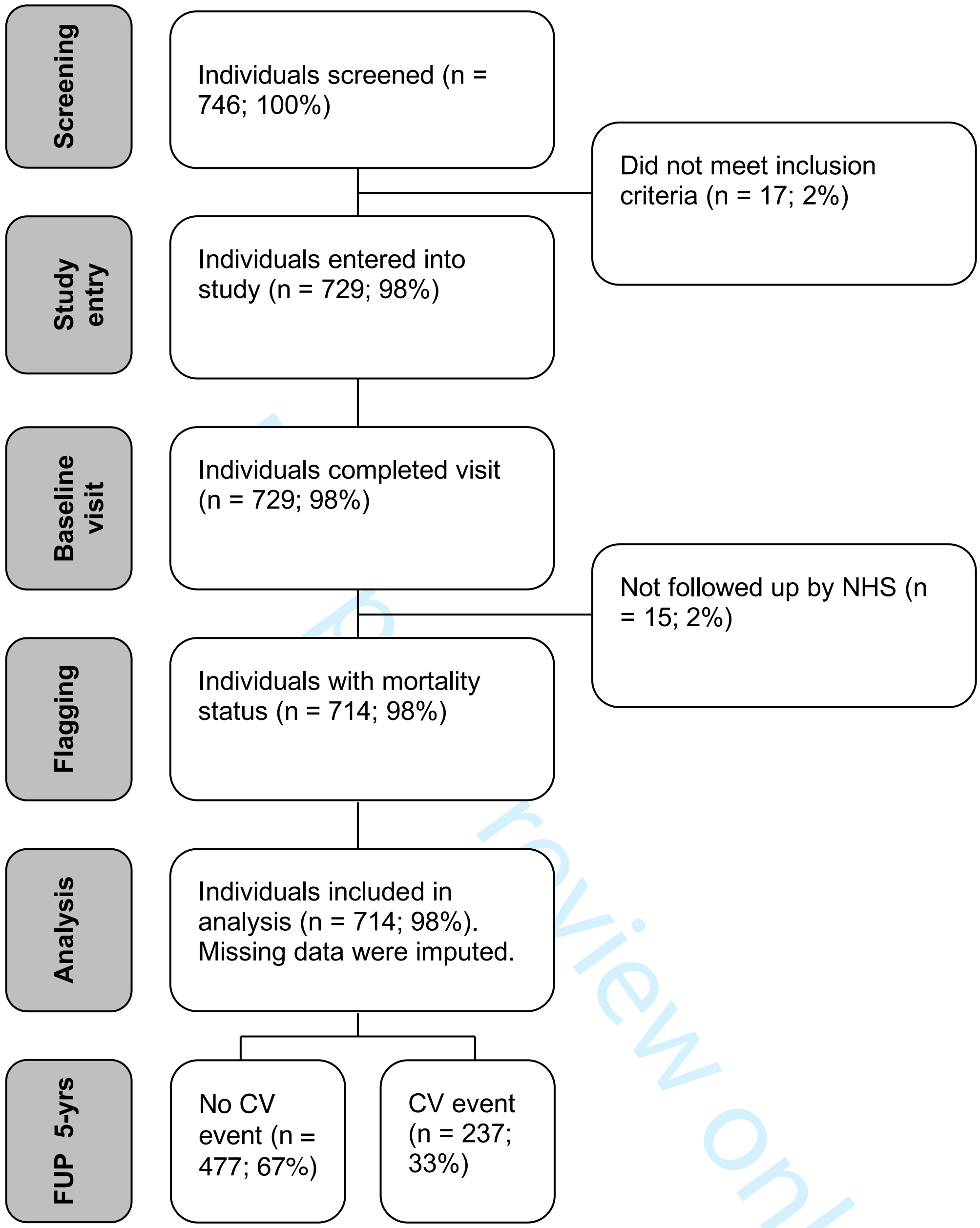

Figure 6 Participant enrolment flow diagram with five years follow up.
Total number of CV events (n = 237): peripheral arterial disease (n = 77), diseases of arteries, arterioles and capillaries (n = 6), angina (n = 24), unstable angina (n = 2), coronary heart disease not otherwise specified (n = 58), acute myocardial infarction (MI), and certain current complications following acute MI (n = 8), cerebral infarction (n = 9), stroke, not specified as haemorrhage or infarction (n = 1), other stroke (n = 14), heart failure (n = 28), and abdominal aortic aneurysm (n = 5). FEV_1 = forced expiratory volume one second. FVC = forced vital capacity. NHS = National Health Services. FUP = follow-up period.

STROBE 2007 (v4) Statement—Checklist of items that should be included in reports of *cohort studies*

**Section/Topic**	**Item #**	**Recommendation**	**Reported on page #**
**Title and abstract**	1	(*a*) Indicate the study's design with a commonly used term in the title or the abstract	1
		(*b*) Provide in the abstract an informative and balanced summary of what was done and what was found	3
**Introduction**			
Background/rationale	2	Explain the scientific background and rationale for the investigation being reported	6
Objectives	3	State specific objectives, including any prespecified hypotheses	7
**Methods**			
Study design	4	Present key elements of study design early in the paper	8
Setting	5	Describe the setting, locations, and relevant dates, including periods of recruitment, exposure, follow-up, and data collection	8
Participants	6	(*a*) Give the eligibility criteria, and the sources and methods of selection of participants. Describe methods of follow-up	8
		(*b*) For matched studies, give matching criteria and number of exposed and unexposed	
Variables	7	Clearly define all outcomes, exposures, predictors, potential confounders, and effect modifiers. Give diagnostic criteria, if applicable	8-9
Data sources/ measurement	8*	For each variable of interest, give sources of data and details of methods of assessment (measurement). Describe comparability of assessment methods if there is more than one group	8-9
Bias	9	Describe any efforts to address potential sources of bias	9
Study size	10	Explain how the study size was arrived at	8-9
Quantitative variables	11	Explain how quantitative variables were handled in the analyses. If applicable, describe which groupings were chosen and why	9
Statistical methods	12	(*a*) Describe all statistical methods, including those used to control for confounding	9 + supplementary text
		(*b*) Describe any methods used to examine subgroups and interactions	9
		(*c*) Explain how missing data were addressed	Supplementary text 1
		(*d*) If applicable, explain how loss to follow-up was addressed	

		(*e*) Describe any sensitivity analyses	
**Results**			
Participants	13*	(a) Report numbers of individuals at each stage of study—eg numbers potentially eligible, examined for eligibility, confirmed eligible, included in the study, completing follow-up, and analysed	11 + supplementary figure 6
		(b) Give reasons for non-participation at each stage	Supplementary figure 6
		(c) Consider use of a flow diagram	Supplementary figure 6
Descriptive data	14*	(a) Give characteristics of study participants (eg demographic, clinical, social) and information on exposures and potential confounders	11-12
		(b) Indicate number of participants with missing data for each variable of interest	Supplementary figures 1-5
		(c) Summarise follow-up time (eg, average and total amount)	11
Outcome data	15*	Report numbers of outcome events or summary measures over time	11
Main results	16	(*a*) Give unadjusted estimates and, if applicable, confounder-adjusted estimates and their precision (eg, 95% confidence interval). Make clear which confounders were adjusted for and why they were included	12-14
		(*b*) Report category boundaries when continuous variables were categorized	
		(*c*) If relevant, consider translating estimates of relative risk into absolute risk for a meaningful time period	
Other analyses	17	Report other analyses done—eg analyses of subgroups and interactions, and sensitivity analyses	
**Discussion**			
Key results	18	Summarise key results with reference to study objectives	15
**Limitations**			
Interpretation	20	Give a cautious overall interpretation of results considering objectives, limitations, multiplicity of analyses, results from similar studies, and other relevant evidence	16-18
Generalisability	21	Discuss the generalisability (external validity) of the study results	18
**Other information**			
Funding	22	Give the source of funding and the role of the funders for the present study and, if applicable, for the original study on which the present article is based	19-20

*Give information separately for cases and controls in case-control studies and, if applicable, for exposed and unexposed groups in cohort and cross-sectional studies.

For peer review only

Note: An Explanation and Elaboration article discusses each checklist item and gives methodological background and published examples of transparent reporting. The STROBE checklist is best used in conjunction with this article (freely available on the Web sites of PLoS Medicine at http://www.plosmedicine.org/, Annals of Internal Medicine at http://www.annals.org/, and Epidemiology at http://www.epidem.com/). Information on the STROBE Initiative is available at www.strobe-statement.org.

For peer review only

BMJ Open

Cardiovascular risk prediction using physical performance measures in COPD: results from a multi-centre observational study.

Journal:	*BMJ Open*
Manuscript ID	bmjopen-2020-038360.R2
Article Type:	Original research
Date Submitted by the Author:	12-Aug-2020
Complete List of Authors:	Fermont, Jilles; University of Cambridge, Department of Medicine<br>Fisk, Marie; University of Cambridge<br>Bolton, Charlotte; University of Nottingham<br>MacNee, William; The University of Edinburgh<br>Cockcroft, John; Columbia University Medical Center<br>Fuld, Jonathan; Cambridge University Hospitals NHS Foundation Trust, Centre for Self Management Support<br>Cheriyan, Joseph; University of Cambridge Medicine, Division of Experimental Medicine & Immunotherapeutics<br>Mohan, Divya; GSK R&D<br>Mäki-Petäjä, Kaisa ; University of Cambridge<br>Al-Hadithi, Ali ; University of Cambridge,<br>Tal-Singer, Ruth; GSK R&D<br>Müllerova, Hana; GSK R&D<br>Polkey, Mike; Royal Brompton Hospital<br>Wood, Angela; University of Cambridge<br>McEniery, Carmel M.; University of Cambridge<br>Wilkinson, Ian; University of Cambridge; Cambridge Clinical Trials Unit,
**Primary Subject Heading**:	Respiratory medicine
Secondary Subject Heading:	Cardiovascular medicine, Epidemiology, Diagnostics, Public health
Keywords:	Chronic airways disease < THORACIC MEDICINE, EPIDEMIOLOGY, RESPIRATORY MEDICINE (see Thoracic Medicine), Cardiac Epidemiology < CARDIOLOGY, PRIMARY CARE

SCHOLARONE™
Manuscripts

I, the Submitting Author has the right to grant and does grant on behalf of all authors of the Work (as defined in the below author licence), an exclusive licence and/or a non-exclusive licence for contributions from authors who are: i) UK Crown employees; ii) where BMJ has agreed a CC-BY licence shall apply, and/or iii) in accordance with the terms applicable for US Federal Government officers or employees acting as part of their official duties; on a worldwide, perpetual, irrevocable, royalty-free basis to BMJ Publishing Group Ltd ("BMJ") its licensees and where the relevant Journal is co-owned by BMJ to the co-owners of the Journal, to publish the Work in this journal and any other BMJ products and to exploit all rights, as set out in our licence.

The Submitting Author accepts and understands that any supply made under these terms is made by BMJ to the Submitting Author unless you are acting as an employee on behalf of your employer or a postgraduate student of an affiliated institution which is paying any applicable article publishing charge ("APC") for Open Access articles. Where the Submitting Author wishes to make the Work available on an Open Access basis (and intends to pay the relevant APC), the terms of reuse of such Open Access shall be governed by a Creative Commons licence – details of these licences and which Creative Commons licence will apply to this Work are set out in our licence referred to above.

Other than as permitted in any relevant BMJ Author's Self Archiving Policies, I confirm this Work has not been accepted for publication elsewhere, is not being considered for publication elsewhere and does not duplicate material already published. I confirm all authors consent to publication of this Work and authorise the granting of this licence.

For peer review only

Cardiovascular risk prediction using physical performance measures in COPD: results from a multi-centre observational study.

Jilles M. Fermont[1,2$], Marie Fisk[1], Charlotte E. Bolton[3], William Macnee[4], John Cockcroft[5], Jonathan Fuld[6], Joseph Cheriyan[1], Divya Mohan[7], Kaisa M. Mäki-Petäjä[1], Ali B.A.K. Al-Hadithi[1], Ruth Tal-Singer[7], Hana Müllerova[8], Michael I. Polkey[9], Angela M. Wood[2,10-13], Carmel McEniery[1], Ian B. Wilkinson[1,14], on behalf of the ERICA consortium.

[1]Division of Experimental Medicine and Immunotherapeutics, Department of Medicine, University of Cambridge, Cambridge, United Kingdom;

[2]British Heart Foundation Cardiovascular Epidemiology Unit, Department of Public Health and Primary Care, University of Cambridge, Cambridge, United Kingdom;

[3]Division of Respiratory Medicine and NIHR Nottingham BRC respiratory theme, University of Nottingham, Nottingham, United Kingdom;

[4]Centre for Inflammation Research, Queen's Medical Research Institute, University of Edinburgh, Edinburgh, Scotland United Kingdom;

[5]Department of Cardiology, Columbia University Medical Centre, New York, United States;

[6]Department of Respiratory Medicine, Cambridge University Hospitals NHS Foundation Trust, Cambridge, United Kingdom;

[7]Medical Innovation, Value Evidence Outcomes, GSK R&D, PA, United States;

[8]GSK R&D, Uxbridge, United Kingdom;

[9]Department of Respiratory Medicine, Royal Brompton Hospital, London, United Kingdom;

[10]British Heart Foundation Centre of Research Excellence, University of Cambridge, Cambridge, United Kingdom;

[11]National Institute for Health Research Blood and Transplant Research Unit in Donor Health and Genomics, University of Cambridge, Cambridge, United Kingdom;

[12]National Institute for Health Research Cambridge Biomedical Research Centre, University of Cambridge and Cambridge University Hospitals, Cambridge, United Kingdom;

[13]Health Data Research UK Cambridge, Wellcome Genome Campus and University of Cambridge, Cambridge, United Kingdom;

[14]Cambridge Clinical Trials Unit, Cambridge University Hospitals NHS Foundation Trust, Addenbrooke's Hospital, Cambridge, United Kingdom.

For peer review only

$Corresponding author: Jilles M. Fermont, Division of Experimental Medicine and Immunotherapeutics, Department of Medicine, University of Cambridge, Cambridge CB1 8RN, United Kingdom. Tel: +44 (0)12 2333 6806. E-mail: jmf88@medschl.cam.ac.uk.

Word count: 3398

Keywords: Chronic Obstructive Pulmonary Disease; COPD; Biomarkers; Prognosis; Cardiovascular Diseases.

For peer review only

ABSTRACT

Objectives Although cardiovascular disease (CVD) is a common co-morbidity associated with chronic obstructive pulmonary disease (COPD), it is unknown how to improve prediction of cardiovascular (CV) risk in COPD individuals. Traditional CV risk scores have been tested in different populations but not uniquely in COPD. The potential of alternative markers to improve CV risk prediction in COPD individuals is unknown. We aimed to determine the predictive value of conventional CVD risk factors in COPD, and to determine if additional markers improve prediction beyond conventional factors.

Design Data from the ERICA cohort, which enrolled 729 GOLD stage II-IV COPD individuals were used. Linked hospital episode statistics and survival data were prospectively collected for a median 4.6 years follow-up.

Setting Five UK centres interested in COPD.

Participants Population-based sample including 714 individuals with spirometry-defined COPD, smoked at least ten pack years and who were clinically stable for >4 weeks.

Interventions Baseline measurements included aortic pulse wave velocity (aPWV), carotid intima media thickness (CIMT), C-reactive protein (CRP), fibrinogen, spirometry and BODE-Index, six-minute walk test (6MWT) and four-metre gait speed (4MGS) test.

Primary and secondary outcome measures New occurrence (first event) of fatal or non-fatal hospitalised CVD, and all-cause and cause-specific mortality.

Results Out of 714 participants, 192 (27%) had CV-hospitalisation and six died due to CVD. The overall CV risk model C-statistic was 0.689 (95% CI 0.688 to 0.691). Aortic pulse wave velocity and CIMT had no association with study outcome; nor improved model prediction. CRP, fibrinogen, GOLD stage, BODE Index, 4MGS and 6MWT were associated with the outcome; independently of conventional risk factors ($p < 0.05$ for all). However, only 6MWT improved model discrimination (C = 0.727 [0.726 to 0.728]).

For peer review only

Conclusions Poor physical performance defined by the 6MWT improves prediction of CV hospitalisation in COPD individuals.

Trial registration Clinical Trials Gateway (ID 11101).

For peer review only

ARTICLE SUMMARY

Strengths and limitations of this study

- This is the first study assessing the utility of conventional cardiovascular (CV) disease risk factors for CV risk prediction and the value of additional markers of risk within a COPD cohort.
- Patient level cohort data were linked to hospital admission data (i.e. hospital episode statistics) obtained from the National Health Services in England, Scotland and Wales, and Office for National Statistics record of mortality with analyses limited to five years of follow-up.
- Hospitalised cardiovascular episodes were coded based on ICD-10 classifications extracted from both primary and secondary positions.
- A multivariable prediction model, with 10-fold cross validation and 200 replications, was used to evaluate a wide range of cardiovascular and physical performance biomarkers.
- Generalisability of our results is limited to those with moderate COPD in the UK with NHS hospitalisations.

For peer review only

INTRODUCTION

Chronic obstructive pulmonary disease (COPD) is associated with a two to three-fold increased risk of coronary artery disease and other cardiovascular (CV) comorbidities.[1,2] COPD and cardiovascular disease (CVD) are common conditions that share important common risk factors, such as smoking and physical inactivity, and both tend to affect older people. In addition, both conditions are often associated with raised systemic inflammatory markers and increased arterial stiffness.[3,4]

Given the global healthcare burden associated with CVD,[5] there is incentive to improve the accuracy of CV risk prediction in different populations. Individuals with COPD may be considered constitutively at high CV risk given their age and smoking history. However, it is unknown whether classic CV risk prediction models such as the Framingham general CV risk score, which predicts an individual's ten year risk of developing CVD based on an algorithm of weighted risk factors and has been tested in a number of different populations,[6–9] perform well in individuals with COPD.[10,11]

Conceptually, the performance of the Framingham model might be improved by additional measures. Candidate measurements include surrogate CV risk markers that impart mechanistic information (i.e. aortic pulse wave velocity (aPWV) or carotid intima-media thickness (CIMT)), inflammatory markers (i.e. C-reactive protein (CRP)) or measures of physical performance that enhance CV risk prediction in COPD individuals. In fact, the value of these measures in CV risk prediction have been explored in different population groups. For example, the six-minute walk test (6MWT) significantly improved risk prediction in patients with stable coronary disease.[12] In individuals with intermediate CV risk but without CVD, adding CRP or fibrinogen to conventional risk factors modestly improved CV event prediction.[13] A meta-analysis of aPWV studied in different disease cohorts showed it improved CV event prediction independently of conventional risk factors.[14] In contrast, CIMT measurement, although associated with CV risk factors, did not significantly improve risk prediction beyond traditional factors in individuals with hypertension.[15] Given that individuals with COPD, in addition to having high CV risk based on conventional factors, also have increased aPWV, CIMT[4], and increased inflammatory markers,[16] as well as reduced lung function which is associated with increased CV risk in the general population[17] and reduced physical performance,[18,19] the clinical significance of

For peer review only

these findings in relation to CV risk prediction in individuals with COPD is an important question to address.

The aims of our study were to firstly determine the predictive value of conventional CVD risk factors for CV risk, defined by new occurrence (first event since study enrolment) of fatal or non-fatal hospitalised CVD in individuals with stable global initiative for chronic obstructive lung disease (GOLD) stage II-IV[20] COPD. A secondary aim was to determine whether addition of alternative CV measures (i.e. aPWV and CIMT), inflammatory markers (i.e. CRP and fibrinogen), COPD severity (i.e. GOLD stage and BODE Index), as well as physical performance tests commonly used in COPD research (i.e. 6MWT and four-metre gait speed (4MGS)) improved the predictive value of a CV risk model based on conventional CVD risk factors for CV risk prediction. We addressed these questions in the Evaluation of the Role of Inflammation in Chronic Airways disease (ERICA) COPD cohort using study data and linked UK electronic health records.

For peer review only

METHODS

Study design and participants

The ERICA study is a multi-centre observational, cohort study with 729 individuals with stable GOLD stage II-IV[20] COPD, established to identify important CV and physical performance biomarkers that could be targeted to improve the outcomes of COPD individuals. Participants had a clinical diagnosis of COPD, smoking history of at least ten pack years, post-bronchodilator forced expiratory lung volume in 1 second ($FEV_{1)}$ / forced vital capacity (FVC) ratio <0.7 and FEV_1 ≤80% of predicted normal lung function, and were aged >40 years old and clinically stable for >4 weeks. Full details of the protocol have been provided elsewhere.[21] Baseline data captured included demographics, spirometry, blood circulating biochemical markers, measures of arterial stiffness (i.e. aPWV and augmentation index (AIx)), CIMT and physical performance (i.e. 4MGS and 6MWT). Individuals in the ERICA study were linked with UK National Health Services (NHS) electronic healthcare records (i.e. hospital episode statistics (HES[1]))[22] and Office for National Statistics death data through anonymised IDs provided by the NHS.

Clinical measures

After four hours of fasting, with no bronchodilators for six hours, and ten minutes of supine rest, carotid-femoral aPWV and AIx measurements were taken using a SphygmoCor system as previously described.[23] Carotid intima-media thickness of the common carotid arteries was measured using B-mode ultrasound at a distance of one cm from the carotid bulb with a 7–12 MHz linear probe.[24] The thickest artery of the two was included in the analysis. Fasting blood samples were taken for biochemical analysis including plasma fibrinogen, serum CRP and glucose. Physical performance measures 6MWT[25] and 4MGS[26] were assessed according to guidelines. Diabetes status and anti-hypertensive treatment were self-reported at baseline study visit. Disease severity was defined according to GOLD classification.[20] Points for the BODE (Body mass index (BMI), airflow Obstruction, Dyspnoea, and Exercise capacity) Index were assigned as described by Celli *et al.*[27]

Cardiovascular hospitalisation and mortality

[1] Hospital episode statistics (HES) data is a database that includes details of all hospital admissions, accident and emergency department visits and outpatient appointments at an individual patient level.

For peer review only - http://bmjopen.bmj.com/site/about/guidelines.xhtml

Cardiovascular hospitalisation and mortality data were extracted from the linked hospital admission data and death certificates. Non-fatal CV episodes were extracted from both primary and secondary international statistical classification of diseases and related health problems 10th revision coding (ICD-10) positions. Causes of death were adjudicated by CV and pulmonary physicians. We defined the primary outcome as first reported occurrence (since study enrolment) of fatal or non-fatal hospitalised CVD, where CVD was defined as diseases of the arteries, stroke or heart failure (see supplementary table 1) based on classifications used by the Emerging Risk Factors Collaboration.[28] Time to primary outcome was calculated from the difference between the baseline visit date (starting December 2011) and either the date of death or first hospitalised CV attendance up to November 2017, when follow-up discontinued. Secondary outcomes of interest were all-cause and cause-specific mortality (defined as CV, pulmonary, cancer, or other).

Risk factors of interest

Conventional CVD risk factors included age, sex, self-reported smoking status [current/ex-smoker], high-density lipoprotein (HDL) cholesterol, total cholesterol, systolic blood pressure (SBP), diabetes [yes/no] and treatment for high blood pressure [yes/no]. We also assessed the addition of the following risk factors: BMI, aPWV and CIMT, fasting glucose, CRP and fibrinogen, COPD severity (i.e. GOLD stage and BODE Index), and measures of physical performance (i.e. 6MWT and 4MGS).

Statistical analysis

Hazard ratios with 95% confidence intervals (CI) were estimated using Cox regression models stratified by study centre. Age and sex were added to all models. To quantify the independent association of CIMT we further included systolic blood pressure. For aPWV we also included mean arterial pressure and resting heart rate. For AIx we additionally included resting heart rate and height. Proportional hazards were assessed by Schoenfeld's global tests. We assessed the relationships of the new markers and outcomes and consequently log-transformed the following risk factors: CRP, fibrinogen and glucose. Hazard ratios for log-transformed risk factors represent a twofold increase in the risk factor, whereas others are presented as a change in unit. We evaluated the predictive value of new markers added to the conventional CVD risk factors using measures of discrimination (i.e. Harrell's C-statistic)[29,30] and calibration (i.e. Gronnesby and Borgan test and Brier score). Brier

For peer review only - http://bmjopen.bmj.com/site/about/guidelines.xhtml

scores range from 0-1 (0 is perfect accuracy and 1 perfect inaccuracy), and allows to compare performance of a model with a reference model. The C-statistic is a measure for validating the discriminative ability of a model. Value ranges from 0.5-1.0 (1.0 is perfect prediction and 0.5 a random guess). A higher score indicates better discriminative ability of the model. To optimise efficiency and avoid optimism from internal validation in small samples, we used 10-fold cross validation with 200 replications.[31] See online supplementary text 1 and supplementary figures 1-5 for further statistical analyses details.

Observational data is reported according to the STROBE statement.[32] All tests were two-sided and statistical significance were defined by 95% CI for hazard ratios not traversing 1 or p<0.05. Our analyses were performed using Stata version 13 (College Station. Texas) and R (R Foundation).

For peer review only

RESULTS

Of the 729 individuals included in the study, 714 (98%) could be linked with hospital admission and survival records, and were included in the analysis (online supplementary figure 6). The median age was 67 (interquartile range 62-73) years and 434 (61%) individuals were male (table 1). A third (n = 218) of the cohort smoked, 12% (n = 82) had self-reported diabetes, 34% (n = 245) were taking anti-hypertensive medications, and 31% (n = 224) were taking cholesterol-lowering medications at baseline. The median FEV_1 was 1.3 (0.9-1.7) litres (mean ± SD = 1.34 ± 0.53). In total, 192 individuals (27%) had a first event of CV hospitalisation[2] (n = 116 (60%) were in ICD-10 secondary coding position) during median follow-up for 4.6 years and six individuals had CV death without any preceding CV episode. Cardiovascular hospitalisation accounted for the majority (97%) of events analysed. The CV incidence rate was 6.7 (95% CI 5.8 to 7.7) per 100 person-years. See online supplementary tables 1-2 for categorisation of different admission codes for CV hospitalisation.

Table 1 Baseline characteristics (No. = 714).

Characteristic	Summary measures
**Conventional CVD risk factors**	
Age (yrs.)	67 (62 to 73)
Male	434 (61)
Current smoker	218 (31)
HDL (mmol/L)	1.4 (1.2 to 1.7)
Cholesterol (mmol/L)	5.0 (4.3 to 5.8)
SBP (mmHg)	142 (131 to 154)
Diabetes mellitus	82 (12)
Drugs to treat hypertension	245 (34)
**CV measures**	
aPWV (m/sec)	9.8 (8.4 to 11.8)
CIMT (mm)	0.81 (0.71 to 0.96)

[2] Peripheral arterial disease (n = 9), diseases of arteries, arterioles and capillaries (n = 7), angina (n = 21), unstable angina (n = 3), coronary heart disease not otherwise specified (n = 63), acute myocardial infarction (MI), and certain current complications following acute MI (n = 11), cerebral infarction (n = 11), stroke, not specified as haemorrhage or infarction (n = 3), other stroke (n = 18), heart failure (n = 32), and abdominal aortic aneurysm (n = 8).

For peer review only - http://bmjopen.bmj.com/site/about/guidelines.xhtml

For peer review only

Alternative measures	
CRP (mg/L)	1.21 (0.47 to 2.01)
Fibrinogen (g/dL)	1.22 (1.06 to 1.36)
Glucose (mmol/L)	1.59 (1.46 to 4.59)
BMI (kg/m^2)	27 (23 to 31)
GOLD (stage)	2 (2 to 3)
4MGS (m/s)	0.95 (0.77 to 1.14)
6MWT distance (metre)	366 (255 to 440)
BODE (point)	3 (1−5)

Values are given as the median and interquartile range (IQR), or No. of cases (%). CVD = cardiovascular disease. HDL = high-density lipoprotein. SBP = systolic blood pressure. CV = cardiovascular. aPWV = carotid-femoral aortic pulse wave velocity. CIMT = carotid intima-media thickness. CRP = C-reactive protein. BMI = body mass index. GOLD = global initiative for obstructive lung disease. 4MGS = four-metre gait speed. 6MWT = six-minute walk test. BODE = BMI, obstruction (FEV_1), dyspnoea (Medical Research Council), exercise (6MWT).

Conventional cardiovascular disease risk factors

Of the conventional CVD risk factors, age and treatment for high blood pressure had significant positive associations with the study's outcome whereas SBP had a significant negative association and other risk factors (i.e. sex, smoking status, cholesterol, HDL cholesterol and diabetes) were not significantly associated with CV hospitalisation. Use of hypertension drug treatment followed by age and total cholesterol contributed most to the discriminative ability of the model. The overall discriminative ability of the CV risk model had a C-statistic of 0.689, 95% CI 0.688 to 0.691; figure 1 and online supplementary table 2).

PLACE FIGURE 1 HERE

Surrogate cardiovascular risk markers

For peer review only - http://bmjopen.bmj.com/site/about/guidelines.xhtml

Except for AIx, neither aPWV nor CIMT were significantly associated with CV hospitalisation, after including conventional CVD risk factors (figure 2 and online supplementary table 3). Moreover, none of the CV risk markers significantly changed the discriminative ability of the CV risk model.

PLACE FIGURE 2 HERE

Physical performance measures, COPD severity and inflammatory markers

Multivariable analysis identified that poor physical performance (i.e. reduced 6MWT distance and slower 4MGS) were significantly associated with increased CV hospitalisation, independently of conventional CVD risk factors. With the exception of glucose and BMI, severity of COPD defined by higher BODE Index and GOLD stage, as well as inflammatory markers (i.e. CRP and fibrinogen) all had significant positive associations with CV hospitalisations independently of conventional CVD risk factors.

Predictive modelling indicated significant improvement in risk discrimination when adding BMI (C = 0.698, 95% CI 0.696 to 0.699), BODE (C = 0.705, 95% CI 0.704 to 0.707), 4MGS (C = 0.712, 95% CI 0.710 to 0.713) or 6MWT (C = 0.727, 95% CI 0.726 to 0.728) to the CV risk model, but not GOLD stage, inflammatory markers or glucose (figure 3 and online supplementary table 4). Calibration tests indicate good model fit (figure 3). Adding BMI, 4MGS, 6MWT and BODE collectively to the CV risk model resulted in a C-statistic of 0.731 (95% CI 0.729 to 0.732), indicating 6MWT primarily accounted for improved discriminative ability of the model. The model including 6MW had a better Brier score relative to the CV risk model (0.123 vs. 0.129, respectively).

PLACE FIGURE 3 HERE

All-cause and cause-specific mortality

For peer review only

There were 144 deaths in total (nearly 20% of the cohort), over a median follow-up period of 4.6 years. The majority of deaths were in men (n = 96, 67%) and pulmonary disease was the leading cause in both sexes (table 2). Pulmonary disease accounted for 65% of deaths in women and 50% in men, followed by cancer (19% in women, 27% in men), CV (6% and 16%), and other (10% and 7% in women and men respectively).

Table 2 Mortality in the ERICA cohort.

**Cause-specific mortality**	Female No. (%)	Male No. (%)	Total No. (%)
Pulmonary	31 (65)	48 (50)	79 (55)
Cardiovascular	3 (6)	15 (16)	18 (13)
Cancer	9 (19)	26 (27)	35 (24)
Other	5 (10)	7 (7)	12 (8)
**All-cause**	48 (100)	96 (100)	144 (100)

Deaths recorded over median follow up of 4.6 years.

For peer review only

DISCUSSION

This is the first study assessing the utility of conventional CVD risk factors for CV risk prediction and the value of additional markers of risk within a COPD cohort. Novel findings include (i) poor physical performance improved the discriminative power when added to the CV risk model; (ii) BODE Index, GOLD stage, 4MGS and systemic inflammatory markers were positively associated with CV hospitalisations independently of conventional CVD risk factors, although they collectively improved the model's discriminative ability marginally; (iii) of the conventional CVD risk factors; age, SBP and use of anti-hypertensives were positively associated with study outcome. However, age, cholesterol and use of anti-hypertensives contributed most to the model's prognostic power. Systolic blood pressure had a significant negative association with CV hospitalisations but did not add to the model's predictive ability. Furthermore, aPWV and CIMT, despite providing *in-vivo* mechanistic information about the arterial system, had no significant association with CV hospitalisations, whereas AIx did. Therefore, these data suggest that in COPD, physical performance (assessed by 6MWT) contributes to CV risk estimation defined predominantly by non-fatal CV hospitalisations. Finally, CVD contributed to only a small number of deaths at a median follow-up of 4.6 years. Pulmonary disease, followed by cancer were the major causes of death in both men and women with COPD.

We observed a C-statistic of 0.689 based on the conventional CV risk model, which increased to a maximum of 0.731 when adding additional markers; this increment was primarily due to 6MWT. These C-statistic values indicate a moderate predictive ability of the models for the study's outcome. For perspective, a C-statistic of 0.53 was observed in a Framingham model in the very elderly (aged 85-plus; the median age of our cohort was 67 years) without prior CVD, indicating it does not predict CV mortality in this group.[6] However, in primary care individuals without CVD in the Framingham study, a C-statistic of 0.76 for an outcome of general CVD (i.e. coronary heart disease, stroke, peripheral artery disease, or heart failure), demonstrated good discrimination.[10] Since our cohort included individuals with CVD at baseline, and different CVD risk factors and CV outcomes are evaluated in various studies, no direct comparison of study results can be made. That aPWV was not predictive in our cohort contrasts with findings reported by Ben-Shlomo *et al.* However, from the 17 included cohorts in their study, none were COPD cohorts, the mean age of the cohorts was lower, and the proportion of individuals taking drugs to treat hypertension was higher.[14]

Overall, our study emphasises the importance of physical function as a predictor of CV risk. Tests such as 6MWT or 4MGS are proxy measures of overall mobility and physical functioning.[33] Exercise capacity and CV fitness are known to be associated with fatal- and non-fatal CVD,[34] whilst exercise-based cardiac rehabilitation reduces risk of CV events.[35] The 6MWT distance is prognostic in patients with stable coronary heart disease,[12] and those with moderate-to-severe heart failure.[36] In our cohort poor physical function improved discriminative ability of the CV risk model beyond conventional CVD factors. This has implications for clinical practice in CV risk assessment for COPD individuals, suggesting it may be helpful to incorporate physical performance into clinical assessment. Especially those aged under 65 years may benefit most from active CVD assessment, according to Morgan *et al*.[37] However, given that 6MWT can be logistically challenging to set up and time-consuming,[38] the faster and simpler 4MGS that also significantly improved the C-statistic could be used as a simpler alternative. In elderly with CVD, 4MGS is comparable to 6MWT in predicting all-cause mortality.[39]

The BODE Index also significantly improved the C-statistic of the CV risk model. However, this was primarily due to the 6MWT component. In fact, although GOLD stage had a significant positive association with the study outcome, this did not improve prognostic power of the model. Therefore, despite association between airflow limitation and CV risk in general population studies,[17,40] in patients with COPD, physical performance assessment rather than another component of BODE (i.e. spirometry) adds value to CV risk prediction.

Although we did not find an association between inflammatory markers and surrogate markers of CV risk in the baseline cross-sectional component of the ERICA study,[41] both CRP and fibrinogen were associated with CV hospitalisations captured over nearly five years follow-up. That the associations remained significant after including conventional CVD risk factors indicate potential value for identifying high-risk individuals within a COPD population. The inverse relationship of SBP was an unexpected finding. One hypothesis is that SBP has a J-shaped curve for CV events and mortality.[42] Therefore, in this cohort, lower SBP might be a marker of sickness and frailty, hence its association with CV hospitalisations.

For peer review only

A third of the cohort had a CV related hospitalisation during follow-up and the majority had pre-existing CV comorbidity at baseline. The high numbers of CV hospitalisations, gives perspective of what this comorbidity incurs for patients and the huge healthcare costs involved. In contrast to the sizeable number of CV hospitalisations, was the small number of CV deaths. Gayle *et al.*, previously reported that CV-related mortality in chronic lung patients had already started to decline in England.[43] This may reflect better CV risk management reducing CV mortality. Importantly though, such risk management does not seem to impact CV morbidity, and is an area that requires future research to determine the optimum approach to impact CV morbidity in COPD individuals.

Nearly 20% of the cohort died during follow-up. This is comparable to the TORCH study where approximately 15% of the cohort died over 3 years of follow-up.[2] Pulmonary disease, followed by cancer accounted for proportionally more deaths in our cohort compared with TORCH, whereas CV-specific mortality was less (10-15% vs 27% in TORCH). Reasons for these findings are not entirely understood but the sizeable proportion of our cohort already on medicines for dyslipidaemia and blood pressure control may be an important factor.

Our study has limitations. The conventional CVD risk factors are usually used to predict CV risk defined by development of CVD. However, the majority of our participants already had CVD and were on CV medications, which may be a confounding factor impacting the discriminative ability of CVD risk factors. Moreover, in our study, CV risk was defined differently (as CV hospitalisation with CV mortality). Hospitalised CV episodes were coded based on ICD-10 classifications[28] extracted from both primary and secondary positions. Notably, most CV hospitalisations were recorded in secondary positions, indicating that the primary admission might be related to something else. Due to the few CV events recorded in the primary position, we were unable to conduct a sensitivity analysis including CV events in the primary position only. The study period covered the time from study enrolment until the end of study, or death. Some individuals, however, may have been admitted to hospital for CV events before study enrolment. We were unable to obtain HES data outside this period. Competing risks may have occurred, for example, individuals who died of other causes may not have experienced a CV event during the study period for this reason. However, our study did not have sufficient statistical

For peer review only - http://bmjopen.bmj.com/site/about/guidelines.xhtml

power to assess this. It is possible our models predict non-CV hospitalisation due to, for example, potential cross-contamination of COPD and CV hospitalisation as a result of misclassification of morbidity and mortality that often occurs when data are obtained from routine sources.[44] Deprivation scores may be another important determinant of CV risk in COPD individuals that is worth evaluation in future research,[45] although the relatively small cohort size of our study meant we did not include this in the analysis.

Furthermore, follow-up time for the study was a maximum of five years, whereas risk scores such as Framingham Risk Score and QRISK calculate a 10-year risk. Hence, the prognostic value of variables may alter with a different time horizon and depends on the extent of time trends in the new biomarkers. For example, a too short time period may result in an insufficient number of events, whilst over a longer time period, e.g. 20 years, the predictive ability would diminish because ageing is a strong predictor. We did not have access to an independent validation cohort but used cross-validation techniques instead. Generalisability of our results is limited to those with moderate COPD in the UK with NHS hospitalisations.

CONCLUSION

In a UK COPD cohort, poor physical performance assessed by 6MWT or 4MGS and inflammatory biomarkers are associated with subsequent CV-related hospitalisations, independently of conventional CVD risk factors. Importantly, poor physical performance (defined primarily by 6MWT), also significantly improved the predictive discrimination of the CV risk model. These data suggest an assessment of physical performance may enhance CV risk evaluation in individuals with COPD.

ACKNOWLEDGMENTS

The views and opinions expressed are those of the authors and do not necessarily reflect those of the University of Cambridge, the NHS, the NIHR, the Department of Health and Social Care, or other parent institutions of the authors. The corresponding author and IBW had full access to all the data in the study and takes responsibility for the integrity of the data and the accuracy of the data analysis.

For peer review only

Contributors: The following authors made substantial contributions to the conceptualisation or design: JMF, RTS, HM, MIP, AMW and IBW; data curation: JMF, IBW, KMP and AAH; formal analysis: JMF; interpretation: JMF, MF and IBW; funding acquisition: RTS, MIP and IBW; investigation: JMF, MF, CEB, WM, JRC, JF, JC, DM, KMP, AAH, MIP, CMM and IBW; methodology: JMF, AMW, IBW; project administration: JMF, RTS, MIP and IBW; resources: JMF, MF, CEB, WM, JRC, DM, MIP, CMM and IBW; software: JMF; supervision: DM, RTS, HM, MIP, AMW and IBW; validation: JMF; visualisation: JMF; and writing – original draft preparation: JMF. All authors contributed to the reviewing and editing of the manuscript.

Competing interests: All authors have completed the ICMJE uniform disclosure form at www.icmje.org/coi_disclosure.pdf and declare: GSK, a consortium partner, funded JMFs PhD. HM, RTS and DM were employees of GSK at the time this work was completed and own GSK shares and stock options. JC is employed by Cambridge University Hospitals NHS Foundation Trust and is obligated to spend 50% of his time on GSK clinical trial activity, representing a significant relationship; however, he receives no other benefits or compensation from GSK. MIP received grants from GSK outside the submitted work. IBW received grants from GSK during the conduct of the study and outside the submitted work.

Funding: This work was supported by a grant (9157-61188) from Innovate UK (formerly known as Technology Strategy Board) with contributory funding in kind (e.g. scientific expertise and meeting rooms) from GSK, a consortium partner, who also funded the corresponding author's PhD. As a consortium partner, GSK was involved in the study design, data analysis, decision to publish, and preparation of the manuscript. The specific roles of all authors are articulated in the 'author contributions' section. RTS was a co-investigator on the grant and as a consortium member was involved in the decision to publish, and preparation of the manuscript. IBW, JC and CMM acknowledge funding support from the NIHR Cambridge Comprehensive Biomedical Research Centre. CEB is supported by the NIHR Nottingham BRC respiratory theme. This work was supported by core funding from: the UK Medical Research Council (MR/L003120/1), the British Heart Foundation (RG/13/13/30194; RG/18/13/33946) and the National Institute for Health Research [Cambridge Biomedical Research Centre at the Cambridge University Hospitals NHS Foundation Trust] [*]. This

For peer review only

work was also supported by Health Data Research UK, which is funded by the UK Medical Research Council, Engineering and Physical Sciences Research Council, Economic and Social Research Council, Department of Health and Social Care (England), Chief Scientist Office of the Scottish Government Health and Social Care Directorates, Health and Social Care Research and Development Division (Welsh Government), Public Health Agency (Northern Ireland), British Heart Foundation and Wellcome. *The views expressed are those of the authors and not necessarily those of the NHS, the NIHR or the Department of Health and Social Care.

Data availability: The dataset underlying these findings, with de-identified participant data (including the data dictionary), are available to interested and qualified researchers upon request and can be obtained from the Cambridge Clinical Trials Unit. Access to hospital episode statistics requires a data sharing agreement with the National Health Services. For data access, please contact cctu@addenbrookes.nhs.uk.

Patient consent for publication: Informed patient consent was obtained in writing from all study participants and permits the publishing of all data included in this article. The investigation conforms with the principles outlined in the Declaration of Helsinki. Ethical approval was granted by the National Research Ethics Service Committee East of England - Cambridge South (11/EE/0357).

Patient and public involvement: Patients and public were not involved in the design, conduct or reporting of this study.

For peer review only

REFERENCES

1 Chen W, Thomas J, Sadatsafavi M, *et al.* Risk of cardiovascular comorbidity in patients with chronic obstructive pulmonary disease: a systematic review and meta-analysis. *Lancet Respir Med* 2015;**3**:631–9. doi:10.1016/S2213-2600(15)00241-6

2 McGarvey LP, John M, Anderson JA, *et al.* Ascertainment of cause-specific mortality in COPD: operations of the TORCH Clinical Endpoint Committee. *Thorax* 2007;**62**:411–5.

3 Garcia-Rio F, Miravitlles M, Soriano JB, *et al.* Systemic inflammation in chronic obstructive pulmonary disease: a population-based study. *Respir Res* 2010;**11**:63. doi:10.1186/1465-9921-11-63

4 Fisk M, McEniery CM, Gale N, *et al.* Surrogate Markers of Cardiovascular Risk and Chronic Obstructive Pulmonary Disease: A Large Case-Controlled Study. *Hypertension* 2018;**71**:499–506. doi:10.1161/HYPERTENSIONAHA.117.10151

5 Lozano R, Naghavi M, Foreman K, *et al.* Global and regional mortality from 235 causes of death for 20 age groups in 1990 and 2010: a systematic analysis for the Global Burden of Disease Study 2010. *Lancet* 2012;**380**:2095–128. doi:10.1016/S0140-6736(12)61728-0

6 de Ruijter W, Westendorp RG, Assendelft WJ, *et al.* Use of Framingham risk score and new biomarkers to predict cardiovascular mortality in older people: population based observational cohort study. *BMJ* 2009;**338**:a3083. doi:10.1136/bmj.a3083

7 Wannamethee SG, Shaper AG, Lennon L, *et al.* Metabolic syndrome vs Framingham Risk Score for prediction of coronary heart disease, stroke, and type 2 diabetes mellitus. *Arch Intern Med* 2005;**165**:2644–50. doi:10.1001/archinte.165.22.2644

8 Weiner DE, Tighiouart H, Elsayed EF, *et al.* The framingham predictive instrument in chronic kidney disease. *J Am Coll Cardiol* 2007;**50**:217–24. doi:10.1016/j.jacc.2007.03.037

9 Samia M, F. RR, Richey SA, *et al.* Enhanced Risk Assessment in Asymptomatic Individuals With Exercise Testing and Framingham Risk Scores. *Circulation* 2005;**112**:1566–72. doi:10.1161/CIRCULATIONAHA.105.542993

10 D'Agostino RB, Vasan RS, Pencina MJ, *et al.* General cardiovascular risk profile for use in primary care. *Circulation* 2008;**117**:743–53.

11 Lee HM, Lee J, Lee K, *et al.* Relation Between COPD Severity and Global Cardiovascular Risk in US Adults. *Chest* 2012;**142**:1118–25. doi:10.1378/chest.11-2421

For peer review only - http://bmjopen.bmj.com/site/about/guidelines.xhtml

12 Beatty AL, Schiller NB, Whooley MA. Six-minute walk test as a prognostic tool in stable coronary heart disease: data from the heart and soul study. *Arch Intern Med* 2012;**172**:1096–102. doi:10.1001/archinternmed.2012.2198

13 Emerging Risk Factors Collaboration, Kaptoge S, Di Angelantonio E, *et al.* C-reactive protein, fibrinogen, and cardiovascular disease prediction. *N Engl J Med* 2012;**367**:1310–20. doi:10.1056/NEJMoa1107477

14 Ben-Shlomo Y, Spears M, Boustred C, *et al.* Aortic pulse wave velocity improves cardiovascular event prediction: an individual participant meta-analysis of prospective observational data from 17,635 subjects. *J Am Coll Cardiol* 2014;**63**:636–46. doi:10.1016/j.jacc.2013.09.063

15 Bots ML, Groenewegen KA, Anderson TJ, *et al.* Common carotid intima-media thickness measurements do not improve cardiovascular risk prediction in individuals with elevated blood pressure: the USE-IMT collaboration. *Hypertens (Dallas, Tex 1979)* 2014;**63**:1173–81. doi:10.1161/HYPERTENSIONAHA.113.02683

16 Gan WQ, Man SFP, Senthilselvan A, *et al.* Association between chronic obstructive pulmonary disease and systemic inflammation: a systematic review and a meta-analysis. *Thorax* 2004;**59**:574–80. doi:10.1136/thx.2003.019588

17 Hole DJ, Watt GC, Davey-Smith G, *et al.* Impaired lung function and mortality risk in men and women: findings from the Renfrew and Paisley prospective population study. *BMJ* 1996;**313**:711–6. doi:10.1136/bmj.313.7059.711

18 Sciurba F, Criner GJ, Lee SM, *et al.* Six-Minute Walk Distance in Chronic Obstructive Pulmonary Disease. *Am J Respir Crit Care Med* 2003;**167**:1522–7. doi:10.1164/rccm.200203-166OC

19 Camarri B, Eastwood PR, Cecins NM, *et al.* Six minute walk distance in healthy subjects aged 55–75 years. *Respir Med* 2006;**100**:658–65. doi:10.1016/j.rmed.2005.08.003

20 Global Initiative for Chronic Obstructive Lung Disease (GOLD). Global Strategy for the Diagnosis, Management and Prevention of COPD. 2016. http://goldcopd.org/global-strategy-diagnosis-management-prevention-copd-2016/

21 Mohan D, Gale NS, McEniery CM, *et al.* Evaluating the role of inflammation in chronic airways disease: the ERICA study. *COPD* 2014;**11**:552–9. doi:10.3109/15412555.2014.898031

For peer review only

22 Herbert A, Wijlaars L, Zylbersztejn A, *et al.* Data Resource Profile: Hospital Episode Statistics Admitted Patient Care (HES APC). *Int J Epidemiol* 2017;**46**:1093-1093i. doi:10.1093/ije/dyx015

23 Wilkinson IB, Fuchs SA, Jansen IM, *et al.* Reproducibility of pulse wave velocity and augmentation index measured by pulse wave analysis. *J Hypertens* 1998;**16**:2079–84.https://www.ncbi.nlm.nih.gov/pubmed/9886900

24 Csanyi A, Egervari A. Simple clinical method of average intima-media thickness measurement in the common carotid artery. *Vasa* 1996;**25**:242–8.http://www.ncbi.nlm.nih.gov/pubmed/8795305

25 Laboratories ATSC on PS for CPF. ATS statement: guidelines for the six-minute walk test. *Am J Respir Crit Care Med* 2002;**166**:111–7. doi:10.1164/ajrccm.166.1.at1102

26 Guralnik JM, Simonsick EM, Ferrucci L, *et al.* A short physical performance battery assessing lower extremity function: association with self-reported disability and prediction of mortality and nursing home admission. *J Gerontol* 1994;**49**:M85-94.http://www.ncbi.nlm.nih.gov/pubmed/8126356

27 Celli BR, Cote CG, Marin JM, *et al.* The body-mass index, airflow obstruction, dyspnea, and exercise capacity index in chronic obstructive pulmonary disease. *N Engl J Med* 2004;**350**:1005–12. doi:10.1056/NEJMoa021322

28 Emerging Risk Factors Collaboration. The Emerging Risk Factors Collaboration: analysis of individual data on lipid, inflammatory and other markers in over 1.1 million participants in 104 prospective studies of cardiovascular diseases. *Eur J Epidemiol* 2007;**22**:839–69. doi:10.1007/s10654-007-9165-7

29 Altman DG, Vergouwe Y, Royston P, *et al.* Prognosis and prognostic research: validating a prognostic model. *BMJ* 2009;**338**:b605.

30 Newson RB. Comparing the predictive powers of survival models using Harrell's C or Somers' D. *Stata J* 2010;**10**:339–58.http://www.stata-journal.com/article.html?article=st0198

31 Smith GC, Seaman SR, Wood AM, *et al.* Correcting for optimistic prediction in small data sets. *Am J Epidemiol* 2014;**180**:318–24. doi:10.1093/aje/kwu140

32 von Elm E, Altman DG, Egger M, *et al.* The Strengthening the Reporting of Observational Studies in Epidemiology (STROBE) statement: guidelines for reporting observational studies. *J Clin Epidemiol* 2008;**61**:344–9. doi:10.1016/j.jclinepi.2007.11.008

For peer review only

33 Lord SR, Menz HB. Physiologic, psychologic, health predictors of 6-minute walk performance in older people. *Arch Phys Med Rehabil* 2002;**83**:907–11. doi:10.1053/apmr.2002.33227

34 Newman AB, Simonsick EM, Naydeck BL, *et al.* Association of long-distance corridor walk performance with mortality, cardiovascular disease, mobility limitation, and disability. *Jama-Journal Am Med Assoc* 2006;**295**:2018–26. doi:DOI 10.1001/jama.295.17.2018

35 Anderson L, Oldridge N, Thompson DR, *et al.* Exercise-Based Cardiac Rehabilitation for Coronary Heart Disease Cochrane Systematic Review and Meta-Analysis. *J Am Coll Cardiol* 2016;**67**:1–12. doi:10.1016/j.jacc.2015.10.044

36 Opasich C. Six-minute walking performance in patients with moderate-to-severe heart failure; is it a useful indicator in clinical practice? *Eur Heart J* 2001;**22**:488–96. doi:10.1053/euhj.2000.2310

37 Morgan AD, Zakeri R, Quint JK. Defining the relationship between COPD and CVD: what are the implications for clinical practice? *Ther Adv Respir Dis* 2018;**12**:1753465817750524. doi:10.1177/1753465817750524

38 Holland AE, Spruit MA, Troosters T, *et al.* An official European Respiratory Society/American Thoracic Society technical standard: field walking tests in chronic respiratory disease. *Eur Respir J* 2014;**44**:1428–46. doi:10.1183/09031936.00150314

39 Kamiya K, Hamazaki N, Matsue Y, *et al.* Gait speed has comparable prognostic capability to six-minute walk distance in older patients with cardiovascular disease. *Eur J Prev Cardiol* 2017;**25**:212–9. doi:10.1177/2047487317735715

40 D. SD, Paul MSF. Why Are Patients With Chronic Obstructive Pulmonary Disease at Increased Risk of Cardiovascular Diseases? *Circulation* 2003;**107**:1514–9. doi:10.1161/01.CIR.0000056767.69054.B3

41 Duvoix A, Dickens J, Haq I, *et al.* Blood fibrinogen as a biomarker of chronic obstructive pulmonary disease. *Thorax* 2013;**68**:670–6. doi:10.1136/thoraxjnl-2012-201871

42 SUMMIT Investigators, Brook RD, Byrd JB, *et al.* Blood pressure, heart rate, and mortality in chronic obstructive pulmonary disease: the SUMMIT trial. *Eur Heart J* 2018;**39**:3128–34. doi:10.1093/eurheartj/ehy451

43 Gayle A V, Axson EL, Bloom CI, *et al.* Changing causes of death for patients with chronic respiratory disease in England, 2005-2015. *Thorax* 2019;**74**:483 LP – 491.

For peer review only

doi:10.1136/thoraxjnl-2018-212514

44 Macnee W, Maclay J, McAllister D. Cardiovascular injury and repair in chronic obstructive pulmonary disease. *Proc Am Thorac Soc* 2008;**5**:824–33. doi:10.1513/pats.200807-071TH

45 Pujades-Rodriguez M, Timmis A, Stogiannis D, *et al.* Socioeconomic deprivation and the incidence of 12 cardiovascular diseases in 1.9 million women and men: implications for risk prediction and prevention. *PLoS One* 2014;**9**:e104671–e104671. doi:10.1371/journal.pone.0104671

For peer review only

Figure legends

Figure 1. Conventional cardiovascular disease risk factors at baseline, their hazard ratios and discriminative ability for fatal or non-fatal hospitalised cardiovascular disease.

Values are given as the median and interquartile range (IQR), or No. of cases (%). Baseline data of 714 patients are included. All models are stratified by recruitment site.

[a]Model includes age and sex.

[b]Model includes conventional cardiovascular disease risk factors: age, sex, smoking, high-density lipoprotein, total cholesterol, systolic blood pressure, diabetes, hypertension medication.

CI = confidence interval. HDL = high-density lipoprotein. SBP = systolic blood pressure. CV = cardiovascular.

There were < 5% missing values for descriptive variables such as BMI and smoking status. Missing values were addressed using multiple imputations using chained equations.

Figure 2. Aortic stiffness at baseline, their hazard ratios and discriminative ability for fatal or non-fatal hospitalised cardiovascular disease.

Values are given as the median and interquartile range (IQR), or No. of cases (%). Baseline data of 714 patients are included. All models are stratified by recruitment site.

Goodness of fit (chi2(3), P > chi2): CV risk model (2.07, 0.559), aPWV (1.64, 0.652), CIMT (2.32, 0.509), AIx (3.08, 0.380). Estimates based on quartiles of risk.

Brier score (95% CI): CV risk model 0.129 (0.111 to 0.146), aPWV 0.126 (0.108 to 0.145), CIMT 0.128 (0.110 to 0.147), AIx 0.126 (0.109 to 0.144). Lower score indicates better accuracy of estimates.

[a]Model includes age and sex.

[b]Model includes conventional cardiovascular disease risk factors: age, sex, smoking, high-density lipoprotein, total cholesterol, systolic blood pressure, diabetes, hypertension medication. Carotid intima-media thickness further included systolic blood pressure. Carotid-femoral aortic pulse wave velocity further included mean arterial pressure and resting heart rate. AIx further included resting heart rate and height.

CI = confidence interval. PWV = pulse wave velocity. CIMT = carotid intima-media thickness. AIx = augmentation index.

For peer review only

There were about 10% missing values for variables CIMT (n = 66) and aPWV (n = 60). Missing values were addressed using multiple imputations using chained equations.

Figure 3. Alternative measures at baseline, their hazard ratios and discriminative ability for fatal or non-fatal hospitalised cardiovascular disease.

Values are given as the median and interquartile range (IQR), or No. of cases (%). Baseline data of 714 patients are included. All models are stratified by recruitment site.

Goodness of fit (chi2(3), P > chi2): CV risk model (2.07, 0.559), CRP (0.32, 0.956), Fibrinogen (1.48, 0.687), Glucose (0.42, 0.936), BMI (1.56, 0.668), GOLD (5.63, 0.131), 4MGS (4.70, 0.195), 6MW (2.94, 0.401), BODE (6.46, 0.091), BMI, 4MGS, 6MW, BODE (4.12, 0.249). Estimates based on quartiles of risk.

Brier score (95% CI): CV risk model 0.129 (0.111 to 0.146), CRP 0.125 (0.107 to 0.1423), Fibrinogen 0.128 (0.111 to 0.146), Glucose 0.128 (0.111 to 0.146), BMI 0.128 (0.111 to 0.146), GOLD 0.128 (0.110 to 0.146), 4MGS 0.127 (0.110 to 0.144), 6MW 0.123 (0.105 to 0.140), BODE 0.124 (0.106 to 0.142), BMI, 4MGS, 6MW, BODE 0.122 (0.104 to 0.140). Lower score indicates better accuracy of estimates.

[a]Model includes age and sex.

[b]Model includes conventional cardiovascular disease risk factors: age, sex, smoking, high-density lipoprotein, total cholesterol, systolic blood pressure, diabetes, hypertension medication.

CI = confidence interval. CRP = C-reactive protein. BMI = body mass index. GOLD = global initiative for chronic obstructive lung disease. 4MGS = four-metre gait speed. 6MWT = six-minute walk test. BODE = body mass index, obstruction, dyspnoea, exercise.

There were < 5% missing values for biochemical markers including fibrinogen and cholesterol. Missing values were addressed using multiple imputations using chained equations.

For peer review only

Online supplementary material

Supplementary text 1 Supplementary methods.

Supplementary table 1 Definitions of diagnoses by ICD-10 coding.

Supplementary table 2 Fatal or non-fatal hospitalised cardiovascular disease by ICD-10 coding position.

Supplementary table 3 Conventional cardiovascular disease risk factors at baseline, their hazard ratios for cardiovascular disease.

Supplementary table 4 Arterial stiffness at baseline, their hazard ratios for cardiovascular disease.

Supplementary table 5 Alternative measures at baseline, their hazard ratios for cardiovascular disease.

Supplementary figure 1 Percentage of missing values.

Supplementary figure 2 Percentage and pattern of missing values in key baseline characteristics.

Supplementary figure 3 Differences in baseline pulse wave velocity.

Supplementary figure 4 Differences in baseline carotid intima-media thickness.

Supplementary figure 5 Differences in baseline augmentation index.

Supplementary figure 6 Participant enrolment flow diagram with five years follow up.

For peer review only

Risk factor	Median (IQR) or n (%)	HR (95% CI)[a]	HR (95% CI)[b]	C-statistic
All conventional CVD risk factors				0.689 [0.688 to 0.691]
Age – per 10 year increase	67 (62–73)	1.58 (1.30 to 1.92)	1.59 (1.29 to 1.99)	0.619 [0.618 to 0.621]
Sex – male	434 (61)	1.28 (0.94 to 1.74)	1.16 (0.84 to 1.60)	0.585 [0.581 to 0.588]
Smoking – current	218 (31)	0.95 (0.68 to 1.31)	0.92 (0.66 to 1.29)	0.515 [0.512 to 0.517]
HDL – per 1 mmol/L increase	1.4 (1.2–1.7)	0.73 (0.52 to 1.03)	0.86 (0.60 to 1.23)	0.571 [0.569 to 0.573]
Cholesterol – per 1 mmol/L increase	5.0 (4.3–5.8)	0.80 (0.70 to 0.92)	0.89 (0.76 to 1.03)	0.613 [0.611 to 0.614]
SBP – per 10 mmHg increase	142 (131–154)	0.91 (0.84 to 0.99)	0.90 (0.82 to 0.98)	0.532 [0.530 to 0.535]
Diabetes – yes	82 (12)	1.50 (1.01 to 2.22)	1.15 (0.76 to 1.75)	0.538 [0.534 to 0.539]
Hypertension medication – yes	245 (34)	1.79 (1.34 to 2.38)	1.76 (1.31 to 2.36)	0.620 [0.616 to 0.623]

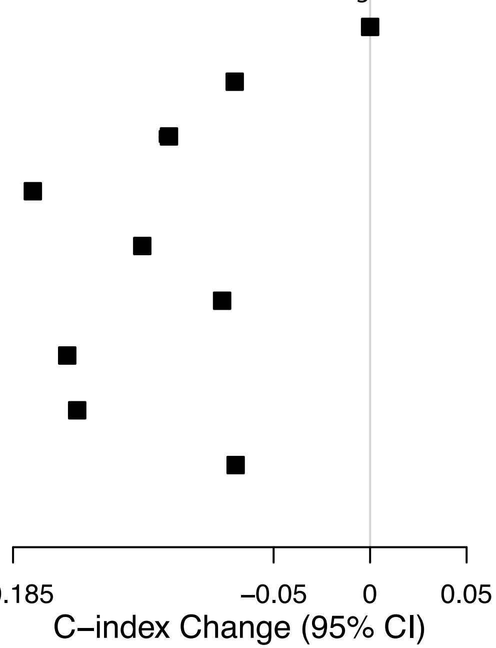

For peer review only

Risk factor	Median (IQR) or n (%)	HR (95% CI)[a]	HR (95% CI)[b]	C−statistic
All conventional CVD risk factors				0.689 [0.688 to 0.691]
+PWV – per 1 m/sec increase	9.8 (8.4–11.8)	1.01 (0.95 to 1.07)	0.99 (0.93 to 1.06)	0.691 [0.689 to 0.692]
+CIMT – per 1 mm increase	0.81 (0.71–0.96)	1.09 (0.49 to 2.45)	1.08 (0.48 to 2.43)	0.691 [0.690 to 0.693]
+AIx – per 5% increase	28 (20–34)	0.85 (0.77 to 0.93)	0.90 (0.82 to 0.99)	0.688 [0.686 to 0.689]

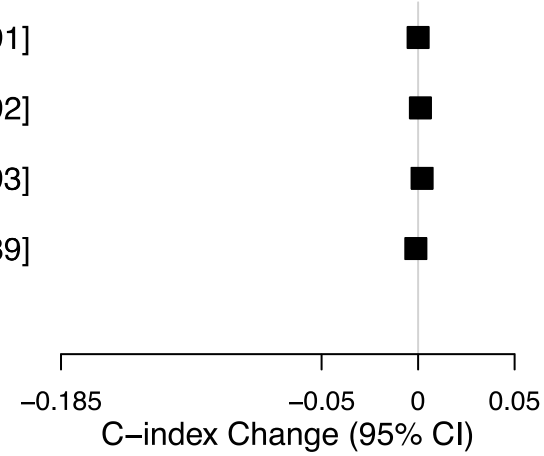

For peer review only

Risk factor	Median (IQR) or n (%)	HR (95% CI)[a]	HR (95% CI)[b]	C−statistic
All conventional CVD risk factors				0.689 [0.688 to 0.691]
+ CRP – per twofold increase	1.21 (0.47 to 2.01)	1.22 (1.07 to 1.39)	1.20 (1.06 to 1.37)	0.693 [0.691 to 0.694]
+ Fibrinogen – per twofold increase	1.22 (1.06 to 1.36)	2.27 (1.20 to 4.31)	2.12 (1.10 to 4.07)	0.693 [0.691 to 0.694]
+ Glucose – per twofold increase	1.59 (1.46 to 4.59)	1.18 (0.46 to 2.99)	0.83 (0.32 to 2.18)	0.690 [0.688 to 0.691]
+ BMI – per 1 kg/m2 increase	27 (23–31)	1.04 (1.01 to 1.06)	1.02 (0.99 to 1.05)	0.698 [0.696 to 0.699]
+ GOLD – per 1 stage increase	1 (1–2)	1.22 (0.98 to 1.52)	1.29 (1.03 to 1.61)	0.689 [0.687 to 0.691]
+ 4MGS – per 1 second increase	4.2 (3.5 to 5.2)	1.09 (1.03 to 1.16)	1.07 (1.01 to 1.14)	0.712 [0.710 to 0.713]
+ 6MW distance – per 30 meter increase	366 (255 to 440)	0.91 (0.88 to 0.94)	0.91 (0.88 to 0.95)	0.727 [0.726 to 0.728]
+ BODE – per 1 point increase	3 (1–5)	1.13 (1.07 to 1.20)	1.15 (1.08 to 1.21)	0.705 [0.704 to 0.707]
+ BMI, 4MGS, 6MW, BODE – per 1 unit increase				0.731 [0.729 to 0.732]

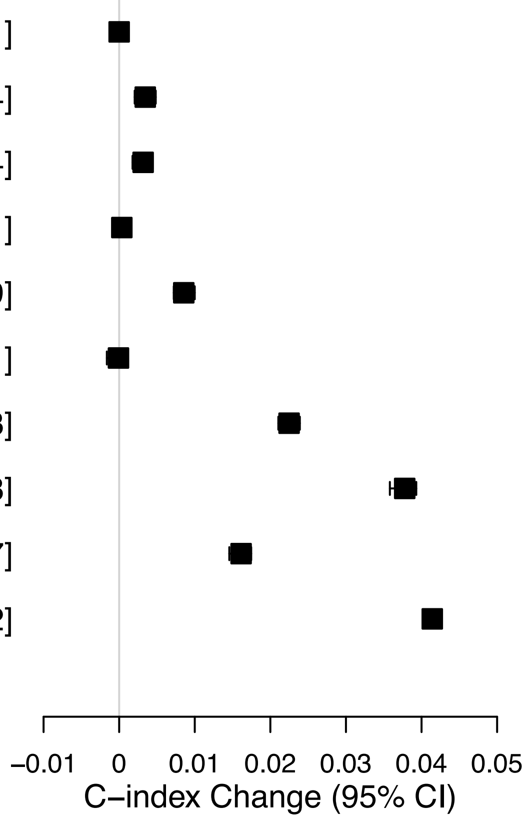

For peer review only

Supplementary materials for "Cardiovascular risk prediction using physical performance measures in COPD: results from a multi-centre observational study."

Jilles M. Fermont, Marie Fisk, Charlotte E. Bolton, William Macnee, John Cockcroft, Jonathan Fuld, Joseph Cheriyan, Divya Mohan, Kaisa M. Mäki-Petäjä, Ali B.A.K. Al-Hadithi, Ruth Tal-Singer, Hana Müllerova, Michael I. Polkey, Angela M. Wood, Carmel McEniery, Ian B. Wilkinson, on behalf of the ERICA consortium.

For peer review only

Supplementary text

Text 1 Supplementary methods

There were missing values. Data were assessed for the level and type of missing data, and completion patterns (Figures 1-2). There were about 10% missing values for variables CIMT (n = 66) and PWV (n = 60), with <5% missing values for other variables (Figures 3-5). Missing values were addressed using multiple imputations using chained equations (MICE). The time-to-event outcome was included using the non-parametric Nelson-Aalen estimator. Predictive mean matching was used for continuous variables, ordered logistic regression (as continuous) for ordinal variables, multinomial logistic regression for categorical variables, and logistic regression for binary variables. Derived variables such as the BODE Index (a composite score of BMI, forced expiratory volume in one second (FEV_1), Medical Research Council dyspnoea score, and 6MWT distance) and GOLD stage were estimated post MICE using passive imputation.

Data were cleaned for episode status, and inpatient (i.e. hospitalised) CV episodes were identified based on classifications used by the Emerging Risk Factors Collaboration.[1] Cardiovascular events were extracted from both primary and secondary positions of ICD-10. Priority was given to CV events reported in the primary position (n = 70). Following this, we selected CV events in the secondary position (n = 116), which had no recording of a CV event in the primary position. Only episodes during the study follow-up were evaluated.

[1] The Emerging Risk Factors Collaboration: analysis of individual data on lipid, inflammatory and other markers in over 1.1 million participants in 104 prospective studies of cardiovascular diseases. *Eur J Epidemiol* **22,** 839–869 (2007). https://doi.org/10.1007/s10654-007-9165-7

For peer review only

Supplementary tables

Table 1 Definitions of diagnoses by ICD-10 coding.

End point	ICD-10 codes
**All cardiovascular disease**	**F01, G46.3-G46.7, G458, G459 I11.0, I13.0, I13.2, I20.0-I20.1, I20.8-I25, I50, I60, I61, I.62, I63, I64, I65-I69, I70.2, I71.3-I71.9, I72, I73.9-I79 + cardiac death**
**Diseases of the arteries**	**I70.2, I72, I73.9-I79**
Peripheral arterial disease	I70.2, I73.9
Diseases of arteries, arterioles and capillaries	I72, I74-I79
**Coronary heart disease**	**I20.0-I20.1, I20.8-I25**
Angina	I20.1, I20.8-I20.9
Unstable angina	I20.0, I24
Coronary heart disease not otherwise specified	I25
Acute myocardial infarction (MI), and certain current complications following acute MI	I21, I23
Subsequent myocardial infarction	I22
**All stroke**	**I60, I61, I.62, I63, I64, I65-I69, F01, G46.3-G46.7, G458, G459**
Subarachnoid haemorrhage	I60
Intra-cerebral haemorrhage	I61
Cerebral infarction	I63
Stroke, not specified as haemorrhage or infarction	I64
Stroke syndromes	G46.3-G46.7
Transient ischemic attack	G458, G459
Other stroke	I62, I65-I69, F01
**Heart failure**	**I11.0, I13.0, I13.2, I50**
Heart failure	I50
Hypertensive heart disease with (congestive) heart failure	I11.0
Hypertensive heart and renal disease with (congestive) heart failure	I13.0
Hypertensive heart and renal disease with both (congestive) heart failure and renal disease	I13.2
**Cardiac death**	**Adjudicated**
**Other vascular deaths**	**I71.3-I71.9**
Abdominal aortic aneurysm	I71.3-I71.9

For peer review only

Table 2 Fatal or non-fatal hospitalised cardiovascular disease by ICD-10 coding position.

	All cardiovascular disease			AECOPD	
	**Primary ICD-10 position**	**Secondary ICD-10 position**	**Cardiac death**	**Primary ICD-10 position**	**Secondary ICD-10 position**
1	I714			J441	
2	I209			J441	
3	I501				
4	I771			J440	
5	I251				
6	I251				
7	I219				J440
8	I251				
9	I745				
10	I259				
11	I251			J441	
12	I251				
13	I500			J440	
14	I743			J441	
15	I64X			J440	
16	I714				
17	I219			J440	
18	I211				
19	I64X				
20	I739				
21	I739				
22	I639				
23	I251			J441	
24	I652				
25	I251			J440	
26	I251				
27	I632				
28	I251			J440	
29	I660			J440	
30	I500			J441	
31	I771				
32	I251			J441	
33	I638			J441	
34	I702			J440	
35	I200			J441	
36	I652				
37	I501			J441	
38	I214				
39	I64X			J440	
40	I509				
41	I739				
42	I739				
43	I639				J440
44	I743				
45	I251				
46	I251			J440	
47	I219				
48	I638				
49	I251			J440	
50	I251			J440	
51	I639				J440

For peer review only

52	I500				
53	I251				
54	I251			J441	
55	I639				
56	I652				J440
57	I219				
58	I635				
59	I214			J440	
60	I251				
61	I739				J440
62	I639				
63	I251			J440	
64	I200				J440
65	I214				J440
66	I251				
67	I634				J440
68	I209				J440
69	I714				
70	I509				
71		I209		J440	
72		I501			
73		I713			J440
74		I714			
75		I252			J440
76		I259		J440	
77		I252		J440	
78		I500		J441	
79		I694			
80		I209			
81		I209			
82		I259		J441	
83		I509		J440	
84		I500			J440
85		I509			J440
86		I200			
87		I219		J440	
88		I714			J440
89		I780			J440
90		I219		J440	
91		I252		J440	
92		I714		J441	
93		I209			J440
94		I251		J440	
95		I259			J440
96		I501		J440	
97		I252			
98		I252			
99		I500			J440
100		I252			J440
101		I252			
102		I652			
103		I500		J440	
104		I259		J441	
105		I251			
106		I501		J440	
107		I252			
108		I255			J440
109		I259			J440

110		I500		J440	
111		I252			
112		I252		J440	
113		I259			
114		I678		J441	
115		I501			J440
116		I678		J440	
117		I501			J440
118		I678		J440	
119		I259			
120		I209			
121		I209		J441	
122		I500		J441	
123		I259			J440
124		I259			J440
125		I259			J440
126		I209		J440	
127		I252			J440
128		I509		J440	
129		I209			
130		I259			
131		I259			
132		I259		J440	
133		I209			
134		I679			
135		I678		J440	
136		I500		J440	
137		I501			
138		I500		J441	
139		I209			
140		I652		J440	
141		I209			
142		I678			
143		I209			
144		I209		J441	
145		I501		J441	
146		I209		J440	
147		I209		J440	
148		I739			
149		I509			
150		I678		J441	
151		I252		J440	
152		I252			
153		I501		J440	
154		I678			
155		I251		J440	
156		I252			J440
157		I500		J440	
158		I252		J440	
159		I252		J440	
160		I634			
161		I679			
162		I252		J440	
163		I252			
164		I500			J440
165		I209		J441	
166		I739			J440
167		I739		J440	

For peer review only

168		I259			
169		I509			J440
170		I252			
171		I209			J440
172		I798		J440	
173		I500			
174		I209			
175		I501		J440	
176		I219		J441	
177		I209			
178		I252		J440	
179		I252			
180		I671		J441	
181		I259			
182		I259		J440	
183		I671			
184		I259			J440
185		I251			J440
186		I714			J440
187			Cardiac		
188			Cardiac		
189			Cardiac		
190			Cardiac		
191			Cardiac		
192			Cardiac		
Total recorded	70	116	6	74	35

Twenty-five individuals with a CV event recorded in the primary position also had an acute exacerbation of COPD (AECOPD) recorded in the primary position during the study period. In addition, 49 individuals with a CV event recorded in the secondary position also had an AECOPD recorded in the primary position during the study period.

For peer review only

Table 3 Conventional cardiovascular disease risk factors at baseline, their hazard ratios for cardiovascular disease.

	Median (IQR) or No. (%)	HR (95% CI)[a]	P value	HR (95% CI)[b]	P value
**Conventional CVD risk factors**					
Age – per 10 year increase	67 (62-73)	1.58 (1.30 to 1.92)	< 0.001	1.59 (1.29 to 1.99)	< 0.001
Sex – males	434 (61)	1.28 (0.94 to 1.74)	0.113	1.16 (0.84 to 1.60)	0.375
Smoking – current	218 (31)	0.95 (0.68 to 1.31)	0.744	0.92 (0.66 to 1.29)	0.632
HDL – per 1 mmol/L increase	1.4 (1.2-1.7)	0.73 (0.52 to 1.03)	0.074	0.86 (0.60 to 1.23)	0.402
Total cholesterol – per 1 mmol/L increase	5.0 (4.3-5.8)	0.80 (0.70 to 0.92)	0.002	0.89 (0.76 to 1.03)	0.118
SBP – per 10 mmHg increase	142 (131-154)	0.91 (0.84 to 0.99)	0.023	0.90 (0.82 to 0.98)	0.016
Diabetes – yes	82 (12)	1.50 (1.01 to 2.22)	0.044	1.15 (0.76 to 1.75)	0.511
Hypertension treatment – yes	245 (34)	1.79 (1.34 to 2.38)	< 0.001	1.76 (1.31 to 2.36)	< 0.001

Values are given as the median and interquartile range (IQR), or No. of cases (%). Baseline data of 714 patients are included. All models are stratified by recruitment site.
[a]Model includes age and sex.
[b]Model includes conventional cardiovascular disease risk factors: age, sex, smoking, high-density lipoprotein, total cholesterol, systolic blood pressure, diabetes, hypertension drug treatment.
CI = confidence interval. HDL = high-density lipoprotein. SBP = systolic blood pressure. CV = cardiovascular.

For peer review only

Table 4 Arterial stiffness at baseline, their hazard ratios for cardiovascular disease.

	Median (IQR) or No. (%)	HR (95% CI)[a]	P value	HR (95% CI)[b]	P value
**Measures of arterial stiffness**					
PWV – per 1 m/sec increase	9.8 (8.4-11.8)	1.01 (0.95 to 1.07)	0.796	0.99 (0.93 to 1.06)	0.749
CIMT – per 1 mm increase	0.81 (0.71-0.96)	1.09 (0.49 to 2.45)	0.827	1.08 (0.48 to 2.43)	0.861
AIx – per 5% increase	28 (20-34)	0.85 (0.77 to 0.93)	< 0.001	0.90 (0.82 to 0.99)	0.027

Values are given as the median and interquartile range (IQR), or No. of cases (%). Baseline data of 714 patients are included. All models are stratified by recruitment site.
[a]Model includes age and sex.
[b]Model includes conventional cardiovascular disease risk factors: age, sex, smoking, high-density lipoprotein, total cholesterol, systolic blood pressure, diabetes, hypertension drug treatment. Carotid intima-media thickness further included systolic blood pressure. Carotid-femoral aortic pulse wave velocity further included mean arterial pressure and resting heart rate. AIx further included resting heart rate and height.
CI = confidence interval. PWV = pulse wave velocity. CIMT = carotid intima-media thickness. AIx = augmentation index.

For peer review only

Table 5 Alternative measures at baseline, their hazard ratios for cardiovascular disease.

	Median (IQR) or No. (%)	HR (95% CI)[a]	P value	HR (95% CI)[b]	P value
**Other risk factors**					
CRP – per twofold increase	1.21 (0.47 to 2.01)	1.22 (1.07 to 1.39)	0.002	1.20 (1.06 to 1.37)	0.005
Fibrinogen – per twofold increase	1.22 (1.06 to 1.36)	2.27 (1.20 to 4.31)	0.012	2.12 (1.10 to 4.07)	0.024
Glucose – per twofold increase	1.59 (1.50 to 1.69)	1.18 (0.46 to 2.99)	0.730	0.83 (0.32 to 2.18)	0.709
BMI – per 1 kg/m^2 increase	27 (23-31)	1.04 (1.01 to 1.06)	0.002	1.02 (0.99 to 1.05)	0.089
GOLD – per 1 stage increase	1 (1-2)	1.22 (0.98 to 1.52)	0.071	1.29 (1.03 to 1.61)	0.026
4MGST – per 1 second increase	4.2 (3.5 to 5.2)	1.09 (1.03 to 1.16)	0.002	1.07 (1.01 to 1.14)	0.020
6MWT distance – per 30 metre increase	366 (255 to 440)	0.91 (0.88 to 0.94)	< 0.001	0.91 (0.88 to 0.95)	< 0.001
BODE – per 1 point increase	3 (1-5)	1.13 (1.07 to 1.20)	< 0.001	1.15 (1.08 to 1.21)	< 0.001

Values are given as the median and interquartile range (IQR), or No. of cases (%). Baseline data of 714 patients are included. All models are stratified by recruitment site.
[a]Model includes age and sex.
[b]Model includes conventional cardiovascular disease risk factors: age, sex, smoking, high-density lipoprotein, total cholesterol, systolic blood pressure, diabetes, hypertension drug treatment.
CI = confidence interval. CRP = C-reactive protein. BMI = body mass index. GOLD = global initiative for chronic obstructive lung disease. 4MGS = four-metre gait speed. 6MWT = six-minute walk test. BODE = body mass index, obstruction, dyspnoea, exercise.

For peer review only

Supplementary figures

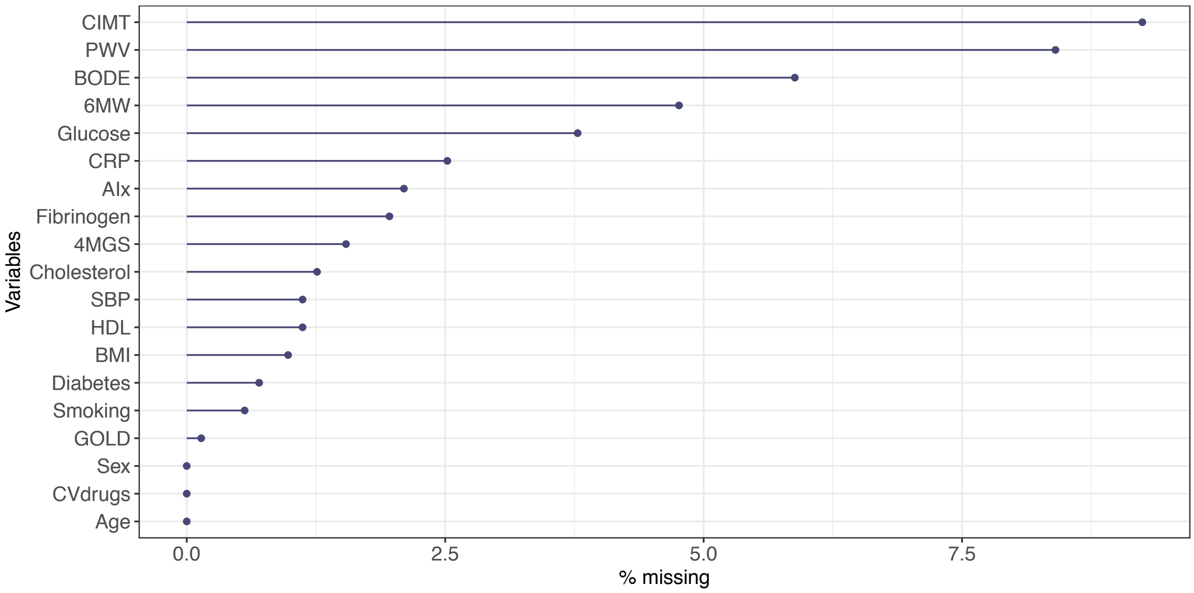

Figure 1 Percentage of missing values.
CIMT = carotid intima-media thickness. PWV = pulse wave velocity. BODE = body mass index, obstruction (i.e. forced expiratory volume in one second), dyspnoea score, exercise (i.e. six-minute walk test distance). 6MWT = six-minute walk test. CRP = C-reactive protein. AIx = augmentation index. 4MGS = four-metre gait speed. SBP = systolic blood pressure. HDL = high-density lipoprotein. BMI = body mass index. GOLD = global initiative for obstructive lung disease. CV = cardiovascular.

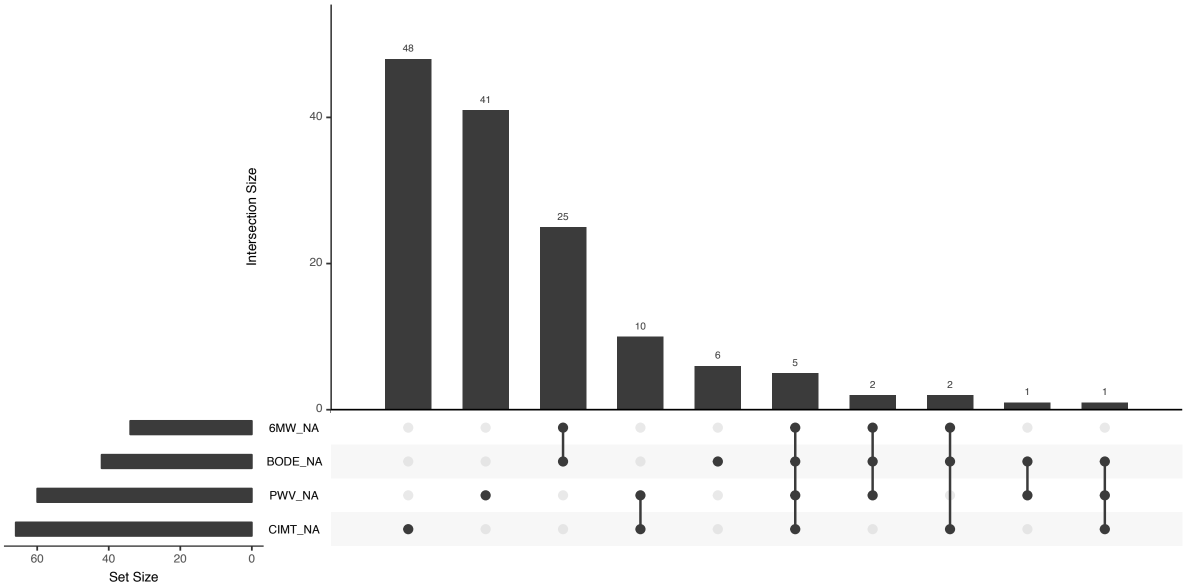

Figure 2 Percentage and pattern of missing values in key baseline characteristics.
6MWT, six-minute walk test. BODE = body mass index, obstruction (i.e. forced expiratory volume in one second), dyspnoea score, exercise (i.e. six-minute walk test distance). PWV, pulse wave velocity. CIMT, carotid-intima media thickness.

For peer review only - http://bmjopen.bmj.com/site/about/guidelines.xhtml

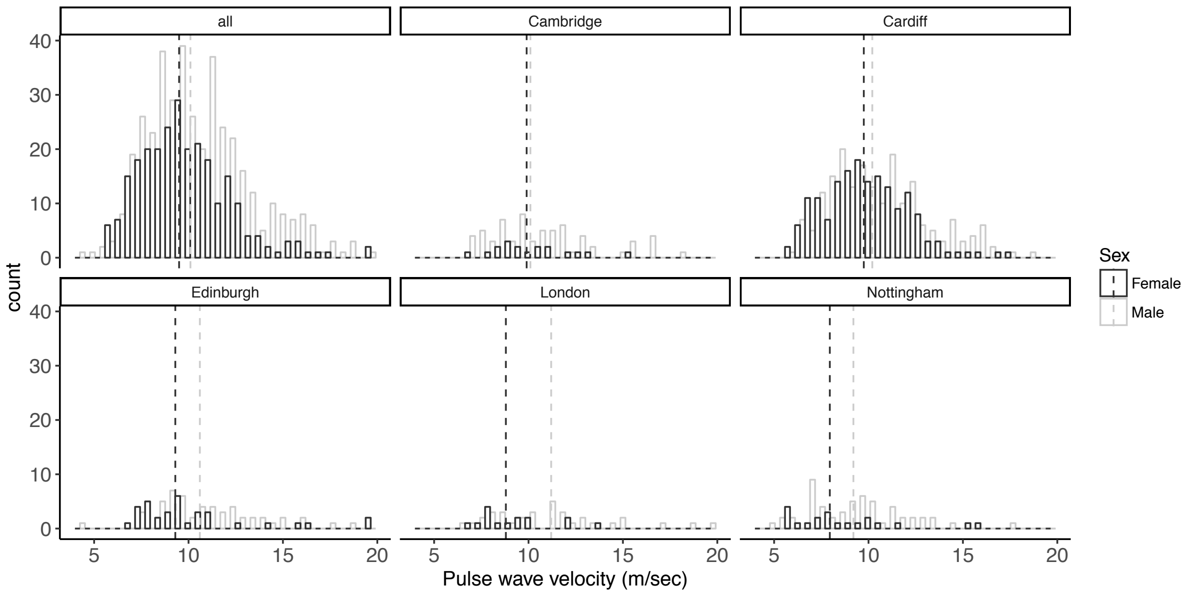

Figure 3 Differences in baseline pulse wave velocity.
Histograms displaying the distribution of PWV by sex and recruitment site. Dashed lines indicate median values by sex. Where only one median line is visible, medians for both sexes are similar.

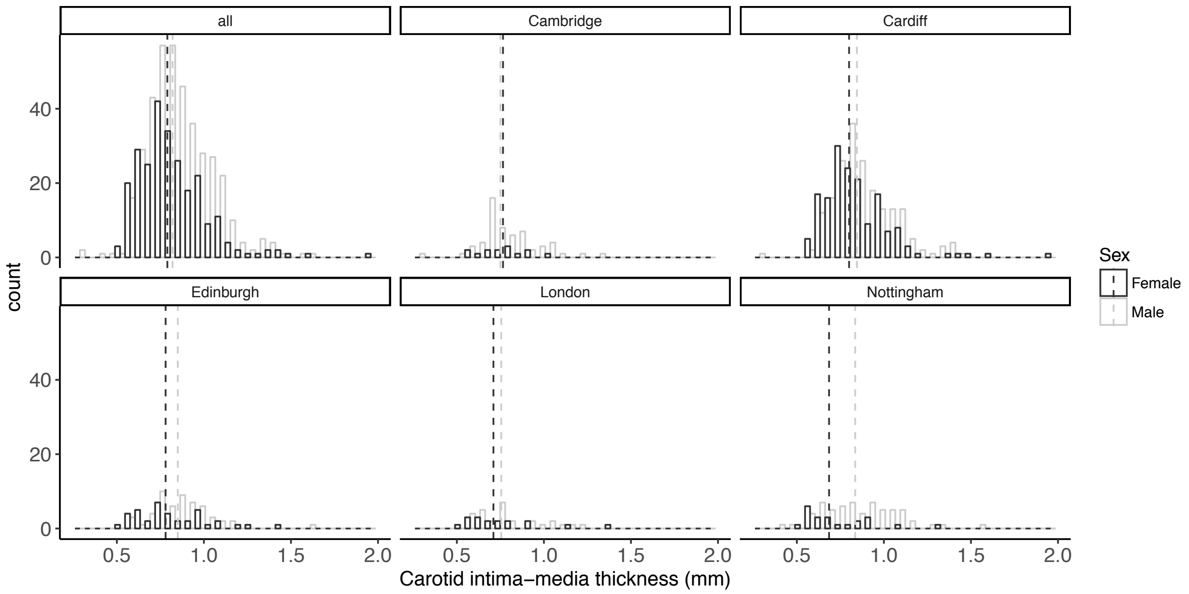

Figure 4 Differences in baseline carotid intima-media thickness.
Histograms displaying the distribution of CIMT by sex and recruitment site. Dashed lines indicate median values by sex. Where only one median line is visible, medians for both sexes are similar.

For peer review only - http://bmjopen.bmj.com/site/about/guidelines.xhtml

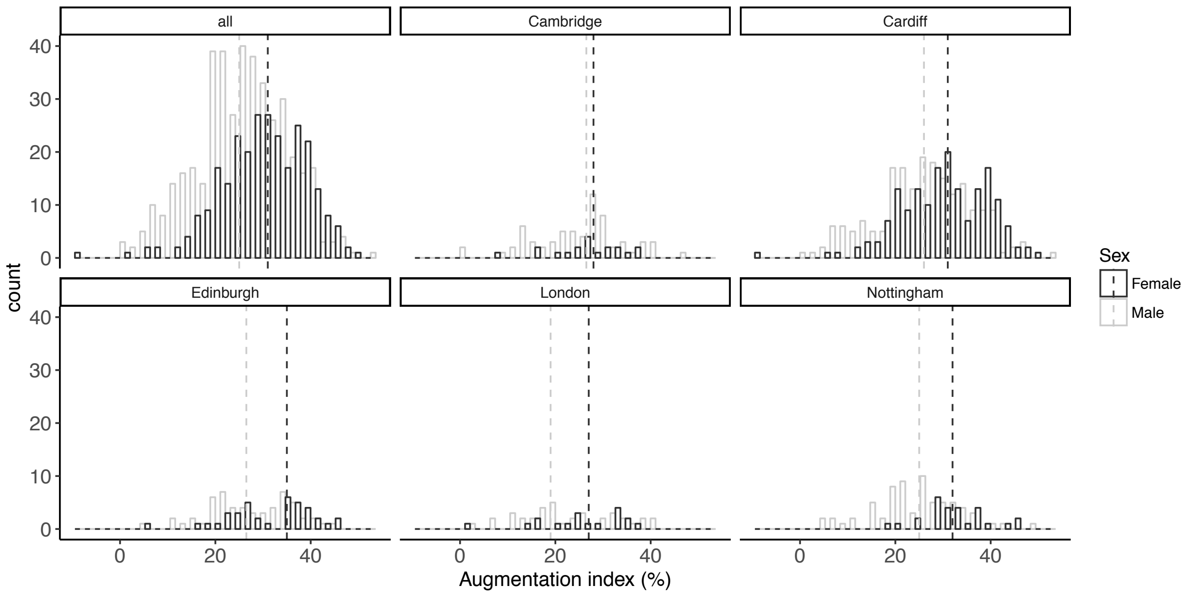

Figure 5 Differences in baseline augmentation index.
Histograms displaying the distribution of AIx by sex and recruitment site. Dashed lines indicate median values by sex.

For peer review only

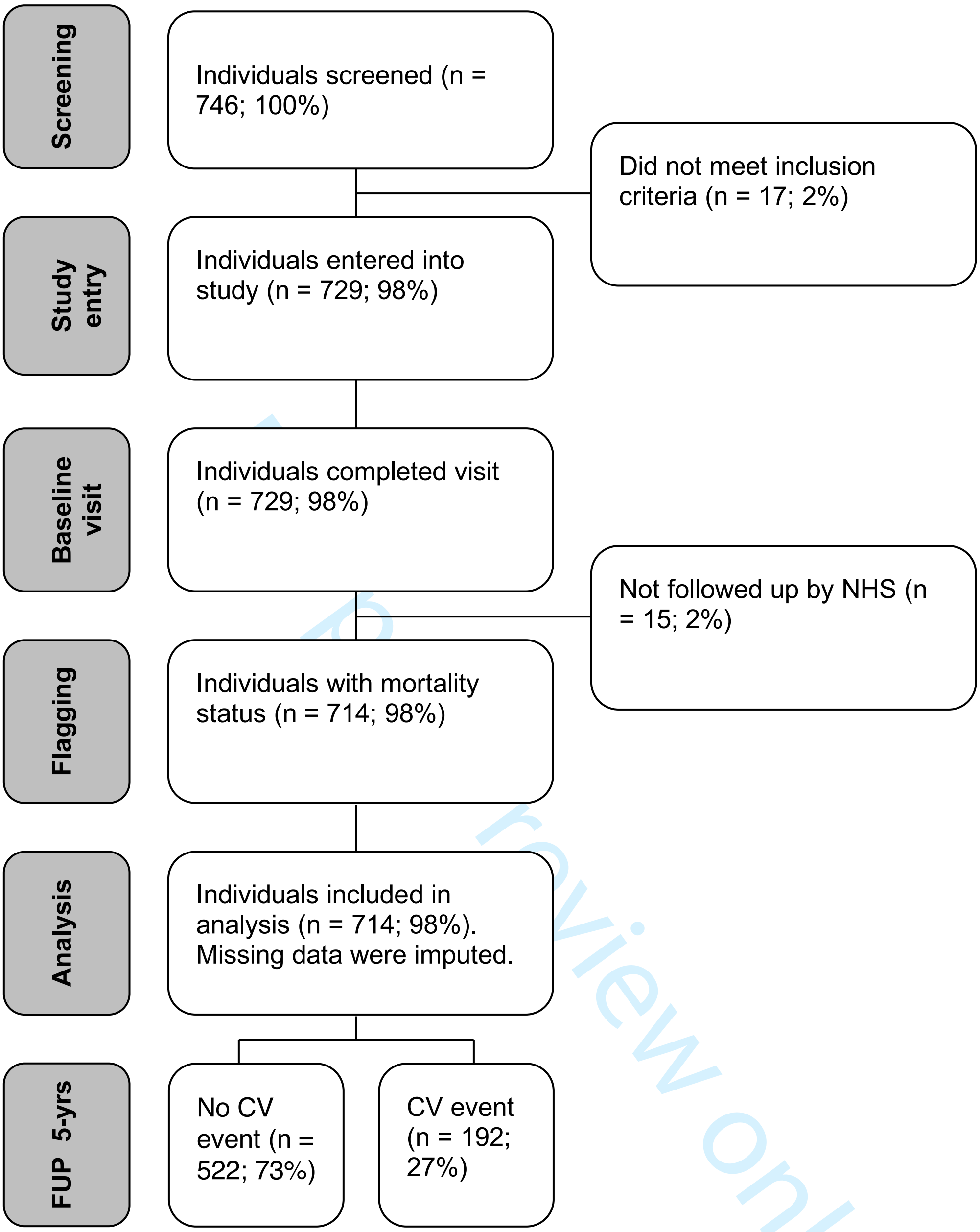

Figure 6 Participant enrolment flow diagram with five years follow up.
Total number of CV events (n = 192): peripheral arterial disease (n = 9), diseases of arteries, arterioles and capillaries (n = 7), angina (n = 21), unstable angina (n = 3), coronary heart disease not otherwise specified (n = 63), acute myocardial infarction (MI), and certain current complications following acute MI (n = 11), cerebral infarction (n = 11), stroke, not specified as haemorrhage or infarction (n = 3), other stroke (n = 18), heart failure (n = 32), abdominal aortic aneurysm (n = 8), and cardiac death (n = 6). FEV_1 = forced expiratory volume one second. FVC = forced vital capacity. NHS = National Health Services. FUP = follow-up period.

For peer review only - http://bmjopen.bmj.com/site/about/guidelines.xhtml

STROBE 2007 (v4) Statement—Checklist of items that should be included in reports of *cohort studies*

Section/Topic	Item #	Recommendation	Reported on page #
**Title and abstract**	1	(*a*) Indicate the study's design with a commonly used term in the title or the abstract	1
		(*b*) Provide in the abstract an informative and balanced summary of what was done and what was found	3
**Introduction**			
Background/rationale	2	Explain the scientific background and rationale for the investigation being reported	6
Objectives	3	State specific objectives, including any prespecified hypotheses	7
**Methods**			
Study design	4	Present key elements of study design early in the paper	8
Setting	5	Describe the setting, locations, and relevant dates, including periods of recruitment, exposure, follow-up, and data collection	8
Participants	6	(*a*) Give the eligibility criteria, and the sources and methods of selection of participants. Describe methods of follow-up	8
		(*b*) For matched studies, give matching criteria and number of exposed and unexposed	
Variables	7	Clearly define all outcomes, exposures, predictors, potential confounders, and effect modifiers. Give diagnostic criteria, if applicable	8-9
Data sources/ measurement	8*	For each variable of interest, give sources of data and details of methods of assessment (measurement). Describe comparability of assessment methods if there is more than one group	8-9
Bias	9	Describe any efforts to address potential sources of bias	9
Study size	10	Explain how the study size was arrived at	8-9
Quantitative variables	11	Explain how quantitative variables were handled in the analyses. If applicable, describe which groupings were chosen and why	9
Statistical methods	12	(*a*) Describe all statistical methods, including those used to control for confounding	9 + supplementary text
		(*b*) Describe any methods used to examine subgroups and interactions	9
		(*c*) Explain how missing data were addressed	Supplementary text 1
		(*d*) If applicable, explain how loss to follow-up was addressed	

For peer review only

		(*e*) Describe any sensitivity analyses	
**Results**			
Participants	13*	(a) Report numbers of individuals at each stage of study—eg numbers potentially eligible, examined for eligibility, confirmed eligible, included in the study, completing follow-up, and analysed	11 + supplementary figure 6
		(b) Give reasons for non-participation at each stage	Supplementary figure 6
		(c) Consider use of a flow diagram	Supplementary figure 6
Descriptive data	14*	(a) Give characteristics of study participants (eg demographic, clinical, social) and information on exposures and potential confounders	11-12
		(b) Indicate number of participants with missing data for each variable of interest	Supplementary figures 1-5
		(c) Summarise follow-up time (eg, average and total amount)	11
Outcome data	15*	Report numbers of outcome events or summary measures over time	11
Main results	16	(*a*) Give unadjusted estimates and, if applicable, confounder-adjusted estimates and their precision (eg, 95% confidence interval). Make clear which confounders were adjusted for and why they were included	12-14
		(*b*) Report category boundaries when continuous variables were categorized	
		(*c*) If relevant, consider translating estimates of relative risk into absolute risk for a meaningful time period	
Other analyses	17	Report other analyses done—eg analyses of subgroups and interactions, and sensitivity analyses	
**Discussion**			
Key results	18	Summarise key results with reference to study objectives	15
**Limitations**			
Interpretation	20	Give a cautious overall interpretation of results considering objectives, limitations, multiplicity of analyses, results from similar studies, and other relevant evidence	16-18
Generalisability	21	Discuss the generalisability (external validity) of the study results	18
**Other information**			
Funding	22	Give the source of funding and the role of the funders for the present study and, if applicable, for the original study on which the present article is based	19-20

*Give information separately for cases and controls in case-control studies and, if applicable, for exposed and unexposed groups in cohort and cross-sectional studies.

For peer review only

Note: An Explanation and Elaboration article discusses each checklist item and gives methodological background and published examples of transparent reporting. The STROBE checklist is best used in conjunction with this article (freely available on the Web sites of PLoS Medicine at http://www.plosmedicine.org/, Annals of Internal Medicine at http://www.annals.org/, and Epidemiology at http://www.epidem.com/). Information on the STROBE Initiative is available at www.strobe-statement.org.

For peer review only

BMJ Open

Cardiovascular risk prediction using physical performance measures in COPD: results from a multi-centre observational study.

Journal:	*BMJ Open*
Manuscript ID	bmjopen-2020-038360.R3
Article Type:	Original research
Date Submitted by the Author:	17-Nov-2020
Complete List of Authors:	Fermont, Jilles; University of Cambridge, Department of Medicine<br>Fisk, Marie; University of Cambridge<br>Bolton, Charlotte; University of Nottingham<br>MacNee, William; The University of Edinburgh<br>Cockcroft, John; Columbia University Medical Center<br>Fuld, Jonathan; Cambridge University Hospitals NHS Foundation Trust, Centre for Self Management Support<br>Cheriyan, Joseph; University of Cambridge Medicine, Division of Experimental Medicine & Immunotherapeutics<br>Mohan, Divya; GSK R&D<br>Mäki-Petäjä, Kaisa ; University of Cambridge<br>Al-Hadithi, Ali ; University of Cambridge,<br>Tal-Singer, Ruth; GSK R&D<br>Müllerova, Hana; GSK R&D<br>Polkey, Mike; Royal Brompton Hospital<br>Wood, Angela; University of Cambridge<br>McEniery, Carmel M.; University of Cambridge<br>Wilkinson, Ian; University of Cambridge; Cambridge Clinical Trials Unit,
**Primary Subject Heading**:	Respiratory medicine
Secondary Subject Heading:	Cardiovascular medicine, Epidemiology, Diagnostics, Public health
Keywords:	Chronic airways disease < THORACIC MEDICINE, EPIDEMIOLOGY, RESPIRATORY MEDICINE (see Thoracic Medicine), Cardiac Epidemiology < CARDIOLOGY, PRIMARY CARE

SCHOLARONE™
Manuscripts

I, the Submitting Author has the right to grant and does grant on behalf of all authors of the Work (as defined in the below author licence), an exclusive licence and/or a non-exclusive licence for contributions from authors who are: i) UK Crown employees; ii) where BMJ has agreed a CC-BY licence shall apply, and/or iii) in accordance with the terms applicable for US Federal Government officers or employees acting as part of their official duties; on a worldwide, perpetual, irrevocable, royalty-free basis to BMJ Publishing Group Ltd ("BMJ") its licensees and where the relevant Journal is co-owned by BMJ to the co-owners of the Journal, to publish the Work in this journal and any other BMJ products and to exploit all rights, as set out in our licence.

The Submitting Author accepts and understands that any supply made under these terms is made by BMJ to the Submitting Author unless you are acting as an employee on behalf of your employer or a postgraduate student of an affiliated institution which is paying any applicable article publishing charge ("APC") for Open Access articles. Where the Submitting Author wishes to make the Work available on an Open Access basis (and intends to pay the relevant APC), the terms of reuse of such Open Access shall be governed by a Creative Commons licence – details of these licences and which Creative Commons licence will apply to this Work are set out in our licence referred to above.

Other than as permitted in any relevant BMJ Author's Self Archiving Policies, I confirm this Work has not been accepted for publication elsewhere, is not being considered for publication elsewhere and does not duplicate material already published. I confirm all authors consent to publication of this Work and authorise the granting of this licence.

For peer review only

Cardiovascular risk prediction using physical performance measures in COPD: results from a multi-centre observational study.

Jilles M. Fermont[1,2$], Marie Fisk[1], Charlotte E. Bolton[3], William Macnee[4], John Cockcroft[5], Jonathan Fuld[6], Joseph Cheriyan[1], Divya Mohan[7], Kaisa M. Mäki-Petäjä[1], Ali B.A.K. Al-Hadithi[1], Ruth Tal-Singer[7], Hana Müllerova[8], Michael I. Polkey[9], Angela M. Wood[2,10-13], Carmel McEniery[1], Ian B. Wilkinson[1,14], on behalf of the ERICA consortium.

[1]Division of Experimental Medicine and Immunotherapeutics, Department of Medicine, University of Cambridge, Cambridge, United Kingdom;

[2]British Heart Foundation Cardiovascular Epidemiology Unit, Department of Public Health and Primary Care, University of Cambridge, Cambridge, United Kingdom;

[3]Division of Respiratory Medicine and NIHR Nottingham BRC respiratory theme, University of Nottingham, Nottingham, United Kingdom;

[4]Centre for Inflammation Research, Queen's Medical Research Institute, University of Edinburgh, Edinburgh, Scotland United Kingdom;

[5]Department of Cardiology, Columbia University Medical Centre, New York, United States;

[6]Department of Respiratory Medicine, Cambridge University Hospitals NHS Foundation Trust, Cambridge, United Kingdom;

[7]Medical Innovation, Value Evidence Outcomes, GSK R&D, PA, United States;

[8]GSK R&D, Uxbridge, United Kingdom;

[9]Department of Respiratory Medicine, Royal Brompton Hospital, London, United Kingdom;

[10]British Heart Foundation Centre of Research Excellence, University of Cambridge, Cambridge, United Kingdom;

[11]National Institute for Health Research Blood and Transplant Research Unit in Donor Health and Genomics, University of Cambridge, Cambridge, United Kingdom;

[12]National Institute for Health Research Cambridge Biomedical Research Centre, University of Cambridge and Cambridge University Hospitals, Cambridge, United Kingdom;

[13]Health Data Research UK Cambridge, Wellcome Genome Campus and University of Cambridge, Cambridge, United Kingdom;

[14]Cambridge Clinical Trials Unit, Cambridge University Hospitals NHS Foundation Trust, Addenbrooke's Hospital, Cambridge, United Kingdom.

For peer review only

$Corresponding author: Jilles M. Fermont, Division of Experimental Medicine and Immunotherapeutics, Department of Medicine, University of Cambridge, Cambridge CB1 8RN, United Kingdom. Tel: +44 (0)12 2333 6806. E-mail: jmf88@medschl.cam.ac.uk.

Word count: 3398

Keywords: Chronic Obstructive Pulmonary Disease; COPD; Biomarkers; Prognosis; Cardiovascular Diseases.

For peer review only

ABSTRACT

Objectives Although cardiovascular disease (CVD) is a common co-morbidity associated with chronic obstructive pulmonary disease (COPD), it is unknown how to improve prediction of cardiovascular (CV) risk in COPD individuals. Traditional CV risk scores have been tested in different populations but not uniquely in COPD. The potential of alternative markers to improve CV risk prediction in COPD individuals is unknown. We aimed to determine the predictive value of conventional CVD risk factors in COPD, and to determine if additional markers improve prediction beyond conventional factors.

Design Data from the ERICA cohort, which enrolled 729 GOLD stage II-IV COPD individuals were used. Linked hospital episode statistics and survival data were prospectively collected for a median 4.6 years follow-up.

Setting Five UK centres interested in COPD.

Participants Population-based sample including 714 individuals with spirometry-defined COPD, smoked at least ten pack years and who were clinically stable for >4 weeks.

Interventions Baseline measurements included aortic pulse wave velocity (aPWV), carotid intima media thickness (CIMT), C-reactive protein (CRP), fibrinogen, spirometry and BODE-Index, six-minute walk test (6MWT) and four-metre gait speed (4MGS) test.

Primary and secondary outcome measures New occurrence (first event) of fatal or non-fatal hospitalised CVD, and all-cause and cause-specific mortality.

Results Out of 714 participants, 192 (27%) had CV-hospitalisation and six died due to CVD. The overall CV risk model C-statistic was 0.689 (95% CI 0.688 to 0.691). Aortic pulse wave velocity and CIMT had no association with study outcome; nor improved model prediction. CRP, fibrinogen, GOLD stage, BODE Index, 4MGS and 6MWT were associated with the outcome; independently of conventional risk factors ($p < 0.05$ for all). However, only 6MWT improved model discrimination (C = 0.727 [0.726 to 0.728]).

For peer review only

Conclusions Poor physical performance defined by the 6MWT improves prediction of CV hospitalisation in COPD individuals.

Trial registration Clinical Trials Gateway (ID 11101).

For peer review only

ARTICLE SUMMARY

Strengths and limitations of this study

- This is the first study assessing the utility of conventional cardiovascular (CV) disease risk factors for CV risk prediction and the value of additional markers of risk within a COPD cohort.
- Patient level cohort data were linked to hospital admission data (i.e. hospital episode statistics) obtained from the National Health Services in England, Scotland and Wales, and Office for National Statistics record of mortality with analyses limited to five years of follow-up.
- Hospitalised cardiovascular episodes were coded based on ICD-10 classifications extracted from both primary and secondary positions.
- A multivariable prediction model, with 10-fold cross validation and 200 replications, was used to evaluate a wide range of cardiovascular and physical performance biomarkers.
- Generalisability of our results is limited to those with moderate COPD in the UK with NHS hospitalisations.

For peer review only

INTRODUCTION

Chronic obstructive pulmonary disease (COPD) is associated with a two to three-fold increased risk of coronary artery disease and other cardiovascular (CV) comorbidities.[1,2] COPD and cardiovascular disease (CVD) are common conditions that share important common risk factors, such as smoking and physical inactivity, and both tend to affect older people. In addition, both conditions are often associated with raised systemic inflammatory markers and increased arterial stiffness.[3,4]

Given the global healthcare burden associated with CVD,[5] there is incentive to improve the accuracy of CV risk prediction in different populations. Individuals with COPD may be considered constitutively at high CV risk given their age and smoking history. However, it is unknown whether classic CV risk prediction models such as the Framingham general CV risk score, which predicts an individual's ten year risk of developing CVD based on an algorithm of weighted risk factors and has been tested in a number of different populations,[6–9] perform well in individuals with COPD.[10,11]

Conceptually, the performance of the Framingham model might be improved by additional measures. Candidate measurements include surrogate CV risk markers that impart mechanistic information (i.e. aortic pulse wave velocity (aPWV) or carotid intima-media thickness (CIMT)), inflammatory markers (i.e. C-reactive protein (CRP)) or measures of physical performance that enhance CV risk prediction in COPD individuals. In fact, the value of these measures in CV risk prediction have been explored in different population groups. For example, the six-minute walk test (6MWT) significantly improved risk prediction in patients with stable coronary disease.[12] In individuals with intermediate CV risk but without CVD, adding CRP or fibrinogen to conventional risk factors modestly improved CV event prediction.[13] A meta-analysis of aPWV studied in different disease cohorts showed it improved CV event prediction independently of conventional risk factors.[14] In contrast, CIMT measurement, although associated with CV risk factors, did not significantly improve risk prediction beyond traditional factors in individuals with hypertension.[15] Given that individuals with COPD, in addition to having high CV risk based on conventional factors, also have increased aPWV, CIMT[4], and increased inflammatory markers,[16] as well as reduced lung function which is associated with increased CV risk in the general population[17] and reduced physical performance,[18,19] the clinical significance of

For peer review only

these findings in relation to CV risk prediction in individuals with COPD is an important question to address.

The aims of our study were to firstly determine the predictive value of conventional CVD risk factors for CV risk, defined by new occurrence (first event since study enrolment) of fatal or non-fatal hospitalised CVD in individuals with stable global initiative for chronic obstructive lung disease (GOLD) stage II-IV[20] COPD. A secondary aim was to determine whether addition of alternative CV measures (i.e. aPWV and CIMT), inflammatory markers (i.e. CRP and fibrinogen), COPD severity (i.e. GOLD stage and BODE Index), as well as physical performance tests commonly used in COPD research (i.e. 6MWT and four-metre gait speed (4MGS)) improved the predictive value of a CV risk model based on conventional CVD risk factors for CV risk prediction. We addressed these questions in the Evaluation of the Role of Inflammation in Chronic Airways disease (ERICA) COPD cohort using study data and linked UK electronic health records.

For peer review only

METHODS

Study design and participants

The ERICA study is a multi-centre observational, cohort study with 729 individuals with stable GOLD stage II-IV[20] COPD, established to identify important CV and physical performance biomarkers that could be targeted to improve the outcomes of COPD individuals. Participants had a clinical diagnosis of COPD, smoking history of at least ten pack years, post-bronchodilator forced expiratory lung volume in 1 second ($FEV_{1)}$ / forced vital capacity (FVC) ratio <0.7 and FEV_1 ≤80% of predicted normal lung function, and were aged >40 years old and clinically stable for >4 weeks. Full details of the protocol have been provided elsewhere.[21] Baseline data captured included demographics, spirometry, blood circulating biochemical markers, measures of arterial stiffness (i.e. aPWV and augmentation index (AIx)), CIMT and physical performance (i.e. 4MGS and 6MWT). Individuals in the ERICA study were linked with UK National Health Services (NHS) electronic healthcare records (i.e. hospital episode statistics (HES[1]))[22] and Office for National Statistics death data through anonymised IDs provided by the NHS.

Clinical measures

After four hours of fasting, with no bronchodilators for six hours, and ten minutes of supine rest, carotid-femoral aPWV and AIx measurements were taken using a SphygmoCor system as previously described.[23] Carotid intima-media thickness of the common carotid arteries was measured using B-mode ultrasound at a distance of one cm from the carotid bulb with a 7–12 MHz linear probe.[24] The thickest artery of the two was included in the analysis. Fasting blood samples were taken for biochemical analysis including plasma fibrinogen, serum CRP and glucose. Physical performance measures 6MWT[25] and 4MGS[26] were assessed according to guidelines. Diabetes status and anti-hypertensive treatment were self-reported at baseline study visit. Disease severity was defined according to GOLD classification.[20] Points for the BODE (Body mass index (BMI), airflow Obstruction, Dyspnoea, and Exercise capacity) Index were assigned as described by Celli *et al*.[27]

Cardiovascular hospitalisation and mortality

[1] Hospital episode statistics (HES) data is a database that includes details of all hospital admissions, accident and emergency department visits and outpatient appointments at an individual patient level.

For peer review only - http://bmjopen.bmj.com/site/about/guidelines.xhtml

Cardiovascular hospitalisation and mortality data were extracted from the linked hospital admission data and death certificates. Non-fatal CV episodes were extracted from both primary and secondary international statistical classification of diseases and related health problems 10th revision coding (ICD-10) positions. Causes of death were adjudicated by CV and pulmonary physicians. We defined the primary outcome as first reported occurrence (since study enrolment) of fatal or non-fatal hospitalised CVD, where CVD was defined as diseases of the arteries, stroke or heart failure (see supplementary table 1) based on classifications used by the Emerging Risk Factors Collaboration.[28] Time to primary outcome was calculated from the difference between the baseline visit date (starting December 2011) and either the date of death or first hospitalised CV attendance up to November 2017, when follow-up discontinued. Secondary outcomes of interest were all-cause and cause-specific mortality (defined as CV, pulmonary, cancer, or other).

Risk factors of interest

Conventional CVD risk factors included age, sex, self-reported smoking status [current/ex-smoker], high-density lipoprotein (HDL) cholesterol, total cholesterol, systolic blood pressure (SBP), diabetes [yes/no] and treatment for high blood pressure [yes/no]. We also assessed the addition of the following risk factors: BMI, aPWV and CIMT, fasting glucose, CRP and fibrinogen, COPD severity (i.e. GOLD stage and BODE Index), and measures of physical performance (i.e. 6MWT and 4MGS).

Statistical analysis

Hazard ratios with 95% confidence intervals (CI) were estimated using Cox regression models stratified by study centre. Age and sex were added to all models. To quantify the independent association of CIMT we further included systolic blood pressure. For aPWV we also included mean arterial pressure and resting heart rate. For AIx we additionally included resting heart rate and height. Proportional hazards were assessed by Schoenfeld's global tests. We assessed the relationships of the new markers and outcomes and consequently log-transformed the following risk factors: CRP, fibrinogen and glucose. Hazard ratios for log-transformed risk factors represent a twofold increase in the risk factor, whereas others are presented as a change in unit. We evaluated the predictive value of new markers added to the conventional CVD risk factors using measures of discrimination (i.e. Harrell's C-statistic)[29,30] and calibration (i.e. Gronnesby and Borgan test and Brier score).

For peer review only - http://bmjopen.bmj.com/site/about/guidelines.xhtml

Gronnesby and Borgan is an overall calibration test for Cox models based on grouping individuals by their estimated risk score and compares observed and model-based expected events within each group (a P-value > α suggests no difference). Brier scores range from 0-1 (0 is perfect accuracy and 1 perfect inaccuracy), and allows to compare performance of a model with a reference model. The C-statistic is a measure for validating the discriminative ability of a model. Value ranges from 0.5-1.0 (1.0 is perfect prediction and 0.5 a random guess). A higher score indicates better discriminative ability of the model. To optimise efficiency and avoid optimism from internal validation in small samples, we used 10-fold cross validation with 200 replications.[31] See online supplementary text 1 and supplementary figures 1-5 for further statistical analyses details.

Observational data is reported according to the STROBE statement.[32] All tests were two-sided and statistical significance were defined by 95% CI for hazard ratios not traversing 1 or p<0.05. Our analyses were performed using Stata version 13 (College Station. Texas) and R (R Foundation).

For peer review only

RESULTS

Of the 729 individuals included in the study, 714 (98%) could be linked with hospital admission and survival records, and were included in the analysis (online supplementary figure 6). The median age was 67 (interquartile range 62-73) years and 434 (61%) individuals were male (table 1). A third (n = 218) of the cohort smoked, 12% (n = 82) had self-reported diabetes, 34% (n = 245) were taking anti-hypertensive medications, and 31% (n = 224) were taking cholesterol-lowering medications at baseline. The median FEV_1 was 1.3 (0.9-1.7) litres (mean ± SD = 1.34 ± 0.53). In total, 192 individuals (27%) had a first event of CV hospitalisation[2] (n = 116 (60%) were in ICD-10 secondary coding position) during median follow-up for 4.6 years and six individuals had CV death without any preceding CV episode. Cardiovascular hospitalisation accounted for the majority (97%) of events analysed. The CV incidence rate was 6.7 (95% CI 5.8 to 7.7) per 100 person-years. See online supplementary tables 1-2 for categorisation of different admission codes for CV hospitalisation.

Table 1 Baseline characteristics (No. = 714).

Characteristic	Summary measures
**Conventional CVD risk factors**	
Age (yrs.)	67 (62 to 73)
Male	434 (61)
Current smoker	218 (31)
HDL (mmol/L)	1.4 (1.2 to 1.7)
Cholesterol (mmol/L)	5.0 (4.3 to 5.8)
SBP (mmHg)	142 (131 to 154)
Diabetes mellitus	82 (12)
Drugs to treat hypertension	245 (34)
**CV measures**	
aPWV (m/sec)	9.8 (8.4 to 11.8)
CIMT (mm)	0.81 (0.71 to 0.96)

[2] Peripheral arterial disease (n = 9), diseases of arteries, arterioles and capillaries (n = 7), angina (n = 21), unstable angina (n = 3), coronary heart disease not otherwise specified (n = 63), acute myocardial infarction (MI), and certain current complications following acute MI (n = 11), cerebral infarction (n = 11), stroke, not specified as haemorrhage or infarction (n = 3), other stroke (n = 18), heart failure (n = 32), and abdominal aortic aneurysm (n = 8).

For peer review only

Alternative measures	
CRP (mg/L)	1.21 (0.47 to 2.01)
Fibrinogen (g/dL)	1.22 (1.06 to 1.36)
Glucose (mmol/L)	1.59 (1.46 to 4.59)
BMI (kg/m^2)	27 (23 to 31)
GOLD (stage)	2 (2 to 3)
4MGS (m/s)	0.95 (0.77 to 1.14)
6MWT distance (metre)	366 (255 to 440)
BODE (point)	3 (1–5)

Values are given as the median and interquartile range (IQR), or No. of cases (%). CVD = cardiovascular disease. HDL = high-density lipoprotein. SBP = systolic blood pressure. CV = cardiovascular. aPWV = carotid-femoral aortic pulse wave velocity. CIMT = carotid intima-media thickness. CRP = C-reactive protein. BMI = body mass index. GOLD = global initiative for obstructive lung disease. 4MGS = four-metre gait speed. 6MWT = six-minute walk test. BODE = BMI, obstruction (FEV_1), dyspnoea (Medical Research Council), exercise (6MWT).

Conventional cardiovascular disease risk factors

Of the conventional CVD risk factors, age and treatment for high blood pressure had significant positive associations with the study's outcome whereas SBP had a significant negative association and other risk factors (i.e. sex, smoking status, cholesterol, HDL cholesterol and diabetes) were not significantly associated with CV hospitalisation. Use of hypertension drug treatment followed by age and total cholesterol contributed most to the discriminative ability of the model. The overall discriminative ability of the CV risk model had a C-statistic of 0.689, 95% CI 0.688 to 0.691; figure 1 and online supplementary table 3).

PLACE FIGURE 1 HERE

Surrogate cardiovascular risk markers

For peer review only

Except for AIx, neither aPWV nor CIMT were significantly associated with CV hospitalisation, after including conventional CVD risk factors (figure 2 and online supplementary table 4). Moreover, none of the CV risk markers significantly changed the discriminative ability of the CV risk model.

PLACE FIGURE 2 HERE

Physical performance measures, COPD severity and inflammatory markers

Multivariable analysis identified that poor physical performance (i.e. reduced 6MWT distance and slower 4MGS) were significantly associated with increased CV hospitalisation, independently of conventional CVD risk factors. With the exception of glucose and BMI, severity of COPD defined by higher BODE Index and GOLD stage, as well as inflammatory markers (i.e. CRP and fibrinogen) all had significant positive associations with CV hospitalisations independently of conventional CVD risk factors.

Predictive modelling indicated significant improvement in risk discrimination when adding BMI (C = 0.698, 95% CI 0.696 to 0.699), BODE (C = 0.705, 95% CI 0.704 to 0.707), 4MGS (C = 0.712, 95% CI 0.710 to 0.713) or 6MWT (C = 0.727, 95% CI 0.726 to 0.728) to the CV risk model, but not GOLD stage, inflammatory markers or glucose (figure 3 and online supplementary table 5). Calibration tests indicate good model fit (figures 2 and 3). Adding BMI, 4MGS, 6MWT and BODE collectively to the CV risk model resulted in a C-statistic of 0.731 (95% CI 0.729 to 0.732), indicating 6MWT primarily accounted for improved discriminative ability of the model. The model including 6MW had a better Brier score relative to the CV risk model (0.123 vs. 0.129, respectively).

PLACE FIGURE 3 HERE

All-cause and cause-specific mortality

For peer review only

There were 144 deaths in total (nearly 20% of the cohort), over a median follow-up period of 4.6 years. The majority of deaths were in men (n = 96, 67%) and pulmonary disease was the leading cause in both sexes (table 2). Pulmonary disease accounted for 65% of deaths in women and 50% in men, followed by cancer (19% in women, 27% in men), CV (6% and 16%), and other (10% and 7% in women and men respectively).

Table 2 Mortality in the ERICA cohort.

**Cause-specific mortality**	Female No. (%)	Male No. (%)	Total No. (%)
Pulmonary	31 (65)	48 (50)	79 (55)
Cardiovascular	3 (6)	15 (16)	18 (13)
Cancer	9 (19)	26 (27)	35 (24)
Other	5 (10)	7 (7)	12 (8)
**All-cause**	48 (100)	96 (100)	144 (100)

Deaths recorded over median follow up of 4.6 years.

For peer review only

DISCUSSION

This is the first study assessing the utility of conventional CVD risk factors for CV risk prediction and the value of additional markers of risk within a COPD cohort. Novel findings include (i) poor physical performance improved the discriminative power when added to the CV risk model; (ii) BODE Index, GOLD stage, 4MGS and systemic inflammatory markers were positively associated with CV hospitalisations independently of conventional CVD risk factors, although they collectively improved the model's discriminative ability marginally; (iii) of the conventional CVD risk factors; age, SBP and use of anti-hypertensives were positively associated with study outcome. However, age, cholesterol and use of anti-hypertensives contributed most to the model's prognostic power. Systolic blood pressure had a significant negative association with CV hospitalisations but did not add to the model's predictive ability. Furthermore, aPWV and CIMT, despite providing *in-vivo* mechanistic information about the arterial system, had no significant association with CV hospitalisations, whereas AIx did. Therefore, these data suggest that in COPD, physical performance (assessed by 6MWT) contributes to CV risk estimation defined predominantly by non-fatal CV hospitalisations. Finally, CVD contributed to only a small number of deaths at a median follow-up of 4.6 years. Pulmonary disease, followed by cancer were the major causes of death in both men and women with COPD.

We observed a C-statistic of 0.689 based on the conventional CV risk model, which increased to a maximum of 0.731 when adding additional markers; this increment was primarily due to 6MWT. These C-statistic values indicate a moderate predictive ability of the models for the study's outcome. For perspective, a C-statistic of 0.53 was observed in a Framingham model in the very elderly (aged 85-plus; the median age of our cohort was 67 years) without prior CVD, indicating it does not predict CV mortality in this group.[6] However, in primary care individuals without CVD in the Framingham study, a C-statistic of 0.76 for an outcome of general CVD (i.e. coronary heart disease, stroke, peripheral artery disease, or heart failure), demonstrated good discrimination.[10] Since our cohort included individuals with CVD at baseline, and different CVD risk factors and CV outcomes are evaluated in various studies, no direct comparison of study results can be made. That aPWV was not predictive in our cohort contrasts with findings reported by Ben-Shlomo *et al.* However, from the 17 included cohorts in their study, none were COPD cohorts, the mean age of the cohorts was lower, and the proportion of individuals taking drugs to treat hypertension was higher.[14]

For peer review only

Overall, our study emphasises the importance of physical function as a predictor of CV risk. Tests such as 6MWT or 4MGS are proxy measures of overall mobility and physical functioning.[33] Exercise capacity and CV fitness are known to be associated with fatal- and non-fatal CVD,[34] whilst exercise-based cardiac rehabilitation reduces risk of CV events.[35] The 6MWT distance is prognostic in patients with stable coronary heart disease,[12] and those with moderate-to-severe heart failure.[36] In our cohort poor physical function improved discriminative ability of the CV risk model beyond conventional CVD factors. This has implications for clinical practice in CV risk assessment for COPD individuals, suggesting it may be helpful to incorporate physical performance into clinical assessment. Especially those aged under 65 years may benefit most from active CVD assessment, according to Morgan *et al*.[37] However, given that 6MWT can be logistically challenging to set up and time-consuming,[38] the faster and simpler 4MGS that also significantly improved the C-statistic could be used as a simpler alternative. In elderly with CVD, 4MGS is comparable to 6MWT in predicting all-cause mortality.[39]

The BODE Index also significantly improved the C-statistic of the CV risk model. However, this was primarily due to the 6MWT component. In fact, although GOLD stage had a significant positive association with the study outcome, this did not improve prognostic power of the model. Therefore, despite association between airflow limitation and CV risk in general population studies,[17,40] in patients with COPD, physical performance assessment rather than another component of BODE (i.e. spirometry) adds value to CV risk prediction.

Although we did not find an association between inflammatory markers and surrogate markers of CV risk in the baseline cross-sectional component of the ERICA study,[41] both CRP and fibrinogen were associated with CV hospitalisations captured over nearly five years follow-up. That the associations remained significant after including conventional CVD risk factors indicate potential value for identifying high-risk individuals within a COPD population. The inverse relationship of SBP was an unexpected finding. One hypothesis is that SBP has a J-shaped curve for CV events and mortality.[42] Therefore, in this cohort, lower SBP might be a marker of sickness and frailty, hence its association with CV hospitalisations.

For peer review only

A third of the cohort had a CV related hospitalisation during follow-up and the majority had pre-existing CV comorbidity at baseline. The high numbers of CV hospitalisations, gives perspective of what this comorbidity incurs for patients and the huge healthcare costs involved. In contrast to the sizeable number of CV hospitalisations, was the small number of CV deaths. Gayle *et al.*, previously reported that CV-related mortality in chronic lung patients had already started to decline in England.[43] This may reflect better CV risk management reducing CV mortality. Importantly though, such risk management does not seem to impact CV morbidity, and is an area that requires future research to determine the optimum approach to impact CV morbidity in COPD individuals.

Nearly 20% of the cohort died during follow-up. This is comparable to the TORCH study where approximately 15% of the cohort died over 3 years of follow-up.[2] Pulmonary disease, followed by cancer accounted for proportionally more deaths in our cohort compared with TORCH, whereas CV-specific mortality was less (10-15% vs 27% in TORCH). Reasons for these findings are not entirely understood but the sizeable proportion of our cohort already on medicines for dyslipidaemia and blood pressure control may be an important factor.

Our study has limitations. The conventional CVD risk factors are usually used to predict CV risk defined by development of CVD. However, the majority of our participants already had CVD and were on CV medications, which may be a confounding factor impacting the discriminative ability of CVD risk factors. Moreover, in our study, CV risk was defined differently (as CV hospitalisation with CV mortality). Hospitalised CV episodes were coded based on ICD-10 classifications[28] extracted from both primary and secondary positions. Notably, most CV hospitalisations were recorded in secondary positions, indicating that the primary admission might be related to something else. Due to the few CV events recorded in the primary position, we were unable to conduct a sensitivity analysis including CV events in the primary position only. The study period covered the time from study enrolment until the end of study, or death. Some individuals, however, may have been admitted to hospital for CV events before study enrolment. We were unable to obtain HES data outside this period. Competing risks may have occurred, for example, individuals who died of other causes may not have experienced a CV event during the study period for this reason. However, our study did not have sufficient statistical

For peer review only - http://bmjopen.bmj.com/site/about/guidelines.xhtml

power to assess this. It is possible our models predict non-CV hospitalisation due to, for example, potential cross-contamination of COPD and CV hospitalisation as a result of misclassification of morbidity and mortality that often occurs when data are obtained from routine sources.[44] Deprivation scores may be another important determinant of CV risk in COPD individuals that is worth evaluation in future research,[45] although the relatively small cohort size of our study meant we did not include this in the analysis.

Furthermore, follow-up time for the study was a maximum of five years, whereas risk scores such as Framingham Risk Score and QRISK calculate a 10-year risk. Hence, the prognostic value of variables may alter with a different time horizon and depends on the extent of time trends in the new biomarkers. For example, a too short time period may result in an insufficient number of events, whilst over a longer time period, e.g. 20 years, the predictive ability would diminish because ageing is a strong predictor. We did not have access to an independent validation cohort but used cross-validation techniques instead. Generalisability of our results is limited to those with moderate COPD in the UK with NHS hospitalisations.

CONCLUSION

In a UK COPD cohort, poor physical performance assessed by 6MWT or 4MGS and inflammatory biomarkers are associated with subsequent CV-related hospitalisations, independently of conventional CVD risk factors. Importantly, poor physical performance (defined primarily by 6MWT), also significantly improved the predictive discrimination of the CV risk model. These data suggest an assessment of physical performance may enhance CV risk evaluation in individuals with COPD.

ACKNOWLEDGMENTS

The views and opinions expressed are those of the authors and do not necessarily reflect those of the University of Cambridge, the NHS, the NIHR, the Department of Health and Social Care, or other parent institutions of the authors. The corresponding author and IBW had full access to all the data in the study and takes responsibility for the integrity of the data and the accuracy of the data analysis.

For peer review only

Contributors: The following authors made substantial contributions to the conceptualisation or design: JMF, RTS, HM, MIP, AMW and IBW; data curation: JMF, IBW, KMP and AAH; formal analysis: JMF; interpretation: JMF, MF and IBW; funding acquisition: RTS, MIP and IBW; investigation: JMF, MF, CEB, WM, JRC, JF, JC, DM, KMP, AAH, MIP, CMM and IBW; methodology: JMF, AMW, IBW; project administration: JMF, RTS, MIP and IBW; resources: JMF, MF, CEB, WM, JRC, DM, MIP, CMM and IBW; software: JMF; supervision: DM, RTS, HM, MIP, AMW and IBW; validation: JMF; visualisation: JMF; and writing – original draft preparation: JMF. All authors contributed to the reviewing and editing of the manuscript.

Competing interests: All authors have completed the ICMJE uniform disclosure form at www.icmje.org/coi_disclosure.pdf and declare: GSK, a consortium partner, funded JMFs PhD. HM, RTS and DM were employees of GSK at the time this work was completed and own GSK shares and stock options. JC is employed by Cambridge University Hospitals NHS Foundation Trust and is obligated to spend 50% of his time on GSK clinical trial activity, representing a significant relationship; however, he receives no other benefits or compensation from GSK. MIP received grants from GSK outside the submitted work. IBW received grants from GSK during the conduct of the study and outside the submitted work.

Funding: This work was supported by a grant (9157-61188) from Innovate UK (formerly known as Technology Strategy Board) with contributory funding in kind (e.g. scientific expertise and meeting rooms) from GSK, a consortium partner, who also funded the corresponding author's PhD. As a consortium partner, GSK was involved in the study design, data analysis, decision to publish, and preparation of the manuscript. The specific roles of all authors are articulated in the 'author contributions' section. RTS was a co-investigator on the grant and as a consortium member was involved in the decision to publish, and preparation of the manuscript. IBW, JC and CMM acknowledge funding support from the NIHR Cambridge Comprehensive Biomedical Research Centre. CEB is supported by the NIHR Nottingham BRC respiratory theme. This work was supported by core funding from: the UK Medical Research Council (MR/L003120/1), the British Heart Foundation (RG/13/13/30194; RG/18/13/33946) and the National Institute for Health Research [Cambridge Biomedical Research Centre at the Cambridge University Hospitals NHS Foundation Trust] [*]. This

For peer review only - http://bmjopen.bmj.com/site/about/guidelines.xhtml

work was also supported by Health Data Research UK, which is funded by the UK Medical Research Council, Engineering and Physical Sciences Research Council, Economic and Social Research Council, Department of Health and Social Care (England), Chief Scientist Office of the Scottish Government Health and Social Care Directorates, Health and Social Care Research and Development Division (Welsh Government), Public Health Agency (Northern Ireland), British Heart Foundation and Wellcome. *The views expressed are those of the authors and not necessarily those of the NHS, the NIHR or the Department of Health and Social Care.

Data availability: The dataset underlying these findings, with de-identified participant data (including the data dictionary), are available to interested and qualified researchers upon request and can be obtained from the Cambridge Clinical Trials Unit. Access to hospital episode statistics requires a data sharing agreement with the National Health Services. For data access, please contact cctu@addenbrookes.nhs.uk.

Patient consent for publication: Informed patient consent was obtained in writing from all study participants and permits the publishing of all data included in this article. The investigation conforms with the principles outlined in the Declaration of Helsinki. Ethical approval was granted by the National Research Ethics Service Committee East of England - Cambridge South (11/EE/0357).

Patient and public involvement: Patients and public were not involved in the design, conduct or reporting of this study.

For peer review only

REFERENCES

1 Chen W, Thomas J, Sadatsafavi M, *et al.* Risk of cardiovascular comorbidity in patients with chronic obstructive pulmonary disease: a systematic review and meta-analysis. *Lancet Respir Med* 2015;**3**:631–9. doi:10.1016/S2213-2600(15)00241-6

2 McGarvey LP, John M, Anderson JA, *et al.* Ascertainment of cause-specific mortality in COPD: operations of the TORCH Clinical Endpoint Committee. *Thorax* 2007;**62**:411–5.

3 Garcia-Rio F, Miravitlles M, Soriano JB, *et al.* Systemic inflammation in chronic obstructive pulmonary disease: a population-based study. *Respir Res* 2010;**11**:63. doi:10.1186/1465-9921-11-63

4 Fisk M, McEniery CM, Gale N, *et al.* Surrogate Markers of Cardiovascular Risk and Chronic Obstructive Pulmonary Disease: A Large Case-Controlled Study. *Hypertension* 2018;**71**:499–506. doi:10.1161/HYPERTENSIONAHA.117.10151

5 Lozano R, Naghavi M, Foreman K, *et al.* Global and regional mortality from 235 causes of death for 20 age groups in 1990 and 2010: a systematic analysis for the Global Burden of Disease Study 2010. *Lancet* 2012;**380**:2095–128. doi:10.1016/S0140-6736(12)61728-0

6 de Ruijter W, Westendorp RG, Assendelft WJ, *et al.* Use of Framingham risk score and new biomarkers to predict cardiovascular mortality in older people: population based observational cohort study. *BMJ* 2009;**338**:a3083. doi:10.1136/bmj.a3083

7 Wannamethee SG, Shaper AG, Lennon L, *et al.* Metabolic syndrome vs Framingham Risk Score for prediction of coronary heart disease, stroke, and type 2 diabetes mellitus. *Arch Intern Med* 2005;**165**:2644–50. doi:10.1001/archinte.165.22.2644

8 Weiner DE, Tighiouart H, Elsayed EF, *et al.* The framingham predictive instrument in chronic kidney disease. *J Am Coll Cardiol* 2007;**50**:217–24. doi:10.1016/j.jacc.2007.03.037

9 Samia M, F. RR, Richey SA, *et al.* Enhanced Risk Assessment in Asymptomatic Individuals With Exercise Testing and Framingham Risk Scores. *Circulation* 2005;**112**:1566–72. doi:10.1161/CIRCULATIONAHA.105.542993

10 D'Agostino RB, Vasan RS, Pencina MJ, *et al.* General cardiovascular risk profile for use in primary care. *Circulation* 2008;**117**:743–53.

11 Lee HM, Lee J, Lee K, *et al.* Relation Between COPD Severity and Global Cardiovascular Risk in US Adults. *Chest* 2012;**142**:1118–25. doi:10.1378/chest.11-2421

For peer review only

12 Beatty AL, Schiller NB, Whooley MA. Six-minute walk test as a prognostic tool in stable coronary heart disease: data from the heart and soul study. *Arch Intern Med* 2012;**172**:1096–102. doi:10.1001/archinternmed.2012.2198

13 Emerging Risk Factors Collaboration, Kaptoge S, Di Angelantonio E, *et al.* C-reactive protein, fibrinogen, and cardiovascular disease prediction. *N Engl J Med* 2012;**367**:1310–20. doi:10.1056/NEJMoa1107477

14 Ben-Shlomo Y, Spears M, Boustred C, *et al.* Aortic pulse wave velocity improves cardiovascular event prediction: an individual participant meta-analysis of prospective observational data from 17,635 subjects. *J Am Coll Cardiol* 2014;**63**:636–46. doi:10.1016/j.jacc.2013.09.063

15 Bots ML, Groenewegen KA, Anderson TJ, *et al.* Common carotid intima-media thickness measurements do not improve cardiovascular risk prediction in individuals with elevated blood pressure: the USE-IMT collaboration. *Hypertens (Dallas, Tex 1979)* 2014;**63**:1173–81. doi:10.1161/HYPERTENSIONAHA.113.02683

16 Gan WQ, Man SFP, Senthilselvan A, *et al.* Association between chronic obstructive pulmonary disease and systemic inflammation: a systematic review and a meta-analysis. *Thorax* 2004;**59**:574–80. doi:10.1136/thx.2003.019588

17 Hole DJ, Watt GC, Davey-Smith G, *et al.* Impaired lung function and mortality risk in men and women: findings from the Renfrew and Paisley prospective population study. *BMJ* 1996;**313**:711–6. doi:10.1136/bmj.313.7059.711

18 Sciurba F, Criner GJ, Lee SM, *et al.* Six-Minute Walk Distance in Chronic Obstructive Pulmonary Disease. *Am J Respir Crit Care Med* 2003;**167**:1522–7. doi:10.1164/rccm.200203-166OC

19 Camarri B, Eastwood PR, Cecins NM, *et al.* Six minute walk distance in healthy subjects aged 55–75 years. *Respir Med* 2006;**100**:658–65. doi:10.1016/j.rmed.2005.08.003

20 Global Initiative for Chronic Obstructive Lung Disease (GOLD). Global Strategy for the Diagnosis, Management and Prevention of COPD. 2016. http://goldcopd.org/global-strategy-diagnosis-management-prevention-copd-2016/

21 Mohan D, Gale NS, McEniery CM, *et al.* Evaluating the role of inflammation in chronic airways disease: the ERICA study. *COPD* 2014;**11**:552–9. doi:10.3109/15412555.2014.898031

For peer review only

22 Herbert A, Wijlaars L, Zylbersztejn A, *et al.* Data Resource Profile: Hospital Episode Statistics Admitted Patient Care (HES APC). *Int J Epidemiol* 2017;**46**:1093-1093i. doi:10.1093/ije/dyx015

23 Wilkinson IB, Fuchs SA, Jansen IM, *et al.* Reproducibility of pulse wave velocity and augmentation index measured by pulse wave analysis. *J Hypertens* 1998;**16**:2079–84.https://www.ncbi.nlm.nih.gov/pubmed/9886900

24 Csanyi A, Egervari A. Simple clinical method of average intima-media thickness measurement in the common carotid artery. *Vasa* 1996;**25**:242–8.http://www.ncbi.nlm.nih.gov/pubmed/8795305

25 Laboratories ATSC on PS for CPF. ATS statement: guidelines for the six-minute walk test. *Am J Respir Crit Care Med* 2002;**166**:111–7. doi:10.1164/ajrccm.166.1.at1102

26 Guralnik JM, Simonsick EM, Ferrucci L, *et al.* A short physical performance battery assessing lower extremity function: association with self-reported disability and prediction of mortality and nursing home admission. *J Gerontol* 1994;**49**:M85-94.http://www.ncbi.nlm.nih.gov/pubmed/8126356

27 Celli BR, Cote CG, Marin JM, *et al.* The body-mass index, airflow obstruction, dyspnea, and exercise capacity index in chronic obstructive pulmonary disease. *N Engl J Med* 2004;**350**:1005–12. doi:10.1056/NEJMoa021322

28 Emerging Risk Factors Collaboration. The Emerging Risk Factors Collaboration: analysis of individual data on lipid, inflammatory and other markers in over 1.1 million participants in 104 prospective studies of cardiovascular diseases. *Eur J Epidemiol* 2007;**22**:839–69. doi:10.1007/s10654-007-9165-7

29 Altman DG, Vergouwe Y, Royston P, *et al.* Prognosis and prognostic research: validating a prognostic model. *BMJ* 2009;**338**:b605.

30 Newson RB. Comparing the predictive powers of survival models using Harrell's C or Somers' D. *Stata J* 2010;**10**:339–58.http://www.stata-journal.com/article.html?article=st0198

31 Smith GC, Seaman SR, Wood AM, *et al.* Correcting for optimistic prediction in small data sets. *Am J Epidemiol* 2014;**180**:318–24. doi:10.1093/aje/kwu140

32 von Elm E, Altman DG, Egger M, *et al.* The Strengthening the Reporting of Observational Studies in Epidemiology (STROBE) statement: guidelines for reporting observational studies. *J Clin Epidemiol* 2008;**61**:344–9. doi:10.1016/j.jclinepi.2007.11.008

For peer review only

33 Lord SR, Menz HB. Physiologic, psychologic, health predictors of 6-minute walk performance in older people. *Arch Phys Med Rehabil* 2002;**83**:907–11. doi:10.1053/apmr.2002.33227

34 Newman AB, Simonsick EM, Naydeck BL, *et al.* Association of long-distance corridor walk performance with mortality, cardiovascular disease, mobility limitation, and disability. *Jama-Journal Am Med Assoc* 2006;**295**:2018–26. doi:DOI 10.1001/jama.295.17.2018

35 Anderson L, Oldridge N, Thompson DR, *et al.* Exercise-Based Cardiac Rehabilitation for Coronary Heart Disease Cochrane Systematic Review and Meta-Analysis. *J Am Coll Cardiol* 2016;**67**:1–12. doi:10.1016/j.jacc.2015.10.044

36 Opasich C. Six-minute walking performance in patients with moderate-to-severe heart failure; is it a useful indicator in clinical practice? *Eur Heart J* 2001;**22**:488–96. doi:10.1053/euhj.2000.2310

37 Morgan AD, Zakeri R, Quint JK. Defining the relationship between COPD and CVD: what are the implications for clinical practice? *Ther Adv Respir Dis* 2018;**12**:1753465817750524. doi:10.1177/1753465817750524

38 Holland AE, Spruit MA, Troosters T, *et al.* An official European Respiratory Society/American Thoracic Society technical standard: field walking tests in chronic respiratory disease. *Eur Respir J* 2014;**44**:1428–46. doi:10.1183/09031936.00150314

39 Kamiya K, Hamazaki N, Matsue Y, *et al.* Gait speed has comparable prognostic capability to six-minute walk distance in older patients with cardiovascular disease. *Eur J Prev Cardiol* 2017;**25**:212–9. doi:10.1177/2047487317735715

40 D. SD, Paul MSF. Why Are Patients With Chronic Obstructive Pulmonary Disease at Increased Risk of Cardiovascular Diseases? *Circulation* 2003;**107**:1514–9. doi:10.1161/01.CIR.0000056767.69054.B3

41 Duvoix A, Dickens J, Haq I, *et al.* Blood fibrinogen as a biomarker of chronic obstructive pulmonary disease. *Thorax* 2013;**68**:670–6. doi:10.1136/thoraxjnl-2012-201871

42 SUMMIT Investigators, Brook RD, Byrd JB, *et al.* Blood pressure, heart rate, and mortality in chronic obstructive pulmonary disease: the SUMMIT trial. *Eur Heart J* 2018;**39**:3128–34. doi:10.1093/eurheartj/ehy451

43 Gayle A V, Axson EL, Bloom CI, *et al.* Changing causes of death for patients with chronic respiratory disease in England, 2005-2015. *Thorax* 2019;**74**:483 LP – 491.

For peer review only

doi:10.1136/thoraxjnl-2018-212514

44 Macnee W, Maclay J, McAllister D. Cardiovascular injury and repair in chronic obstructive pulmonary disease. *Proc Am Thorac Soc* 2008;**5**:824–33. doi:10.1513/pats.200807-071TH

45 Pujades-Rodriguez M, Timmis A, Stogiannis D, *et al.* Socioeconomic deprivation and the incidence of 12 cardiovascular diseases in 1.9 million women and men: implications for risk prediction and prevention. *PLoS One* 2014;**9**:e104671–e104671. doi:10.1371/journal.pone.0104671

For peer review only

Figure legends

Figure 1. Conventional cardiovascular disease risk factors at baseline, their hazard ratios and discriminative ability for fatal or non-fatal hospitalised cardiovascular disease.

Values are given as the median and interquartile range (IQR), or No. of cases (%). Baseline data of 714 patients are included. All models are stratified by recruitment site.

[a]Model includes age and sex.

[b]Model includes conventional cardiovascular disease risk factors: age, sex, smoking, high-density lipoprotein, total cholesterol, systolic blood pressure, diabetes, hypertension medication.

CI = confidence interval. HDL = high-density lipoprotein. SBP = systolic blood pressure. CV = cardiovascular.

There were < 5% missing values for descriptive variables such as BMI and smoking status. Missing values were addressed using multiple imputations using chained equations.

Figure 2. Aortic stiffness at baseline, their hazard ratios and discriminative ability for fatal or non-fatal hospitalised cardiovascular disease.

Values are given as the median and interquartile range (IQR), or No. of cases (%). Baseline data of 714 patients are included. All models are stratified by recruitment site.

Gronnesby and Borgan goodness of fit (chi2(3), P > chi2): CV risk model (2.07, 0.559), aPWV (1.64, 0.652), CIMT (2.32, 0.509), AIx (3.08, 0.380). Estimates based on quartiles of risk.

Brier score (95% CI): CV risk model 0.129 (0.111 to 0.146), aPWV 0.126 (0.108 to 0.145), CIMT 0.128 (0.110 to 0.147), AIx 0.126 (0.109 to 0.144). Lower score indicates better accuracy of estimates.

[a]Model includes age and sex.

[b]Model includes conventional cardiovascular disease risk factors: age, sex, smoking, high-density lipoprotein, total cholesterol, systolic blood pressure, diabetes, hypertension medication. Carotid intima-media thickness further included systolic blood pressure. Carotid-femoral aortic pulse wave velocity further included mean arterial pressure and resting heart rate. AIx further included resting heart rate and height.

CI = confidence interval. PWV = pulse wave velocity. CIMT = carotid intima-media thickness. AIx = augmentation index.

For peer review only

For peer review only - http://bmjopen.bmj.com/site/about/guidelines.xhtml

There were about 10% missing values for variables CIMT (n = 66) and aPWV (n = 60). Missing values were addressed using multiple imputations using chained equations.

Figure 3. Alternative measures at baseline, their hazard ratios and discriminative ability for fatal or non-fatal hospitalised cardiovascular disease.

Values are given as the median and interquartile range (IQR), or No. of cases (%). Baseline data of 714 patients are included. All models are stratified by recruitment site.

Gronnesby and Borgan goodness of fit (chi2(3), P > chi2): CV risk model (2.07, 0.559), CRP (0.32, 0.956), Fibrinogen (1.48, 0.687), Glucose (0.42, 0.936), BMI (1.56, 0.668), GOLD (5.63, 0.131), 4MGS (4.70, 0.195), 6MW (2.94, 0.401), BODE (6.46, 0.091), BMI, 4MGS, 6MW, BODE (4.12, 0.249). Estimates based on quartiles of risk.

Brier score (95% CI): CV risk model 0.129 (0.111 to 0.146), CRP 0.125 (0.107 to 0.1423), Fibrinogen 0.128 (0.111 to 0.146), Glucose 0.128 (0.111 to 0.146), BMI 0.128 (0.111 to 0.146), GOLD 0.128 (0.110 to 0.146), 4MGS 0.127 (0.110 to 0.144), 6MW 0.123 (0.105 to 0.140), BODE 0.124 (0.106 to 0.142), BMI, 4MGS, 6MW, BODE 0.122 (0.104 to 0.140). Lower score indicates better accuracy of estimates.

[a]Model includes age and sex.

[b]Model includes conventional cardiovascular disease risk factors: age, sex, smoking, high-density lipoprotein, total cholesterol, systolic blood pressure, diabetes, hypertension medication.

CI = confidence interval. CRP = C-reactive protein. BMI = body mass index. GOLD = global initiative for chronic obstructive lung disease. 4MGS = four-metre gait speed. 6MWT = six-minute walk test. BODE = body mass index, obstruction, dyspnoea, exercise.

There were < 5% missing values for biochemical markers including fibrinogen and cholesterol. Missing values were addressed using multiple imputations using chained equations.

For peer review only

Online supplementary material

Supplementary text 1 Supplementary methods.

Supplementary table 1 Definitions of diagnoses by ICD-10 coding.

Supplementary table 2 Fatal or non-fatal hospitalised cardiovascular disease by ICD-10 coding position.

Supplementary table 3 Conventional cardiovascular disease risk factors at baseline, their hazard ratios for cardiovascular disease.

Supplementary table 4 Arterial stiffness at baseline, their hazard ratios for cardiovascular disease.

Supplementary table 5 Alternative measures at baseline, their hazard ratios for cardiovascular disease.

Supplementary figure 1 Percentage of missing values.

Supplementary figure 2 Percentage and pattern of missing values in key baseline characteristics.

Supplementary figure 3 Differences in baseline pulse wave velocity.

Supplementary figure 4 Differences in baseline carotid intima-media thickness.

Supplementary figure 5 Differences in baseline augmentation index.

Supplementary figure 6 Participant enrolment flow diagram with five years follow up.

For peer review only

Risk factor	Median (IQR) or n (%)	HR (95% CI)[a]	HR (95% CI)[b]	C−statistic
All conventional CVD risk factors				0.689 [0.688 to 0.691]
Age – per 10 year increase	67 (62–73)	1.58 (1.30 to 1.92)	1.59 (1.29 to 1.99)	0.619 [0.618 to 0.621]
Sex – male	434 (61)	1.28 (0.94 to 1.74)	1.16 (0.84 to 1.60)	0.585 [0.581 to 0.588]
Smoking – current	218 (31)	0.95 (0.68 to 1.31)	0.92 (0.66 to 1.29)	0.515 [0.512 to 0.517]
HDL – per 1 mmol/L increase	1.4 (1.2–1.7)	0.73 (0.52 to 1.03)	0.86 (0.60 to 1.23)	0.571 [0.569 to 0.573]
Cholesterol – per 1 mmol/L increase	5.0 (4.3–5.8)	0.80 (0.70 to 0.92)	0.89 (0.76 to 1.03)	0.613 [0.611 to 0.614]
SBP – per 10 mmHg increase	142 (131–154)	0.91 (0.84 to 0.99)	0.90 (0.82 to 0.98)	0.532 [0.530 to 0.535]
Diabetes – yes	82 (12)	1.50 (1.01 to 2.22)	1.15 (0.76 to 1.75)	0.538 [0.534 to 0.539]
Hypertension medication – yes	245 (34)	1.79 (1.34 to 2.38)	1.76 (1.31 to 2.36)	0.620 [0.616 to 0.623]

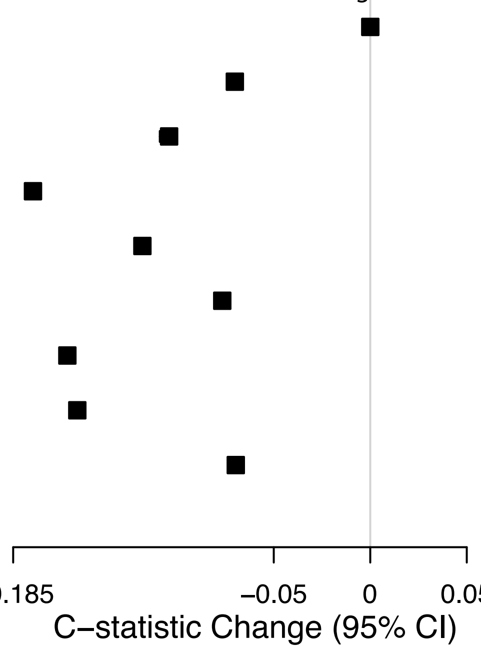

For peer review only

Risk factor	Median (IQR) or n (%)	HR (95% CI)[a]	HR (95% CI)[b]	C−statistic
All conventional CVD risk factors				0.689 [0.688 to 0.691]
+ PWV – per 1 m/sec increase	9.8 (8.4–11.8)	1.01 (0.95 to 1.07)	0.99 (0.93 to 1.06)	0.691 [0.689 to 0.692]
+ CIMT – per 1 mm increase	0.81 (0.71–0.96)	1.09 (0.49 to 2.45)	1.08 (0.48 to 2.43)	0.691 [0.690 to 0.693]
+ AIx – per 5% increase	28 (20–34)	0.85 (0.77 to 0.93)	0.90 (0.82 to 0.99)	0.688 [0.686 to 0.689]

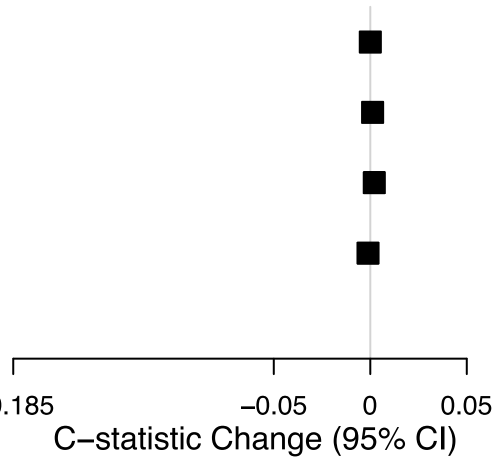

For peer review only
For peer review only - http://bmjopen.bmj.com/site/about/guidelines.xhtml

Risk factor	Median (IQR) or n (%)	HR (95% CI)[a]	HR (95% CI)[b]	C–statistic
All conventional CVD risk factors				0.689 [0.688 to 0.691]
+ CRP – per twofold increase	1.21 (0.47 to 2.01)	1.22 (1.07 to 1.39)	1.20 (1.06 to 1.37)	0.693 [0.691 to 0.694]
+ Fibrinogen – per twofold increase	1.22 (1.06 to 1.36)	2.27 (1.20 to 4.31)	2.12 (1.10 to 4.07)	0.693 [0.691 to 0.694]
+ Glucose – per twofold increase	1.59 (1.46 to 4.59)	1.18 (0.46 to 2.99)	0.83 (0.32 to 2.18)	0.690 [0.688 to 0.691]
+ BMI – per 1 kg/m^2 increase	27 (23–31)	1.04 (1.01 to 1.06)	1.02 (0.99 to 1.05)	0.698 [0.696 to 0.699]
+ GOLD – per 1 stage increase	1 (1–2)	1.22 (0.98 to 1.52)	1.29 (1.03 to 1.61)	0.689 [0.687 to 0.691]
+ 4MGS – per 1 second increase	4.2 (3.5 to 5.2)	1.09 (1.03 to 1.16)	1.07 (1.01 to 1.14)	0.712 [0.710 to 0.713]
+ 6MW distance – per 30 meter increase	366 (255 to 440)	0.91 (0.88 to 0.94)	0.91 (0.88 to 0.95)	0.727 [0.726 to 0.728]
+ BODE – per 1 point increase	3 (1–5)	1.13 (1.07 to 1.20)	1.15 (1.08 to 1.21)	0.705 [0.704 to 0.707]
+ BMI, 4MGS, 6MW, BODE – per 1 unit increase				0.731 [0.729 to 0.732]

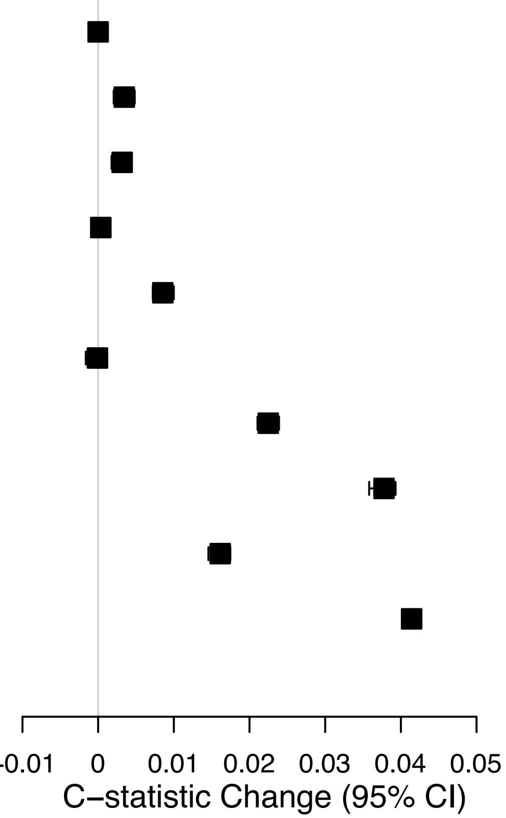

For peer review only

For peer review only - http://bmjopen.bmj.com/site/about/guidelines.xhtml

Supplementary materials for "Cardiovascular risk prediction using physical performance measures in COPD: results from a multi-centre observational study."

Jilles M. Fermont, Marie Fisk, Charlotte E. Bolton, William Macnee, John Cockcroft, Jonathan Fuld, Joseph Cheriyan, Divya Mohan, Kaisa M. Mäki-Petäjä, Ali B.A.K. Al-Hadithi, Ruth Tal-Singer, Hana Müllerova, Michael I. Polkey, Angela M. Wood, Carmel McEniery, Ian B. Wilkinson, on behalf of the ERICA consortium.

For peer review only

Supplementary text

Text 1 Supplementary methods

There were missing values. Data were assessed for the level and type of missing data, and completion patterns (Figures 1-2). There were about 10% missing values for variables CIMT (n = 66) and PWV (n = 60), with <5% missing values for other variables (Figures 3-5). Missing values were addressed using multiple imputations using chained equations (MICE). The time-to-event outcome was included using the non-parametric Nelson-Aalen estimator. Predictive mean matching was used for continuous variables, ordered logistic regression (as continuous) for ordinal variables, multinomial logistic regression for categorical variables, and logistic regression for binary variables. Derived variables such as the BODE Index (a composite score of BMI, forced expiratory volume in one second (FEV_1), Medical Research Council dyspnoea score, and 6MWT distance) and GOLD stage were estimated post MICE using passive imputation.

Data were cleaned for episode status, and inpatient (i.e. hospitalised) CV episodes were identified based on classifications used by the Emerging Risk Factors Collaboration.[1] Cardiovascular events were extracted from both primary and secondary positions of ICD-10. Priority was given to CV events reported in the primary position (n = 70). Following this, we selected CV events in the secondary position (n = 116), which had no recording of a CV event in the primary position. Only episodes during the study follow-up were evaluated.

[1] The Emerging Risk Factors Collaboration: analysis of individual data on lipid, inflammatory and other markers in over 1.1 million participants in 104 prospective studies of cardiovascular diseases. *Eur J Epidemiol* **22,** 839–869 (2007). https://doi.org/10.1007/s10654-007-9165-7

For peer review only

Supplementary tables

Table 1 Definitions of diagnoses by ICD-10 coding.

End point	ICD-10 codes
**All cardiovascular disease**	**F01, G46.3-G46.7, G458, G459 I11.0, I13.0, I13.2, I20.0-I20.1, I20.8-I25, I50, I60, I61, I.62, I63, I64, I65-I69, I70.2, I71.3-I71.9, I72, I73.9-I79 + cardiac death**
**Diseases of the arteries**	**I70.2, I72, I73.9-I79**
Peripheral arterial disease	I70.2, I73.9
Diseases of arteries, arterioles and capillaries	I72, I74-I79
**Coronary heart disease**	**I20.0-I20.1, I20.8-I25**
Angina	I20.1, I20.8-I20.9
Unstable angina	I20.0, I24
Coronary heart disease not otherwise specified	I25
Acute myocardial infarction (MI), and certain current complications following acute MI	I21, I23
Subsequent myocardial infarction	I22
**All stroke**	**I60, I61, I.62, I63, I64, I65-I69, F01, G46.3-G46.7, G458, G459**
Subarachnoid haemorrhage	I60
Intra-cerebral haemorrhage	I61
Cerebral infarction	I63
Stroke, not specified as haemorrhage or infarction	I64
Stroke syndromes	G46.3-G46.7
Transient ischemic attack	G458, G459
Other stroke	I62, I65-I69, F01
**Heart failure**	**I11.0, I13.0, I13.2, I50**
Heart failure	I50
Hypertensive heart disease with (congestive) heart failure	I11.0
Hypertensive heart and renal disease with (congestive) heart failure	I13.0
Hypertensive heart and renal disease with both (congestive) heart failure and renal disease	I13.2
**Cardiac death**	**Adjudicated**
**Other vascular deaths**	**I71.3-I71.9**
Abdominal aortic aneurysm	I71.3-I71.9

For peer review only

Table 2 Fatal or non-fatal hospitalised cardiovascular disease by ICD-10 coding position.

	All cardiovascular disease			AECOPD	
	**Primary ICD-10 position**	**Secondary ICD-10 position**	**Cardiac death**	**Primary ICD-10 position**	**Secondary ICD-10 position**
1	I714			J441	
2	I209			J441	
3	I501				
4	I771			J440	
5	I251				
6	I251				
7	I219				J440
8	I251				
9	I745				
10	I259				
11	I251			J441	
12	I251				
13	I500			J440	
14	I743			J441	
15	I64X			J440	
16	I714				
17	I219			J440	
18	I211				
19	I64X				
20	I739				
21	I739				
22	I639				
23	I251			J441	
24	I652				
25	I251			J440	
26	I251				
27	I632				
28	I251			J440	
29	I660			J440	
30	I500			J441	
31	I771				
32	I251			J441	
33	I638			J441	
34	I702			J440	
35	I200			J441	
36	I652				
37	I501			J441	
38	I214				
39	I64X			J440	
40	I509				
41	I739				
42	I739				
43	I639				J440
44	I743				
45	I251				
46	I251			J440	
47	I219				
48	I638				
49	I251			J440	
50	I251			J440	
51	I639				J440

For peer review only

52	I500				
53	I251				
54	I251			J441	
55	I639				
56	I652				J440
57	I219				
58	I635				
59	I214			J440	
60	I251				
61	I739				J440
62	I639				
63	I251			J440	
64	I200				J440
65	I214				J440
66	I251				
67	I634				J440
68	I209				J440
69	I714				
70	I509				
71		I209		J440	
72		I501			
73		I713			J440
74		I714			
75		I252			J440
76		I259		J440	
77		I252		J440	
78		I500		J441	
79		I694			
80		I209			
81		I209			
82		I259		J441	
83		I509		J440	
84		I500			J440
85		I509			J440
86		I200			
87		I219		J440	
88		I714			J440
89		I780			J440
90		I219		J440	
91		I252		J440	
92		I714		J441	
93		I209			J440
94		I251		J440	
95		I259			J440
96		I501		J440	
97		I252			
98		I252			
99		I500			J440
100		I252			J440
101		I252			
102		I652			
103		I500		J440	
104		I259		J441	
105		I251			
106		I501		J440	
107		I252			
108		I255			J440
109		I259			J440

110		I500		J440	
111		I252			
112		I252		J440	
113		I259			
114		I678		J441	
115		I501			J440
116		I678		J440	
117		I501			J440
118		I678		J440	
119		I259			
120		I209			
121		I209		J441	
122		I500		J441	
123		I259			J440
124		I259			J440
125		I259			J440
126		I209		J440	
127		I252			J440
128		I509		J440	
129		I209			
130		I259			
131		I259			
132		I259		J440	
133		I209			
134		I679			
135		I678		J440	
136		I500		J440	
137		I501			
138		I500		J441	
139		I209			
140		I652		J440	
141		I209			
142		I678			
143		I209			
144		I209		J441	
145		I501		J441	
146		I209		J440	
147		I209		J440	
148		I739			
149		I509			
150		I678		J441	
151		I252		J440	
152		I252			
153		I501		J440	
154		I678			
155		I251		J440	
156		I252			J440
157		I500		J440	
158		I252		J440	
159		I252		J440	
160		I634			
161		I679			
162		I252		J440	
163		I252			
164		I500			J440
165		I209		J441	
166		I739			J440
167		I739		J440	

For peer review only

168		I259			
169		I509			J440
170		I252			
171		I209			J440
172		I798		J440	
173		I500			
174		I209			
175		I501		J440	
176		I219		J441	
177		I209			
178		I252		J440	
179		I252			
180		I671		J441	
181		I259			
182		I259		J440	
183		I671			
184		I259			J440
185		I251			J440
186		I714			J440
187			Cardiac		
188			Cardiac		
189			Cardiac		
190			Cardiac		
191			Cardiac		
192			Cardiac		
Total recorded	70	116	6	74	35

Twenty-five individuals with a CV event recorded in the primary position also had an acute exacerbation of COPD (AECOPD) recorded in the primary position during the study period. In addition, 49 individuals with a CV event recorded in the secondary position also had an AECOPD recorded in the primary position during the study period.

For peer review only

Table 3 Conventional cardiovascular disease risk factors at baseline, their hazard ratios for cardiovascular disease.

	Median (IQR) or No. (%)	HR (95% CI)[a]	P value	HR (95% CI)[b]	P value
**Conventional CVD risk factors**					
Age – per 10 year increase	67 (62-73)	1.58 (1.30 to 1.92)	< 0.001	1.59 (1.29 to 1.99)	< 0.001
Sex – males	434 (61)	1.28 (0.94 to 1.74)	0.113	1.16 (0.84 to 1.60)	0.375
Smoking – current	218 (31)	0.95 (0.68 to 1.31)	0.744	0.92 (0.66 to 1.29)	0.632
HDL – per 1 mmol/L increase	1.4 (1.2-1.7)	0.73 (0.52 to 1.03)	0.074	0.86 (0.60 to 1.23)	0.402
Total cholesterol – per 1 mmol/L increase	5.0 (4.3-5.8)	0.80 (0.70 to 0.92)	0.002	0.89 (0.76 to 1.03)	0.118
SBP – per 10 mmHg increase	142 (131-154)	0.91 (0.84 to 0.99)	0.023	0.90 (0.82 to 0.98)	0.016
Diabetes – yes	82 (12)	1.50 (1.01 to 2.22)	0.044	1.15 (0.76 to 1.75)	0.511
Hypertension treatment – yes	245 (34)	1.79 (1.34 to 2.38)	< 0.001	1.76 (1.31 to 2.36)	< 0.001

Values are given as the median and interquartile range (IQR), or No. of cases (%). Baseline data of 714 patients are included. All models are stratified by recruitment site.
[a]Model includes age and sex.
[b]Model includes conventional cardiovascular disease risk factors: age, sex, smoking, high-density lipoprotein, total cholesterol, systolic blood pressure, diabetes, hypertension drug treatment.
CI = confidence interval. HDL = high-density lipoprotein. SBP = systolic blood pressure. CV = cardiovascular.

For peer review only

Table 4 Arterial stiffness at baseline, their hazard ratios for cardiovascular disease.

	Median (IQR) or No. (%)	HR (95% CI)[a]	P value	HR (95% CI)[b]	P value
**Measures of arterial stiffness**					
PWV – per 1 m/sec increase	9.8 (8.4-11.8)	1.01 (0.95 to 1.07)	0.796	0.99 (0.93 to 1.06)	0.749
CIMT – per 1 mm increase	0.81 (0.71-0.96)	1.09 (0.49 to 2.45)	0.827	1.08 (0.48 to 2.43)	0.861
AIx – per 5% increase	28 (20-34)	0.85 (0.77 to 0.93)	< 0.001	0.90 (0.82 to 0.99)	0.027

Values are given as the median and interquartile range (IQR), or No. of cases (%). Baseline data of 714 patients are included. All models are stratified by recruitment site.
[a]Model includes age and sex.
[b]Model includes conventional cardiovascular disease risk factors: age, sex, smoking, high-density lipoprotein, total cholesterol, systolic blood pressure, diabetes, hypertension drug treatment. Carotid intima-media thickness further included systolic blood pressure. Carotid-femoral aortic pulse wave velocity further included mean arterial pressure and resting heart rate. AIx further included resting heart rate and height.
CI = confidence interval. PWV = pulse wave velocity. CIMT = carotid intima-media thickness. AIx = augmentation index.

For peer review only

Table 5 Alternative measures at baseline, their hazard ratios for cardiovascular disease.

	Median (IQR) or No. (%)	HR (95% CI)[a]	P value	HR (95% CI)[b]	P value
**Other risk factors**					
CRP – per twofold increase	1.21 (0.47 to 2.01)	1.22 (1.07 to 1.39)	0.002	1.20 (1.06 to 1.37)	0.005
Fibrinogen – per twofold increase	1.22 (1.06 to 1.36)	2.27 (1.20 to 4.31)	0.012	2.12 (1.10 to 4.07)	0.024
Glucose – per twofold increase	1.59 (1.50 to 1.69)	1.18 (0.46 to 2.99)	0.730	0.83 (0.32 to 2.18)	0.709
BMI – per 1 kg/m^2 increase	27 (23-31)	1.04 (1.01 to 1.06)	0.002	1.02 (0.99 to 1.05)	0.089
GOLD – per 1 stage increase	1 (1-2)	1.22 (0.98 to 1.52)	0.071	1.29 (1.03 to 1.61)	0.026
4MGST – per 1 second increase	4.2 (3.5 to 5.2)	1.09 (1.03 to 1.16)	0.002	1.07 (1.01 to 1.14)	0.020
6MWT distance – per 30 metre increase	366 (255 to 440)	0.91 (0.88 to 0.94)	< 0.001	0.91 (0.88 to 0.95)	< 0.001
BODE – per 1 point increase	3 (1-5)	1.13 (1.07 to 1.20)	< 0.001	1.15 (1.08 to 1.21)	< 0.001

Values are given as the median and interquartile range (IQR), or No. of cases (%). Baseline data of 714 patients are included. All models are stratified by recruitment site.
[a]Model includes age and sex.
[b]Model includes conventional cardiovascular disease risk factors: age, sex, smoking, high-density lipoprotein, total cholesterol, systolic blood pressure, diabetes, hypertension drug treatment.
CI = confidence interval. CRP = C-reactive protein. BMI = body mass index. GOLD = global initiative for chronic obstructive lung disease. 4MGS = four-metre gait speed. 6MWT = six-minute walk test. BODE = body mass index, obstruction, dyspnoea, exercise.

For peer review only

Supplementary figures

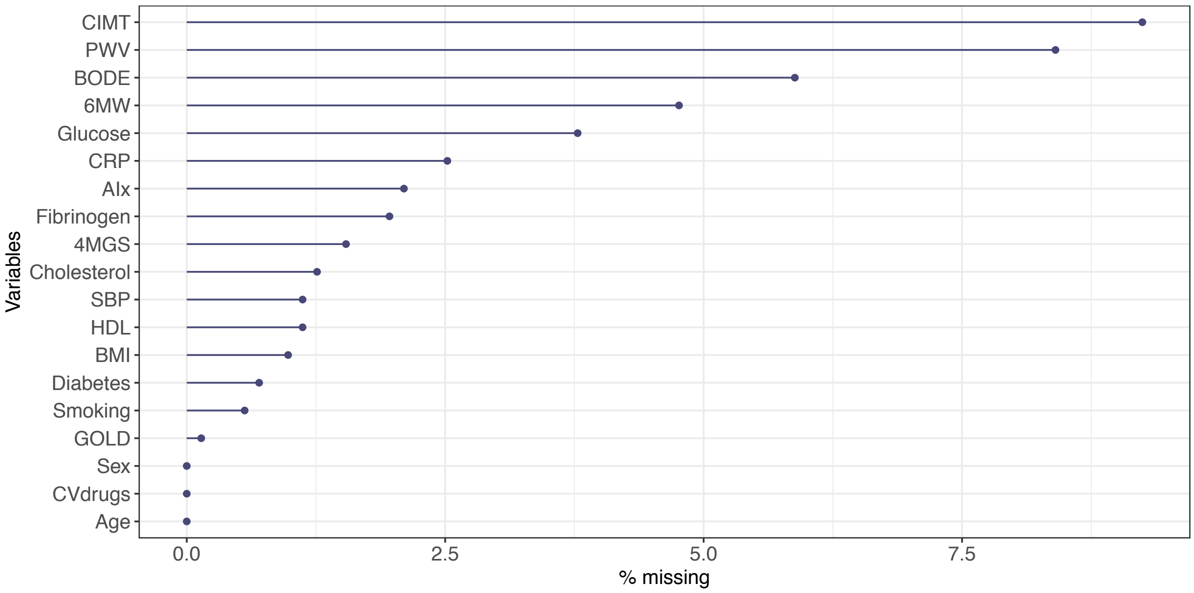

Figure 1 Percentage of missing values.
CIMT = carotid intima-media thickness. PWV = pulse wave velocity. BODE = body mass index, obstruction (i.e. forced expiratory volume in one second), dyspnoea score, exercise (i.e. six-minute walk test distance). 6MWT = six-minute walk test. CRP = C-reactive protein. AIx = augmentation index. 4MGS = four-metre gait speed. SBP = systolic blood pressure. HDL = high-density lipoprotein. BMI = body mass index. GOLD = global initiative for obstructive lung disease. CV = cardiovascular.

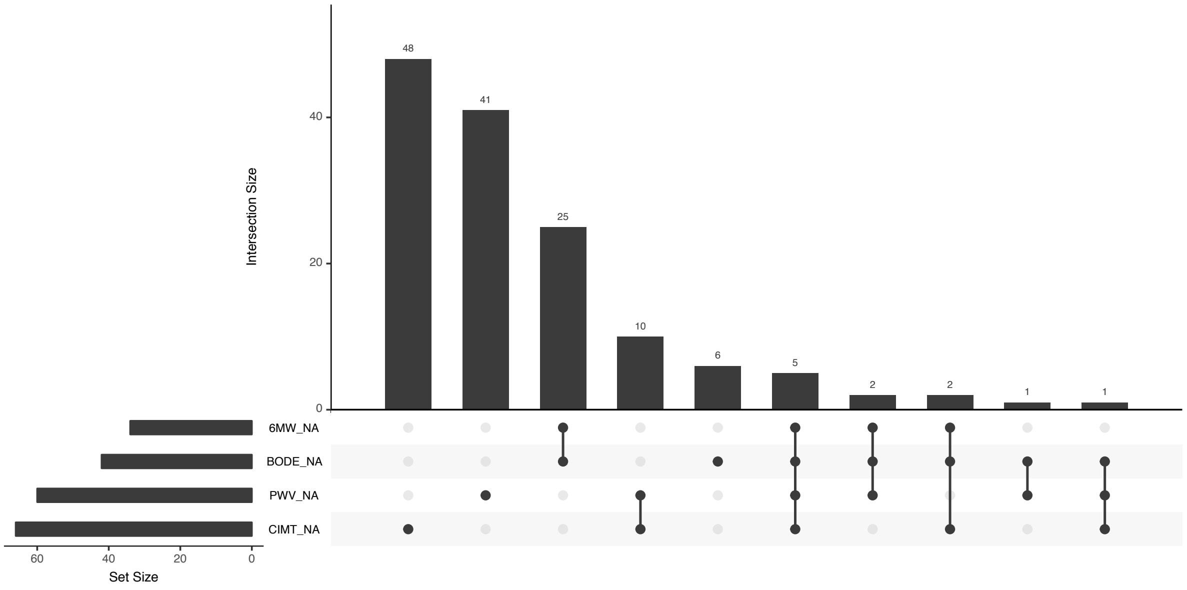

Figure 2 Percentage and pattern of missing values in key baseline characteristics.
6MWT, six-minute walk test. BODE = body mass index, obstruction (i.e. forced expiratory volume in one second), dyspnoea score, exercise (i.e. six-minute walk test distance). PWV, pulse wave velocity. CIMT, carotid-intima media thickness.

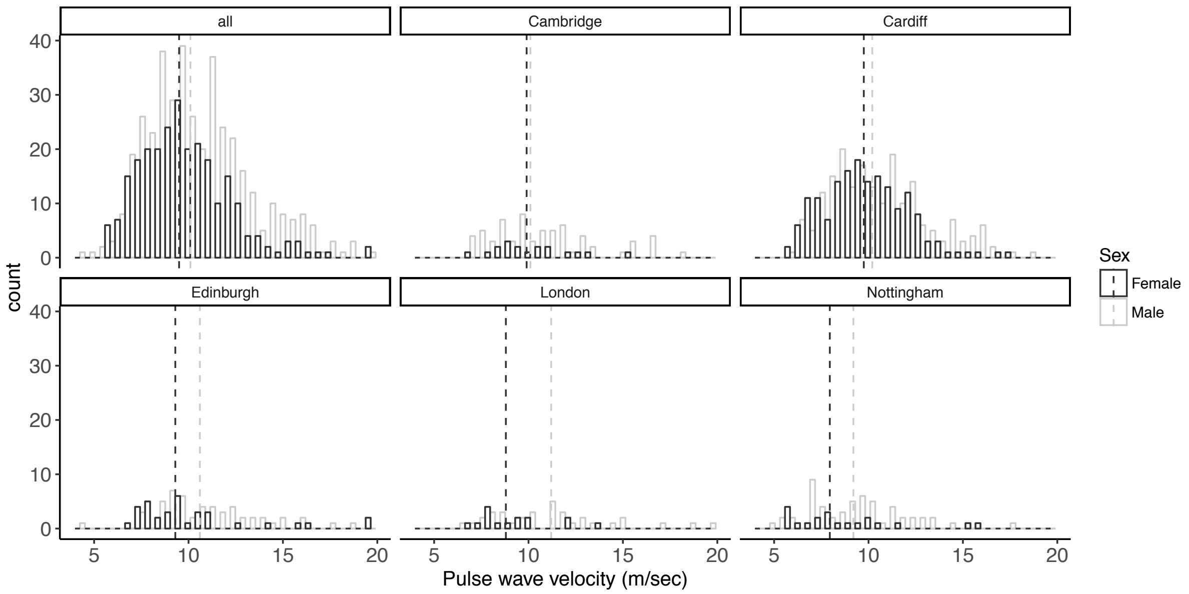

Figure 3 Differences in baseline pulse wave velocity.
Histograms displaying the distribution of PWV by sex and recruitment site. Dashed lines indicate median values by sex. Where only one median line is visible, medians for both sexes are similar.

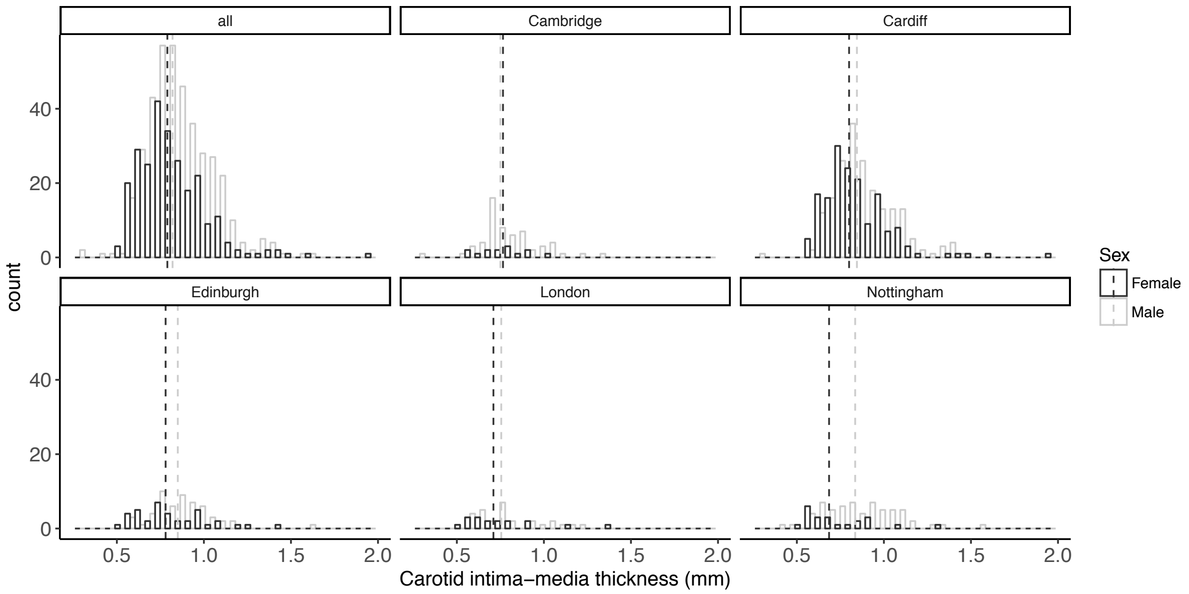

Figure 4 Differences in baseline carotid intima-media thickness.
Histograms displaying the distribution of CIMT by sex and recruitment site. Dashed lines indicate median values by sex. Where only one median line is visible, medians for both sexes are similar.

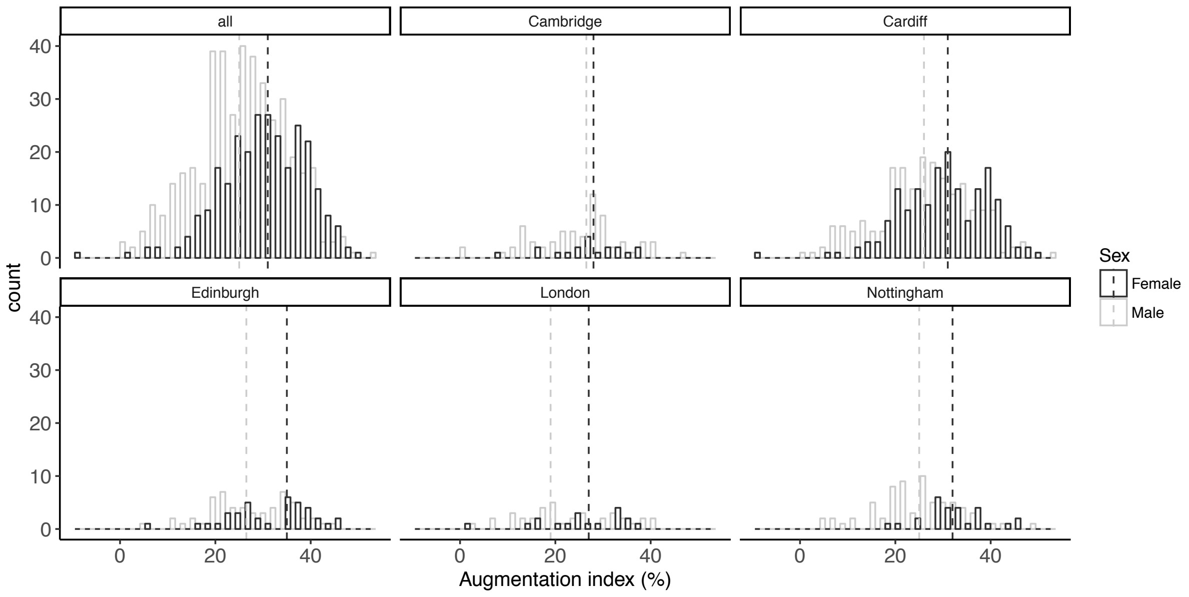

Figure 5 Differences in baseline augmentation index.
Histograms displaying the distribution of AIx by sex and recruitment site. Dashed lines indicate median values by sex.

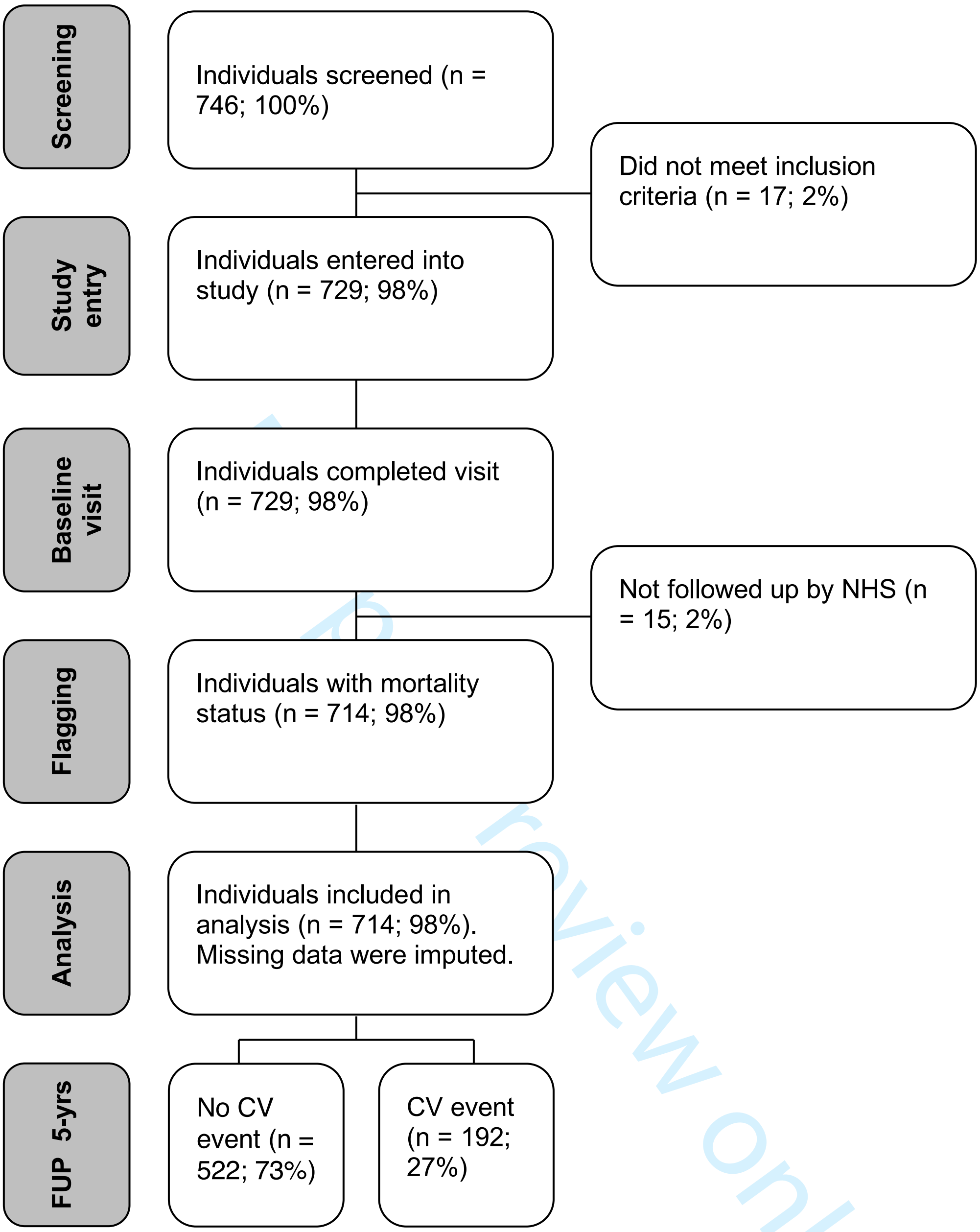

Figure 6 Participant enrolment flow diagram with five years follow up.
Total number of CV events (n = 192): peripheral arterial disease (n = 9), diseases of arteries, arterioles and capillaries (n = 7), angina (n = 21), unstable angina (n = 3), coronary heart disease not otherwise specified (n = 63), acute myocardial infarction (MI), and certain current complications following acute MI (n = 11), cerebral infarction (n = 11), stroke, not specified as haemorrhage or infarction (n = 3), other stroke (n = 18), heart failure (n = 32), abdominal aortic aneurysm (n = 8), and cardiac death (n = 6). FEV_1 = forced expiratory volume one second. FVC = forced vital capacity. NHS = National Health Services. FUP = follow-up period.

STROBE 2007 (v4) Statement—Checklist of items that should be included in reports of *cohort studies*

Section/Topic	Item #	Recommendation	Reported on page #
**Title and abstract**	1	(*a*) Indicate the study's design with a commonly used term in the title or the abstract	1
		(*b*) Provide in the abstract an informative and balanced summary of what was done and what was found	3
**Introduction**			
Background/rationale	2	Explain the scientific background and rationale for the investigation being reported	6
Objectives	3	State specific objectives, including any prespecified hypotheses	7
**Methods**			
Study design	4	Present key elements of study design early in the paper	8
Setting	5	Describe the setting, locations, and relevant dates, including periods of recruitment, exposure, follow-up, and data collection	8
Participants	6	(*a*) Give the eligibility criteria, and the sources and methods of selection of participants. Describe methods of follow-up	8
		(*b*) For matched studies, give matching criteria and number of exposed and unexposed	
Variables	7	Clearly define all outcomes, exposures, predictors, potential confounders, and effect modifiers. Give diagnostic criteria, if applicable	8-9
Data sources/ measurement	8*	For each variable of interest, give sources of data and details of methods of assessment (measurement). Describe comparability of assessment methods if there is more than one group	8-9
Bias	9	Describe any efforts to address potential sources of bias	9
Study size	10	Explain how the study size was arrived at	8-9
Quantitative variables	11	Explain how quantitative variables were handled in the analyses. If applicable, describe which groupings were chosen and why	9
Statistical methods	12	(*a*) Describe all statistical methods, including those used to control for confounding	9 + supplementary text
		(*b*) Describe any methods used to examine subgroups and interactions	9
		(*c*) Explain how missing data were addressed	Supplementary text 1
		(*d*) If applicable, explain how loss to follow-up was addressed	

For peer review only

		(*e*) Describe any sensitivity analyses	
**Results**			
Participants	13*	(a) Report numbers of individuals at each stage of study—eg numbers potentially eligible, examined for eligibility, confirmed eligible, included in the study, completing follow-up, and analysed	11 + supplementary figure 6
		(b) Give reasons for non-participation at each stage	Supplementary figure 6
		(c) Consider use of a flow diagram	Supplementary figure 6
Descriptive data	14*	(a) Give characteristics of study participants (eg demographic, clinical, social) and information on exposures and potential confounders	11-12
		(b) Indicate number of participants with missing data for each variable of interest	Supplementary figures 1-5
		(c) Summarise follow-up time (eg, average and total amount)	11
Outcome data	15*	Report numbers of outcome events or summary measures over time	11
Main results	16	(*a*) Give unadjusted estimates and, if applicable, confounder-adjusted estimates and their precision (eg, 95% confidence interval). Make clear which confounders were adjusted for and why they were included	12-14
		(*b*) Report category boundaries when continuous variables were categorized	
		(*c*) If relevant, consider translating estimates of relative risk into absolute risk for a meaningful time period	
Other analyses	17	Report other analyses done—eg analyses of subgroups and interactions, and sensitivity analyses	
**Discussion**			
Key results	18	Summarise key results with reference to study objectives	15
**Limitations**			
Interpretation	20	Give a cautious overall interpretation of results considering objectives, limitations, multiplicity of analyses, results from similar studies, and other relevant evidence	16-18
Generalisability	21	Discuss the generalisability (external validity) of the study results	18
**Other information**			
Funding	22	Give the source of funding and the role of the funders for the present study and, if applicable, for the original study on which the present article is based	19-20

*Give information separately for cases and controls in case-control studies and, if applicable, for exposed and unexposed groups in cohort and cross-sectional studies.

For peer review only

Note: An Explanation and Elaboration article discusses each checklist item and gives methodological background and published examples of transparent reporting. The STROBE checklist is best used in conjunction with this article (freely available on the Web sites of PLoS Medicine at http://www.plosmedicine.org/, Annals of Internal Medicine at http://www.annals.org/, and Epidemiology at http://www.epidem.com/). Information on the STROBE Initiative is available at www.strobe-statement.org.

For peer review only
